# HtrA1 Mediated Intracellular Effects on Tubulin Using a Polarized RPE Disease Model

**DOI:** 10.1016/j.ebiom.2017.12.011

**Published:** 2017-12-13

**Authors:** Esther Melo, Philipp Oertle, Carolyn Trepp, Hélène Meistermann, Thomas Burgoyne, Lorenzo Sborgi, Alvaro Cortes Cabrera, Chia-yi Chen, Jean-Christophe Hoflack, Tony Kam-Thong, Roland Schmucki, Laura Badi, Nicholas Flint, Zeynep Eren Ghiani, Fréderic Delobel, Corinne Stucki, Giulia Gromo, Alfred Einhaus, Benoit Hornsperger, Sabrina Golling, Juliane Siebourg-Polster, Francoise Gerber, Bernd Bohrmann, Clare Futter, Tom Dunkley, Sebastian Hiller, Oliver Schilling, Volker Enzmann, Sascha Fauser, Marija Plodinec, Roberto Iacone

**Affiliations:** a Roche Pharma Research & Early Development, Roche Innovation Center Basel, Basel 4070, Switzerland; b Department of Ophthalmology, Department of Clinical Research, Inselspital, Bern University Hospital, University of Bern, Bern 3010, Switzerland; c Biozentrum and the Swiss Nanoscience Institute, University of Basel, Basel 4056, Switzerland; d Institute of Ophthalmology, University College London, London EC1V9EL, United Kingdom; e Institute of Molecular Medicine and Cell Research, University of Freiburg, Freiburg D-79104, Germany; f BIOSS Centre for Biological Signaling Studies, University of Freiburg, Freiburg D-79104, Germany

**Keywords:** Age-related macular degeneration, Polarized human retinal, pigmented epithelium, HtrA serine peptidase 1, Disease modelling, Mechanical properties, Cell stiffness, Phagocytic activity

## Abstract

Age-related macular degeneration (AMD) is the leading cause of irreversible vision loss. The protein HtrA1 is enriched in retinal pigment epithelial (RPE) cells isolated from AMD patients and in drusen deposits. However, it is poorly understood how increased levels of HtrA1 affect the physiological function of the RPE at the intracellular level. Here, we developed hfRPE (human fetal retinal pigment epithelial) cell culture model where cells fully differentiated into a polarized functional monolayer. In this model, we fine-tuned the cellular levels of HtrA1 by targeted overexpression. Our data show that HtrA1 enzymatic activity leads to intracellular degradation of tubulin with a corresponding reduction in the number of microtubules, and consequently to an altered mechanical cell phenotype. HtrA1 overexpression further leads to impaired apical processes and decreased phagocytosis, an essential function for photoreceptor survival. These cellular alterations correlate with the AMD phenotype and thus highlight HtrA1 as an intracellular target for therapeutic interventions towards AMD treatment.

## Highlights

• RPE model to investigate the consequences of pathological HtrA1 overexpression
• Increase of HtrA1 transcriptional levels lead to tubulin intracellular degradation.
• Overexpression of HtrA1 leads to 2-fold reduction in stiffness of RPE cells.
• HtrA1 increase translates into impaired apical processes and phagocytic activity.

Age-related macular degeneration (AMD) is an eye disease which may result in blurred or no vision and it is associated with a malfunctioning retinal pigment epithelium (RPE) in the back of the retina tissue. The protein HtrA1 is enriched in RPE cells isolated from AMD patients. We reproduced a RPE cellular model which recapitulates the disease and reported how increased level of the HtrA1 protein leads to the impairment of RPE cellular functions and morphology. These cellular alterations correlate with the patient condition and suggest that inhibitors of the HtrA1 protein could have a therapeutic use in AMD.

## Graphical Abstract

**Figure:**
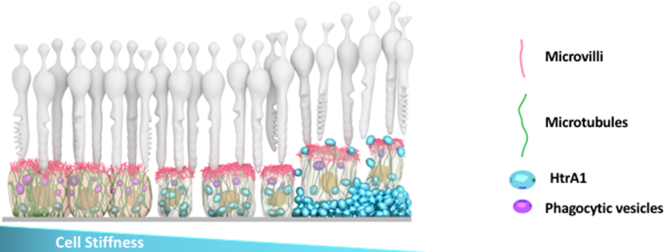

## 1 Introduction

Age-related macular degeneration (AMD) is the leading cause of irreversible vision loss in the elderly population (Coleman et al., 2008). Although the etiology of AMD still remains elusive, many studies have resolved that both genetic and environmental factors are involved in the onset of the disease. Among others, oxidative stress, smoking, UV radiation or high fat dietary intake account for the major environmental risk factors (Clemons et al., 2005, Schick et al., 2016). GWAS studies have identified several tightly linked polymorphisms on chromosome 10q26 that are strongly predisposing to AMD and affecting clinical outcomes to anti-VEGFA treatments (Fritsche et al., 2014, Fritsche et al., 2016, Grassmann et al., 2017, Tosi et al., 2017, Yamashiro and Mori, 2017). In proximity to this genomic locus there are two genes (*ARMS2* and *HTRA1*) which are the strongest AMD risk loci studied so far, though it remains contradictory how these genes influence each other and which are the causal role in the AMD risk (Cheng et al., 2013, Friedrich et al., 2015, Grassmann et al., 2017, Ng et al., 2017). One of these variants is located in the HtrA1 promoter region (rs11200638) (Dewan et al., 2006, Fritsche et al., 2016, Yang et al., 2006) and has been strongly suggested to have a putative role in the AMD pathogenesis. In support of this notion, transgenic mice overexpressing HtrA1 in RPE cells recapitulated features associated with advanced AMD and polypoidal choroidal vasculopathy (PCV) such as: degradation of the Bruch's membrane, increased damage of the Bruch's membrane upon exposure to cigarette smoke, and increase of PCV lesions (Iejima et al., 2015, Jones et al., 2011, Kumar et al., 2014, Nakayama et al., 2014, Vierkotten et al., 2011). HtrA1 is a member of the high-temperature requirement A family of the serine proteases and contains a homologous active domain to the bacterial serine protease which is responsible for the proteolytic activity (Eigenbrot et al., 2012). HtrA1 forms a trimer and its activation is driven by an allosteric mechanism of inter-monomer communication (Cabrera et al., 2017). It is also known that conversion by site-directed mutagenesis of the active domain Ser^328^ to Alanine (S328A) eliminates the enzymatic activity (Hu et al., 1998). HtrA1 was shown to play an important physiological role in extracellular matrix homeostasis and to cleave a substantial number of its components, such as fibronectin, type III collagen, decorin, aggrecan but also intracellular proteins such as tubulin (Canfield et al., 2007, Chien et al., 2004, De Luca et al., 2003, Schmidt et al., 2016).The HtrA1 protein has been reported to be present in drusen deposits, a hallmark of AMD pathogenesis, and within RPE cells isolated from AMD patients, albeit in a small cohort of patients (Tuo et al., 2008). In our work, we show clinical data from 10 post-mortem retinas extracted from 5 AMD patients and 5 healthy subjects that suggest an overexpression of HtrA1 in AMD and particularly an overexpression in the RPE layer of AMD patients. Despite different studies which aimed to define the role of HtrA1 in human RPE at the molecular level (Saini et al., 2017, Supanji et al., 2013), it remains to be further investigated how elevated levels of HtrA1 affect the physiological function of the RPE. This is partially due to the dearth of polarized cell culture models that accurately preserve the RPE phenotype and which could serve as a relevant cell model to investigate the consequences of HtrA1 pathological overexpression. In this work we have established a highly reproducible RPE cell culture model where primary hfRPE cells fully differentiated into a highly polarized and functional monolayer. Using this model and through an adenovirus mediated overexpression approach, we mimic the increase of the transcriptional levels associated with AMD predisposition and pathogenesis. By direct comparison between the overexpression of the proteolytically active HtrA1 protein and the enzymatically inactive S328A variant, which specifically neutralizes the HtrA1 proteolytic activity and leaves the rest of the protein unaltered, we provide intriguing molecular details of the mechanism by which HtrA1 activity leads to the intracellular degradation of tubulin. We also report the translation of this phenotype into impaired phagocytosis activity, a master function of the RPE ensuring photoreceptor survival. These findings delineate how induced HtrA1 could be contributing to the etiology of AMD and provide initial mechanistic insight into the intracellular role of HtrA1 in RPE cells.

## 2 Materials and Methods

### 2.1 Human RPE Cell Isolation and Culture

Human primary fetal retinal pigmented epithelium (hfRPE) cells were purchased from Sciencell (6540). Cells were seeded in transwells (Costar, 3450\3460) previously coated with Laminin 521 (BioLamina, LN521–03). During the first week EpiCM 2% FBS (Sciencell, 0010F,) media was employed to allow for attachment and proliferation (Sciencell, 4101). At day 4, FBS concentration was decreased to 1%. At day 7, cells were transferred to a maturation media; a modification of the formulation (MEM Alpha media (Sigma, M-4526) supplemented with N1 supplement at 1% concentration (Sigma, N-6530), Glutamine-Penicillin-Streptomycin 1 × (Sigma, G-1146), Non-essential Amino Acid (NEAA, M-7145), Taurine 0.25 mg/mL (Sigma, T-0625), Hydrocortisone 20 μg/L (Sigma, H-03966) and Triiodo-thyronin 13 ng/L (Sigma, T-5516) previously reported (Maminishkis et al., 2006, Sonoda et al., 2009). For the apical side of the transwells we used supplemented media 0.1% Bovine Serum Albumin (BSA) (Sigma, A-9647). For the basolateral side media with 1% FBS was employed. Barrier function was evaluated measuring the trans-epithelial electrical resistance (TER) from 2 wells in 3 independent experiments after 5 weeks of culture (CellZscope, NanoAnalytics). A mean value of 6 wells and SD was calculated.

### 2.2 *HTRA1* Overexpression, Constructs and Transfection

To mimic the increase of the *HtrA1* transcriptional levels in human RPE cells which have been associated with AMD, we used a recombinant adenovirus containing the human *HTRA1* mRNA (GenBank: NM_002775; SIRION Biotech) or an enzymatically inactive variant with a S328A modification. After 2 weeks in culture, when the RPE monolayer was completely established, cells were infected with the recombinant adenovirus encoding HtrA1, S328A or with a control adenovirus (Empty Vector). Cells were infected overnight at 37 °C at a multiplicity of infection (MOI) of 1. The medium was then changed, and the cells were kept in culture for three more weeks before any experiment was performed.

For some experiments, a variant from the same constructs was made with a HaloTag sequence added in the vector separated from the *HTRA1* by a linker sequence. We followed an infection protocol equal to the above described. Cells were also infected at the second week of growth and maintained for three more weeks.

### 2.3 HTRA1 and S328A Interaction Profiling by Immuno-Competitive Capture and Co-Immunoprecipitation

The HTRA1 immuno-competitive capture was performed as previously described (Meistermann et al., 2014). A commercial anti- HTRA1 antibody was used for IP and competition experiments (MAB2916, R&D, RRID:AB_212271) and western blot detection was performed with an in-house anti-HTRA1 antibody (Vierkotten et al., 2011). Anti-tubulin (MAB3408, RRID:AB 94650) was used for IP and (Ab52623, RRID:AB_869991) for blot detection. RPE lysate from overexpressing HTRA1 and S328A cells (500 μg total protein per condition) were pre-incubated for 1 h with increasing concentrations of free anti-HTRA1 antibody (0, 1, 2.5, 5 and 10 μg/mL) in triplicates.

Pre-incubated lysates were then loaded on a resin with immobilized anti-HTRA1 and incubated for 1 h. Eluates were separated on SDS-PAGE in three bands spanning from 20 to 120 kDa followed by in-gel trypsin. Samples were analyzed with a nanoflow Easy-nLC system (Proxeon) connected to an Orbitrap Fusion Tribrid mass spectrometer (Thermo Fisher Scientific). Raw files were then processed with Progenesis QI for proteomics (Nonlinear Dynamics) and searches were conducted with Mascot against a concatenated forward/reverse human database allowing for a spectrum false-discovery rate of 1%. Statistical analyses were performed in R as previously described (Meistermann et al., 2014). Briefly, after data quality control of identified peptide peaks, log2 scaled extracted ion counts (XIC) were normalized and summarized to relative protein abundance. To identify proteins displaced with increasing concentration of free anti-HTRA1 antibody, a linear model was fit using a set of contrasts (Augustin et al., 2013). The contrasts compare the protein abundance values above and below each concentration point. Then the maximum of the contrasts moderated t-statistics (Smyth, 2004) was obtained for each protein. Multiple testing adjusted significance (*p*-values) was derived by permutation testing*.*

### 2.4 Western Blotting

Cells were washed with PBS and lysed with RIPA buffer (Thermo Scientific) containing protease inhibitors (Roche Diagnostics GmbH, Mannheim, Germany). 200 μL cell media was precipitated with ice cold acetone at least 1 h at − 20 °C and then centrifuged at 16,000*g* for 10 min. Cell pellet proteins were then dissolved with RIPA buffer containing anti-protease. Samples (25 μg per cells, 20 μL per media) were then denatured in NuPage® LDS Sample buffer 4 × (Invitrogen, UK) at 70 °C for 10 min and run on commercially produced pre-cast 4–15% Criterion TGX Strain-Free gels (Bio—Rad) with Tris/Glycine/SDS (TGS) buffer (Bio—Rad). The proteins were transferred to a Trans-Blot® Turbo™ (Bio—Rad) membrane using the Trans-Blot® Turbo™ Transfer System (Bio—Rad) for 7 min. Membranes were incubated with 5% Blotting Grade Blocker Non-fat Dry Milk (Bio—Rad) in Tris-buffered saline (TBS) (Sigma) + 0.05% Tween-20 (Sigma) for 1 h at RT prior to incubation with primary antibodies specific to Tubulin (1:250, MAB3408; Millipore, RRID:AB_94650), HtrA1 (1:1000, (Vierkotten et al., 2011), Serpin F1/PEDF (1:250, AF1177, R&D Systems, RRID:AB_2187173), *E*-Cadherin (1:500, 610,182, BD Transduction Laboratories™, RRID:AB_39758), C1QTNF5 (1:250, MAB3167, R&D Systems, RRID:AB_2065810) with TBP (1:250, ab51841, Abcam, RRID:AB_945758) as the loading control, overnight at 4 °C. Immunodetection was performed by incubating the membranes with secondary antibodies IRDye® 680CW donkey anti-rabbit IgG (H + L), IRDye® 800CW donkey anti-mouse IgG (H + L), and/or donkey anti-goat (H + L) (1:5000, LI-COR Bioscience, Lincoln, NE) for 1 h at RT prior to washing with TBS + 0.05% Tween-20. Immunoreactive bands were visualized using the LI-COR Odyssey® infrared imaging system (LI-COR Bioscience, Lincoln, NE) according to the manufacturer's specifications.

### 2.5 Total RNA Preparation

Quadruplicate cell samples per time point (day 7, 14, 21, 28, 35) were available. After cell lysis and total RNA extraction in phenol/chloroform, the aqueous phase was processed using the RNeasy® Mini Kit (QIAGEN) as per the manufacturer's instructions. The quantity and quality of total RNA samples were assessed by spectrophotometric analysis (Thermo Scientific Qubit® 3.0 fluorometer) and by profiling on an Agilent Tapestation® 2200. The minimal sample amount was 1.61 μg with an average amount of 3.45 μg. The minimal RNA integrity number was 8.3 with an average of 9.52. Total RNA samples were normalized at a concentration of 80 ng/μL.

### 2.6 mRNA Sequencing

100 ng input total RNA per sample were converted into cDNA libraries following the Illumina TruSeq® stranded total RNA sample preparation protocol with Ribo Zero Gold (cat RS-122-2301). The total RNA, once depleted from cytoplasmic and mitochondrial ribosomal RNA, was fragmented and converted into double-stranded cDNA. Single-stranded adaptors that include unique barcode sequences were attached *via* ligation. The resulting molecules were amplified *via* polymerase chain reaction. The fragment size distribution of each library was quality-controlled using the Agilent Tapestation® 2200. The cDNA fragment size ranged 266–304 bp with an average of 280 bp. Libraries were quantified based on triplicate reactions of the Kapa® library quantification kit (Kapa Biosystems®, cat KK4835) using serial dilutions down to 1:8000. The concentrations ranged 11–98 nM with an average of 49 nM. The 20 libraries were normalized to 2 nM and pooled by 6 (including unrelated libraries) for each flow cell lane, as per the randomization plan. The pooled libraries were spiked in with 10% PhiX library and were bound to the surface of the flow cells at equimolar amounts of 11 picoM. Each template molecule was clonally amplified using a Cbot2® system (Illumina). Sequencing by synthesis was performed on the Illumina HiSeq2500®, using a strategy of 2x50bp paired-end sequencing. The total read depth ranged 16.8–27.3 million reads per sample mapped to the February 2009 human reference sequence hg19 (https://genome.ucsc.edu/cgi-bin/hgGateway?db=hg19), with an average of 22.3 million mapped reads.

### 2.7 miRNA Sequencing

1 μg total RNA of every sample was processed following the Illumina TruSeq small RNAseq library prep protocol (cat. RS-200-0012–0024). A single stranded 3’-RNA adapter and a single stranded 5’-RNA adapter were ligated to the total RNA of all samples. After reverse transcription, PCR-amplification of the resulting cDNA was performed using a unique primer pair for each sample. Each library was quantified before gel-purification based on Kapa® quantification (Kapa Biosystems®, cat KK4835) using 3 independent 10^− 6^ dilutions. The concentrations ranged 1.3–2.9 μg cDNA with an average of 2.1 μg. The fragment size distribution of each library was quality-controlled using the Agilent Tapestation® 2200. The 20 libraries were pooled and electrophoresed on a 10% acrylamide gel for purification of fragments spanning 140–160 base pairs. This fragment size covers the adaptor-ligated mature miRNAs, where the adaptors account for 120 bp. The content of this extract was quality-controlled using the Agilent Tapestation® 2200. The average library size was 150 bp. The concentration was determined with the Kapa® library quantification kit (Kapa Biosystems®, cat KK4835) using 3 independent 10^− 6^ dilutions of the gel-purified library pool. The concentration was normalized to 2 nM, spiked in with 10% PhiX library and loaded onto two flow cell lanes of a rapid HiSeq2500 run at a concentration of 13 pM. Each template molecule was clonally amplified using a Cbot2® system (Illumina). Sequencing by synthesis was performed on the Illumina HiSeq2500®, using a strategy of 50 bp single-end. The total read depth ranged 4.5–13.3 million reads per sample mapped to the February 2009 human reference sequence hg19 (https://genome.ucsc.edu/cgi-bin/hgGateway?db=hg19), with an average of 8.4 million reads.

### 2.8 Sequence Analysis

For mRNA and miRNA sequencing, the data were demultiplexed using Illlumina's CASAVA software, which converts the base call files into Fastq-files and simultaneously sorts the Fastq-files based on barcode information. Read depth per sample, sequence length distribution, Phred quality score, detection of duplicate sequences, GC content, distributions of base quality and base frequency per sample, were controlled before reads' mapping onto the human reference genome.

### 2.9 mRNA Sequencing Data Analysis

In order to estimate gene expression levels, paired-end RNAseq reads were mapped to the human genome (hg19) by using the short-read aligner GSNAP (Wu and Nacu, 2010, Wu and Watanabe, 2005). The number of mapped reads for all RefSeq transcript variants of a gene (counts) were combined into a single value by using SAMTOOLS software (Li et al., 2012) and normalized denoted as rpkms (number of mapped reads per kilobase transcript per million sequenced reads, (Mortazavi et al., 2008).

### 2.10 Micro RNA Sequencing Data Analysis

Adapter trimming on the raw micro-RNA sequence reads allowing up to one mismatch was performed by sRNABench (Barturen et al., 2014). Trimmed sequence reads of length 15 bp or more were then mapped onto hg19 including known micro RNAs (Griffiths-Jones, 2003) from miRBas with Bowtie (Langmead et al., 2009) using “genome mode” of sRNABench. Read counts and normalized reads counts per miRBase and novel micro-RNAs were generated by sRNABench.

### 2.11 Immunocytochemistry

Cells were fixed in 4% paraformaldehyde at room temperature, washed and permeabilized. Incubated primary antibodies were: Tubulin (1:500, MAB3408, Millipore, RRID:AB_94650), HtrA1 (1:500) (Vierkotten et al., 2011), α-acetylated Tubulin(1:500, T6793, Sigma, RRID:AB_477585), α-acetylated-Tubulin (1:800, 5535P, Cell Signaling Technology, RRID:AB_10548757) Serpin F1/PEDF (1:200, AF1177, R&D Systems, RRID:AB_2187173), *E*-Cadherin (1:200, 610,182, BD Transduction Laboratories, RRID:AB_397581), N-Cadherin (1:200, NBP1-48309, Novus Biologicals, RRID:AB_10011059), Claudin 19 (1:100, sc-162,687, Santa Cruz Biotechnology, RRID:AB_10918628) and Occludin (1:200, 331,500, LifeTechnologies, RRID:AB_2533101), Anti-Na +/K + ATPase alpha-1, clone C464.6 (1:500, Millipore, 05–369, RRID:AB_309699). After washing, samples were incubated with donkey Alexa-fluor 488/647 or Cy3 coupled antibodies (1:200, Jackson) and DAPI was added to the samples (LifeTechnologies). Samples were mounted with non-hardening mounting medium (H-1000, Vector Shield).

### 2.12 Procurement of Retina Tissue and IHC Staining and Imaging

#### 2.12.1 Post-Mortem Retinal Tissue Samples Were Donated by Eugenda (Uni Koeln)

Criteria for patient classification were absence of disorders of choroid and retina for the control patients (referred in the text as healthy subject) or a confirmed diagnosis of dry AMD by Eye care professional (refereed in the text as AMD patient). Sections of 4 μm thickness were obtained from formalin-fixed, paraffin-embedded human eye tissue from 5 healthy donors and 5 AMD patients. An immunohistochemestry (IHC) staining protocol was performed with the automated IHC research slide staining system Ventana Discovery Ultra. Stainings were carried out utilizing protocols and reagents according to the instructions of the manufacturer. An anti- HtrA1 (1:500) (Vierkotten et al., 2011) was employed as a primary antibody. A secondary antibody anti-rabbit HRP conjugated antibody (VENTANA, 760-4311) was used to detect the primary antibody. As chromogen, the Discovery Purple Kit (VENTANA, 760-229) was employed. Nuclear counterstaining was performed with hematoxylin.

An Olympus system VS120 slide scanner with a dry 40 × objective was employed to acquire the images of the stained retinas.

### 2.13 Imaging and Analysis

Unless otherwise stated, the general protocol to image the samples was done employing a laser scanning confocal microscope Zeiss 710 with a 40 × (tile scan images in Fig. 5) or 63 × water immersion objective. For high resolution images (Fig. 3B), stacks were acquired every 130 nm and images were then deconvolved (Scientific Volume Imaging, Huygens) and plotted with Imaris (Bitplane Scientific).

Apical imaging of the living RPE cells (Fig. 5, Fig. 6) was performed using an upright microscope (LSI, Leica) equipped with a water immersion lens 63 × maintained at 37 °C and 5%CO_2_ conditions. Orthogonal views and 3D images were generated with the same software used for the acquisition.

### 2.14 Intracellular β-Tubulin Quantification

Intracellular RPE tubulin intensity was measured in cells overexpressing HTRA1. At least 9 images/experiment were acquired using the same acquisition parameters (3 independent experiments). Images were evaluated using Image J software analysis. A region of interest was drawn on the surface occupied by at least 14 HTRA1 positive cells/image. We analyzed ≥ 400 cells per condition. A mean intensity per condition was calculated with all ROIs on the β-tubulin channel staining.

### 2.15 Transmission Electron Microscopy

Samples prepared for the TEM were first fixed during 24 h at 4 °C with a fixative solution containing 2%PFA, 2.5% glutaraldehyde in 0.1 M phosphate buffer (pH 7.4). Samples were then washed in 0.1 M cacodylate (pH 7.4). Transwell membranes were removed from the inserts and with the aid of a scalpel; small squares (5x5mm) were cut. A post-fixation step was done with 2% Osmium tetroxide in 0.1 M cacodylat buffer pH 7.4 for 2 h at room temperature followed by a rinse with cacodylate buffer at pH 7.4. After dehydration steps in graded acetone series the membranes were flat-embedded in Epon812. Resin polymerization was performed overnight at 60 °C. After trimming the block, ultra-thin sections (90 nm thickness) were done with a diamond knife on an ultramicrotome (Reichert Ultracut S) and collected on 200 mesh Nickel grids. A final step was performed staining with 5% Uranyl acetate and lead citrate. Images were acquired in a JEOL1210 or JEOL 1010 transmission electron microscope.

For the immunogold detection of HtrA1, cells were fixed for 24 h in 1% PFA in 0.1 M phosphate buffer at 4 °C. Transwell membranes were removed from the inserts and with the aid of a scalpel; small squares (5x5mm) were cut. These pieces were then incubated overnight in a 2.3 M sucrose solution. The small squares were subsequently embedded in 12% gelatin. Sections 100 nm thick were cut at − 120 °C and collected in a mixture of 1:1 2.3 M sucrose/2% methylcellulose. Labeling was performed on the sections, as described previously (Slot et al., 1991). Images of the samples were acquired on a JEOL 1010 transmission electron microscope.

For microvilli quantification 10 cells per condition were quantified, as well as the length of the membrane. The number of microvilli per cell was normalized for the μm pf plasma membrane. A Wilcoxon Mann Whitney test was performed to assess for significance in the difference between S328A and HtrA1 overexpressing cells.

### 2.16 Scanning Electron Microscopy

Samples for SEM were prepared using standard procedures with fixation using 2.5% glutaraldehyde and 2.5% paraformaldehyde in 0.1 M cacodylate buffer pH 7.4 (11,650, Electron Microscopy Sciences) for 1 h at room temperature, then rinsing in cacodylate buffer 0.1 M pH 7.4, and dehydration in an ethanol gradient. Samples were further dehydrated in ethanol gradient, critical point dryed (Critical Point Dryer- Tousimis Autosamdri 815), then sputter coated with 20 nm gold (Leica EM ACE600-Doube Sputter Coater) and analyzed using a SEM - FEI Nova Nano SEM230 or SEM - FEI Helios Nano Lab 650.

### 2.17 Atomic Force Microscopy (AFM)

All AFM experiments were carried out under close to physiological conditions using a customized mechano-optical microscope (MOM) comprised of an AFM (JPK Instruments AG, Germany and SPECS Zurich GmbH, Switzerland) and epifluorescence/spinning disk confocal setup (Visitron Systems GmbH, Germany). Bright field and fluorescence images were taken with 40 and 63 x oil immersion objectives (Leica, Germany) and recorded using an ORCA Flash 4 sCMOS (Hamamatsu Photonics K.K., Japan). For AFM experiments standard rectangular HQ-CSC38/CR-AU B cantilevers with a nominal spring constant of 0.03 N/m and a nominal tip radius of 20 nm (NanoAndMore GmbH, Germany) were used. The experimental value for the spring constant of each cantilever was determined by thermal tune method. For cell stiffness measurements, the AFM was operated in “Force-Volume Mode”. Briefly, arrays of force–displacement curves were recorded in a regular grid over a selected sample surface of 80 × 80 μm. Each of the 4096 (64 × 64) force–displacement curves per sample was sampled with 20 kHz. All indentations were performed with the maximum load set to 1.8 nN at an indentation velocity of 16 μm/s. Individual AFM stiffness map was completed within 45 min. For time-lapse and S328A/HtrA1 expression experiments, RPE cells were cultured on hanging insert membranes. For MOM experiments, the membranes were cut off from the insert and flat mounted onto an 18-mm coverslip. The coverslip was then inserted into a custom-made sample holder, which enabled correlative AFM and confocal measurements. To align optical and AFM measurements, bright-field and confocal images were recorded as z-stacks prior to each AFM measurement while the cantilever was contacting the sample surface, at the center of the AFM force map. During the measurements, culture dishes were replenished with fresh buffered DFCS saturated with 5% CO_2_ to maintain physiological conditions and compensate for evaporation. Post-AFM, RPE cells were fixed for further immunofluorescence analysis. AFM data were further analyzed using the custom-made “OfflineReader”, OriginPro 2016 (OriginLab Corporation, USA) as described previously (Plodinec et al., 2012) and Gwyddion (http://gwyddion.net/) software. The optical images were analyzed using Fiji/ImageJ (http://imagej.nih.gov). Histograms were plotted at a bin width of 7.5 Pa, mean-values and standard deviations were extracted from Gaussian fits onto the raw data. Statistical analysis was performed using two-sample student's *t*-test. Statistical significance was tested at *p* = 0.05.

### 2.18 Halotag Visualization and Tubulin Life Staining

To visualize the protease within the HaloTag-HTRA1 and HaloTag-S328A overexpressing cells we performed a rapid labelling protocol according to the manufacturer's instructions (Promega). Briefly, cells were incubated with the Halotag TMR ligand (G8251, Promega) at a concentration 5 μM during 30 min at 37 °C, washed twice with fresh media, incubated again for 30 min at 37 °C with fresh media and imaged using phenol red free fresh MEM (41,061, Gibco).

To stain for tubulin in living cells, samples were incubated for 6 h at 37 °C with SiR-Tubulin at a concentration of 200 nM (SC006, Spirichrome). Cells were then washed twice with fresh media and incubated with the Halotag ligand following the protocol previously described. Prior to imaging, cells were stained with Hoechst following the manufacturer's specifications (33,342, Lifetechnologies).

### 2.19 Cilia Quantification

RPE cells overexpressing HTRA1 or S328A were fixed and stained for tubulin. At least 9 images in 3 independent wells were acquired in confocal microscope with a 63 × water immersion lens. Images were then analyzed by Image J software. Maximum intensity projections from the z-stacks were generated and cells with at least 3 cilia were counted, as well as total cells (DAPI). ≥ 4000 cells were analyzed per condition and the percentage of ciliated population was calculated.

### 2.20 Phagocytic Challenge

Differentiated RPE cells were challenged with pHRodo-labelled red Zymosan bioparticles 100 μg/mL (Invitrogen, P35364) during 5 h. Samples were washed and stained with Hoechst (33,342, Lifetechnologies). Phagocytic activity was inhibited by Cytochalasyn D 0.2 μM (Sigma, C8273). Six images from 2 wells\experiment were acquired (3 independent experiments were conducted). Stacks were analyzed with Imaris software (Bitplane Scientific). Images were first subjected to a background substraction and then processed with the Spots module from Imaris. Particles of an estimated spot diameter of 800 nm were detected automatically and filtered based on a minimum satisfying intensity. Values higher than the set threshold were considered for analyses. Appropriate threshold values were confirmed by visual inspection of correct particle detection. Phagocytic activity was expressed as a percentage of the maximum activity, or that of the control cells.

### 2.21 POS Assay

Photoreceptor outer segments (POS) were isolated from freshly slaughtered porcine eyes as described (Schraermeyer et al., 1997). Isolated retinae were agitated for 2 min in KCl sucrose buffer and centrifuged at 7 k rpm for 5 min. Isolated POS were washed and labelled with CY5 Maleimide Mono-Reactive Dye (PA25031 Amersham GE Healthcare). Cells were incubated for 6 h at 37 °C with media containing labelled POS. Cultures were washed with PBS and immediately fixed. Nuclei were stained with Hoechst dye (33,342, Invitrogen). POS uptake was visualized by confocal microscopy (Zeiss LSM710; Carl Zeiss AG, Feldbach, Switzerland) ≥ 10 images\experiment; cells containing at least 3 particles where considered to be positive. Mean and SD were calculated from 2 independent experiments.

### 2.22 Terminal Amine Isotopic Labeling of Substrate (TAILS)

In order to find the potential intracellular proteins that could be regulated by HtrA1 activity, we performed a different approach: we inhibited the endogenous levels of this protease in control cells (not overexpressing any protein) with two HtrA1 serine protease inhibitors (Compound RI2404: N-[(2*S*)-3-(3-chlorophenyl)-1-[[(1*S*)-2-[[(3*S*)-5,5-difluoro-2-methyl-4,6-dioxo-6-(2,2,2-trifluoroethylamino)hexan-3-yl]amino]-1-(4-methoxyphenyl)-2-oxoethyl]amino]-1-oxopropan-2-yl]pyridine-2-carboxamide and Compound EM3770: N-[(2*S*)-3-(3-cyanophenyl)-1-[[(1*S*)-2-[[(3*S*)-5,5-difluoro-2-methyl-4,6-dioxo-6-(2,2,2-trifluoroethylamino)hexan-3-yl]amino]-1-(4-methoxyphenyl)-2-oxoethyl]amino]-1-oxopropan-2-yl]pyridine-2-carboxamide). Briefly, cells were grown following the described protocol and at day 35 media was replaced by serum free media containing the compounds at a concentration of 10 μM in 0.2% DMSO. Control cells, not containing any inhibitor were also treated with the same media containing 0.2% DMSO. Four days later, RPE cell lysates were harvested using 3 M guanidinium chloride plus protease inhibitors (1 μM E64, 5 mM EDTA and 1 mM PMSF) followed by centrifugation at 13,000 rpm for 10 min at 4 °C and TAILS was performed as described previously. Protein concentration was determined using bicinchonic acid assay. 0.5 mg of proteins per condition were labelled with either medium formaldehyde (^12^CD_2_O), sodium cyanoborodeuteride (NaBH_3_CN) or heavy formaldehyde (^13^CD_2_O), sodium cyanoborodeuteride (NaBD_3_CN) and then mixed in a 1:1 ratio. After tryptic digestion, internal peptides were captured by an amine-reactive polymer, whereas blocked N-terminal peptides were collected by ultrafiltration and desalted using reverse-phase C18 column. The sample was pre-fractionated by strong cation exchange chromatography and applied to liquid chromatography-tandem mass spectrometry (LC-MS/MS) analysis. LC-MS/MS data was acquired using the Q-Exactive plus system as described previously (Koczorowska et al., 2016). Data analysis and relative peptide quantitation was performed as described previously (Koczorowska et al., 2016) with the exception of lysine and N-terminal di-methylation (medium formaldehyde + 32.05 Da; heavy formaldehyde + 36.07 Da).

### 2.23 *In vitro* Microtubule Polymerization and HTRA1 Digestion

Hylite 488 fluorescent labelled microtubules (TL488M, Cytoskeleton) were polymerized following the instructions provided by the manufacturer. In brief, monomers of the microtubules were polymerized in 5 μL of GPEM buffer 10% Glycerol (Cytoskeleton, BST01, BST05, BT06) at 37 °C during 20 min. The polymerized microtubules were then resuspended in warm GPEM buffer 30% glycerol. HtrA1 recombinant proteins (Origene, TP322362) and S328A (Origene, TP700208) were added to a final concentration of 10 μg/mL. As a control for microtubule stabilization, we added Paclitaxel (Cytoskeleton, TXD01) to a final concentration of 20 μM. For each condition two replicates of 50 μL were generated and kept at 37 °C. Samples for observation were prepared by pipetting two microliters from each sample and mounting them between a slide and a coverslip. Five images from each well were taken at time points 5 min (just after adding the proteases), 6 h and 24 h. Images were acquired in a microscope (Zeiss, Observer Z.1) coupled to a fluorescence lamp and a camera (Insight, Spot 2Mpixel) using a 63 × water immersion objective. Images were analyzed with ImageJ using the tubeness plugin for tube-like structure detection. The analysis performs a gaussian convolution using a user defined sigma value that depends on the thickness of the tubes. A sigma value of 0.003 was selected as it produced the best results for our images in terms of polymers detection. The analyze particle plugin was used, providing a table with the number of objects found and their area. Values from each condition were plotted together in Graphpad as a histogram using a Bin Center of 0.5 μm2. This enabled us to obtain distribution of the microtubule size population and calculate the percentage occurring for a defined range of surface. We considered two have three types of events in our samples: small microtubules (area 0–0.75 μm2, bin center 0 and 0.5), that would account for segments of depolymerized microtubules, middle microtubules (area 0.75–3.25 μm2, bin center 1–3) and long microtubules (area ≥ 3.5 μm2, bin center ≥ 3.5). Statistical analyses were performed also in GraphPad Prism using a Mann-Whitney non-parametric test for two groups to compare samples treated with HTRA1 wild type protease or S328A HTRA1.

### 2.24 *In vitro* Microtubule Polymerization

An *in vitro* tubulin polymerization assay (BK006P, Cytoskeleton) was performed following the manufacturer's instructions. Briefly, a solution of tubulin monomers (2 mg/mL) was prepared and kept on ice using G-PEM buffer (80 mM PIPES, pH 6.9, 2 mM MgCl_2_, 0.5 mM EGTA, 1 mM GTP). Recombinant HTRA1 (Origene, TP322362) and HTRA1(S328A) (Origene, TP700208) were added to a final concentration of 10 μg/mL. Samples were then incubated for 30 min at 4 °C. Paclitaxel at a concentration of 10 μM was added in all conditions as a positive control. After incubation, samples were transferred to a warm plate and polymerization was monitored by absorbance at 340 nm during the following 90 min and keeping the plate reader at 37 °C. For each experimental condition, the signals from three independent replicates were averaged. Each signal was fitted by the sigmoid function with three free parameters

$$\left(t\right)=S_{\mathrm{rel}}\frac{1}{\left(1+e^{(-k_{F}\left(t-t_{0}\right)}\right)}$$

where *k*_*F*_ is the growth rate constant and *t*_0_ is the position of the inflection point of the curve and *S*_*rel*_ a relative signal amplitude. The latter was normalized to 1 corresponding to the largest occurring value among all conditions. The nucleation time point (*t*_*lag*_) is defined as the point when the tangent passing for the inflection crosses the time axis. It follows from the above parameters as:

$$t_{\mathrm{lag}}=t_{0}-\frac{S_{\mathrm{rel}}}{2k_{F}}$$

## 3 Results

### 3.1 Clinical Evidence of an Increased HtrA1 Content in AMD Patients

**Fig. 1:**
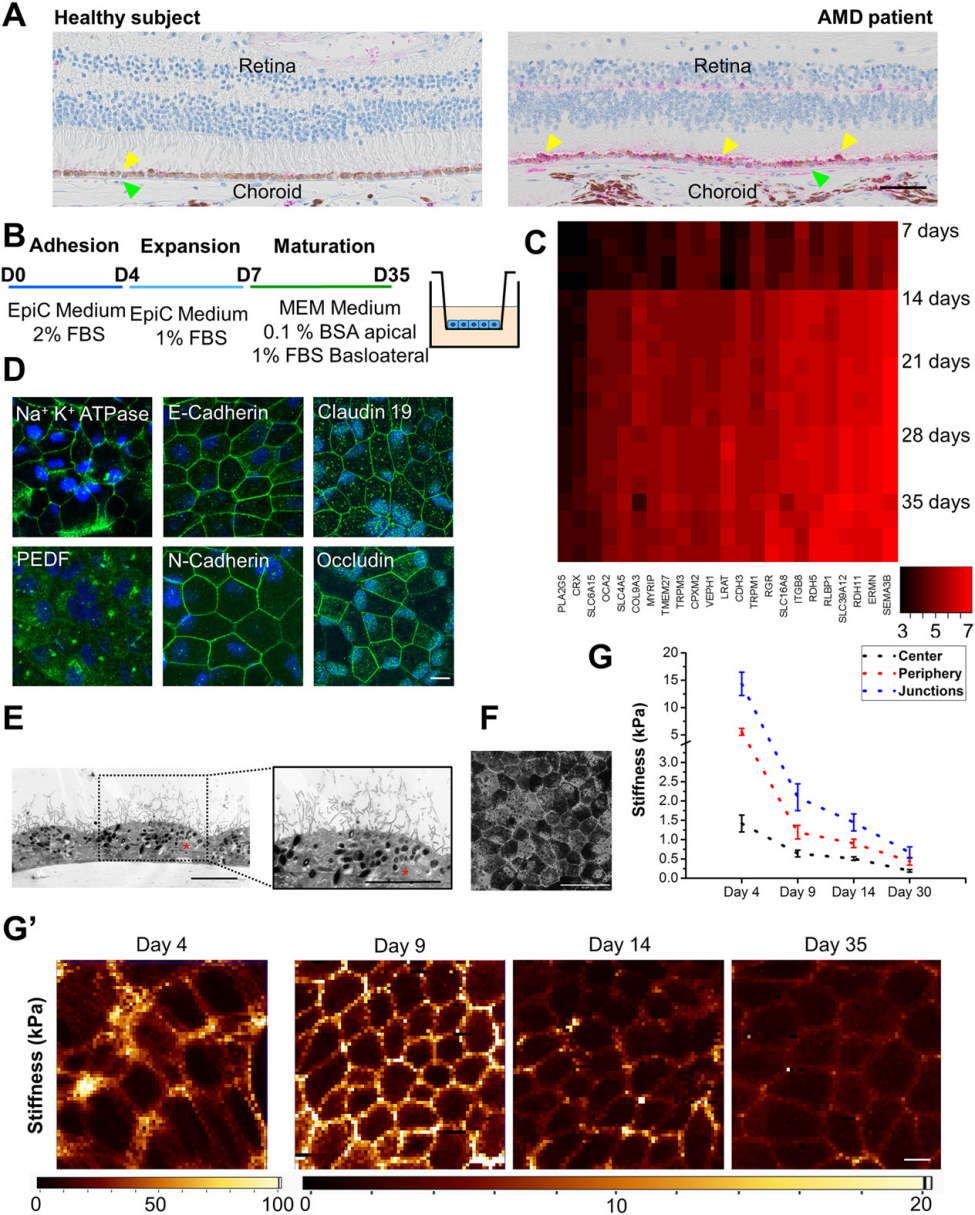
Polarized RPE cell model characterization. (A) Retinal histological sections immunostained against HtrA1 (pink) from a healthy subject (left) and an AMD patient (right). Nuclei stained with eosin. Yellow arrows indicate RPE cells. Green arrows indicate the BM (B) Differentiation protocol. (C) Panel of genes that by RNA sequencing experienced an increase along the differentiation time. (D) Representative images of several characteristic RPE markers: Na^+^ K^+^ ATPase, PEDF, E-cadherin, N-cadherin, claudin 19 and occludin (green); DAPI (blue); scale bar, 10 μm. (E) Transmission electron micrographs from hfRPE at day 35 of differentiation; scale bar, 5 μm. Asterisks indicate the nucleus (F) Phase contrast image of hfRPE cells at the end of the differentiation; scale bar, 50 μm. (G) Mechanical characterization along the differentiation at 4, 9, 14 and 35 days in culture. Mean values and ± SD for nuclei, cytoplasm and cell junctions from three independent experiments. (G′) Representative force-indentation maps at days 4, 9, 14 and 35 of culture; scale bar, 10 μm. See also Fig. S1.

To investigate the functional significance and tissue localization of the HtrA1 protein in human retinas form healthy subjects and AMD patients, we used Immunohistochemistry to re-confirm that HTRA1 is expressed intracellularly in the RPE layer and in the BM (Figs. 1A and S1) (Yang et al., 2006). The expression levels of the HtrA1 protein appeared higher in those samples corresponding to the AMD group, as seen by a stronger immunohistochemical HtrA1 staining and correlated with AMD-related structural changes in the retinal pigment epithelium (Bonilha, 2008). The RPE layer in the AMD patients presents a number of structural changes including: depigmentation due to the loss of melanin granules, disorganization of the basal infoldings, increase in the accumulation of basal deposits on or within Bruch's membrane, presence of drusen (between the basal lamina of the RPE and the inner collagenous layer of Bruch's membrane) and thickening of Bruch's membrane (Figs. 1A and S1). Unfortunately, our analysis of human retina tissues is limited to only 5 AMD patients' eyes and to 5 healthy subject eyes. However, these results represent an important base to further investigate the basic mechanisms induced by intracellular HtrA1 pathological overexpression and highlight the importance to develop a human polarized adult-like RPE model resembling the RPE layer in the retina tissue.

### 3.2 Characterization of a Human Polarized Adult-like RPE Cellular Model

Before investigating how increased levels of HtrA1 affect RPE functionality, we considered how to best employ RPE in our study. The putative role of HtrA1 in AMD pathophysiology has been associated with polarized and adult RPE, which have defined structural, molecular, and apical and basolateral polarized functionalities (Lehmann et al., 2014). We hypothesized that promoting RPE polarization features would represent a more physiological cellular model to investigate, at the molecular level, the consequences induced by HtrA1 pathological overexpression. In order to achieve a robust and reproducible cellular model, primary hfRPE were grown in PET transwell inserts following a protocol that involves conceptually three steps: adhesion; expansion and maturation (Fig. 1B). Maturation is promoted by using inserts previously coated with laminin 521, which is a major component of the Bruch's membrane and has been reported to effectively support the differentiation of pluripotent stem cells into RPE cells (Plaza Reyes et al., 2016). Moreover further optimization of the maturation medium was established by adapting a previously described RPE differentiation medium (Maminishkis et al., 2006, Sonoda et al., 2009).

To validate our protocol, we analyzed gene expression patterns at days 7, 14, 21, 28 and 35. Genes associated with the RPE signature could be divided in three clusters: genes increased during maturation (Fig. 1C), genes with consistently high expression (Fig. S1b, left), genes with consistently low expression (Fig. S1b, right). Interestingly, we could observe a drastic difference in the gene expression between days 7 and 14, coinciding with the switch to the maturation media. The genes increased during maturation and genes with consistently high expression include adult RPE markers such as: solute carriers (*SLC16A8*, *SLC39A12*, *SLC6A15 or SLC4A5*), genes related to the regeneration of visual pigments and completion of the visual cycle (*LRAT, RDH5, RDH 11, RLBP1, CRX*), genes associated with cell-cell and cell matrix adhesion (*CDH3, ITGB8 and COL9A3*), and well known RPE markers (*BEST1, SERPINF1, RPE65, RBP1, TIMP3, ADAM9 or VEGFA*). Interestingly, many of these genes were also identified and reported for hfRPE cells in a former transcriptomic analysis where different RPE types (fetal/adult and native/cultured) were compared (Strunnikova et al., 2010).

We also profiled the miRNA expression pattern at different time points and identified 7 miRNA (supplemental table S1) reported to be present in mouse and human RPE cells of different origin: RPE cells derived from either induced pluripotent stem cells (iPS) or embryonic stem cells (ESC) and primary RPE isolated from retina tissue. From the 7 dentified miRNA, 2 of them were also present in RPE from mouse: hsa-miR-30a-5p and hsa-miR-181a-5p (Ohana et al., 2015, Soundara Pandi et al., 2013). Two more miRNA matched profiles found in mouse and also human: hsa-miR-204-5p (Li et al., 2012, Ohana et al., 2015, Soundara Pandi et al., 2013) and hsa-miR-99b-5p (Soundara Pandi et al., 2013, Wang et al., 2014). A total of 3 miRNA were only reported to be present in human iPS-derived RPE cells: hsa-miR-27a-3p, hsa-miR-100-5p and hsa-miR-23b-3p (Wang et al., 2014). Together, these results suggest that the maturation step shifts the gene-expression profile of primary human RPE towards an adult-like pattern.

Characterization of the RPE phenotype at the end of the maturation was performed by several means. Polarization and an RPE signature could be confirmed by the immunostaining of several markers, such as Na^+^ K^+^ ATPase, Claudin 19, Occludin, E-cadherin, N-cadherin and PEDF (Figs. 1D and S1f). As well, we could confirm the characteristic epithelial polarization with clear microvilli located apically and nuclei present at the basolateral side of the cells by transmission electron microcopy (TEM) (Fig. 1E). Melanosomes could also be identified by TEM. Microscopic inspection revealed a confluent pigmented monolayer by the end of the protocol (Fig. 1F).

Additionally, we characterized the hfRPE monolayer by atomic force microscopy (AFM) at days 4, 9, 14 and 35 days. Polarized RPE cells were measured for first time by AFM when growing in a transwell setup (Fig. 1G). During the maturation period, a typical epithelial pattern of stiffness was found across the cell (Plodinec et al., 2012), cell-cell junctions were the stiffest region, the cytoplasmic area exhibited intermediate values, and the cell cytoplasm above the nuclear area the softest. We also found a general decrease in stiffness for all areas as maturation occurred, with the effect being stronger at the junctions than in the other two regions (Fig. 1G and 1G′) (Shawky and Davidson, 2015).

### 3.3 Modelling the Increase of the *HtrA1* Transcriptional Levels in RPE Cellular Models

Through direct comparison between the overexpression of the proteolytically active HtrA1 protein and the enzymatically inactive S328A variant, which specifically neutralizes the HtrA1 proteolytic activity and leaves the rest of the protein unaltered (Hu et al., 1998) we intended to reveal molecular details of the mechanism by which the HtrA1 enzymatic activity could lead to intracellular remodeling in RPE (Fig. S1c, schematic). Moreover, we confirmed that the percentage of transfected cells was the same for both conditions supporting the notion that any observed phenotypes are specific to the particular mutation and independent of the amount of virus transfected into the cells (Fig. S1c, left graph). Epithelial barrier function was assessed by measuring transepithelial electrical resistance (TER) revealing no differences between the HtrA1 and S328A cells (Fig. S1c, right graph). Achievement of high TER values by the end of the differentiation confirmed the presence of a tight barrier, characteristic of a healthy polarized epithelium.

To avoid any effects on proliferation, we transfected the RPE cells in the second week of growth when the monolayer achieved complete confluence and the cells were already undergoing maturation (Fig. S1d). After 5 weeks in culture, cells showed increased content of HtrA1 detected by Western Blotting (WB) at cellular levels but also in the apical and basolateral media (Fig. S1e). We detected a clear band at ~ 50KDa and observed auto-catalytic events, identified as bands of lower molecular weight, in cells and media from the HtrA1 overexpressing condition (Hu et al., 1998). We also observed a higher band ~ 80 kDa, which could represent a protein aggregate, since it is known that HtrA1 can form multimers (Eigenbrot et al., 2012). Taken together, these data show that our hfRPE model exhibits mechanical, molecular and expression/miRNA characteristics associated with a polarized adult-like RPE phenotype. Hence, this model constitutes a powerful tool to investigate the consequences induced by HtrA1 pathological overexpression by direct comparison between the proteolytic active HtrA1 protein and the enzymatically inactive S328A variant.

### 3.4 HtrA1 Interacts Biochemically with Tubulin in Polarized hfRPE Cells Leading to a Decrease in the Intracellular Tubulin Content

**Fig. 2:**
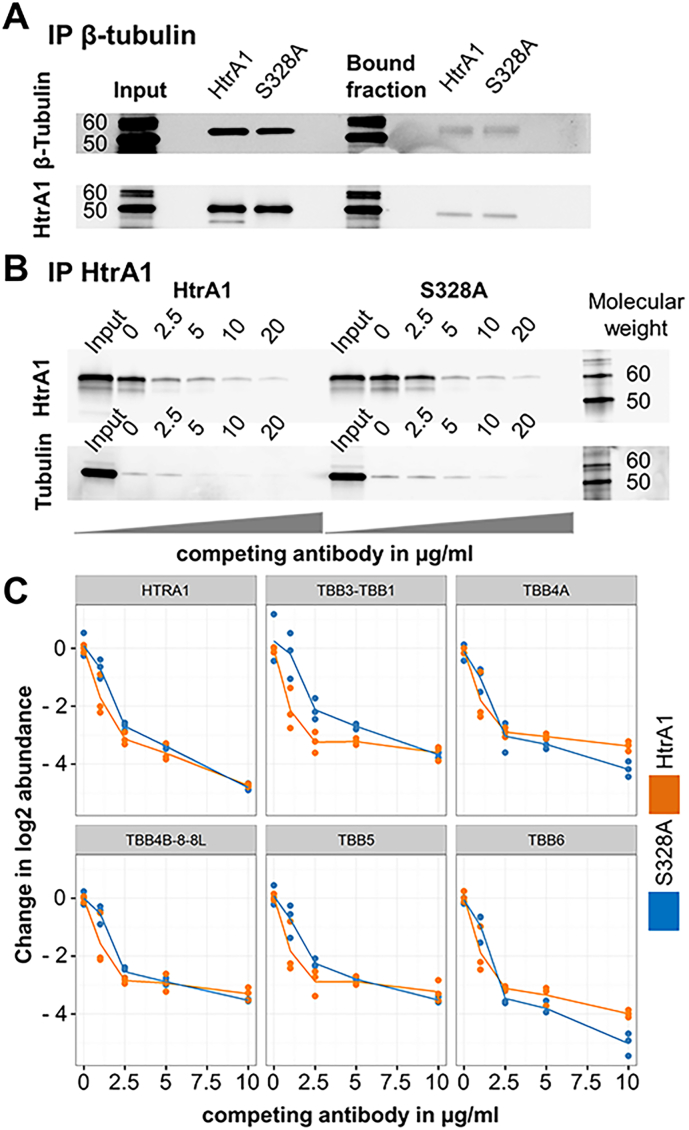
HtrA1 interacts with tubulin. (A) Immunoprecipitation against β-tubulin and detection of HtrA1 and β-tubulin in lysates obtained from HtrA1 and S328A HtrA1 overexpressing RPE cells. Left, input material in the column. Right, bound fraction. See also Fig. S2. (B) Western blot gels for HtrA1 and β-tubulin, representative of a single experiment from cell lysates incubated in increasing concentration of anti-HtrA1 competing antibody. Left, lysate from HtrA1 overexpressing cells. Right, lysate from S328A overexpressing cells. (C) Mass Spectrometry identification of possible HtrA1 interactors by immunocompetition assays. Concentration of HtrA1 and other beta subunits decay as the antibody competition antibody increases. Orange, lysate from HtrA1 overexpressing cells. Blue, lysate from S328A overexpressing cells.

To confirm the intracellular interaction between HtrA1 and microtubules (MT) as reported in previous studies (Chien et al., 2009b) we performed an immunoprecipitation (IP) using a beta-tubulin antibody in cell lysates from polarized hfRPE overexpressing Htra1 or S328A variant (Figs. 2A and S2a). We observed the co-immunoprecipitation (coIP) of HtrA1 irrespectively of the transfection conditions. Accordingly, beta tubulin was found to be co-immunoprecipitated when using an HtrA1 antibody for pull-down (Fig. 2B). Furthermore, using an Immuno-Competitive Capture (ICC) approach (Meistermann et al., 2014), we could validate the specificity of this interaction (Fig. 2B) as shown by a gradual decrease in the capture of beta tubulin upon increasing concentration of free competitor antibody. Combined with a mass spectrometry (MS) readout and robust statistical analysis, we showed that HtrA1 is interacting specifically with multiple beta tubulin subunits (Figs. 2C and S2b) as well as with alpha tubulin and multiple other candidates (supplemental table S2).

Additionally, we confirmed the cleavage of tubulin by HtrA1 in control cells by N-terminomics. We treated untransfected RPE cells with two selective HtrA1 inhibitors Compound RI2404 and Compound EM3770 which both display single-digit nM HTRA1 inhibition and selectivity (> 40 fold) towards three structurally related serine proteases of the chymotrypsin family Cathepsin S, Cathepsin G and Neutrophil Elastase (Fig. S2b). We observed, for both compounds, a more than two-fold down-regulation of three proteolytic cleavage sites in tubulin, each of which featured a hydrophobic P1 residue, in-line with biochemical HtrA1 specificity (Chien et al., 2009a, Truebestein et al., 2011) (Fig. S2c and Supplemental table S3).

Summarizing these results, we validate the interaction between HtrA1 and tubulin with high emphasis on the β subunits. In addition, we provide evidence of tubulin cleavage by HtrA1. These data indicate a probable role for HtrA1 as a microtubule associated protein (MAP) and its potential to regulate MT, tubulin stability and MT-associated cellular functions by reducing the amount of tubulin when active and expressed in high amounts.

**Fig. 3:**
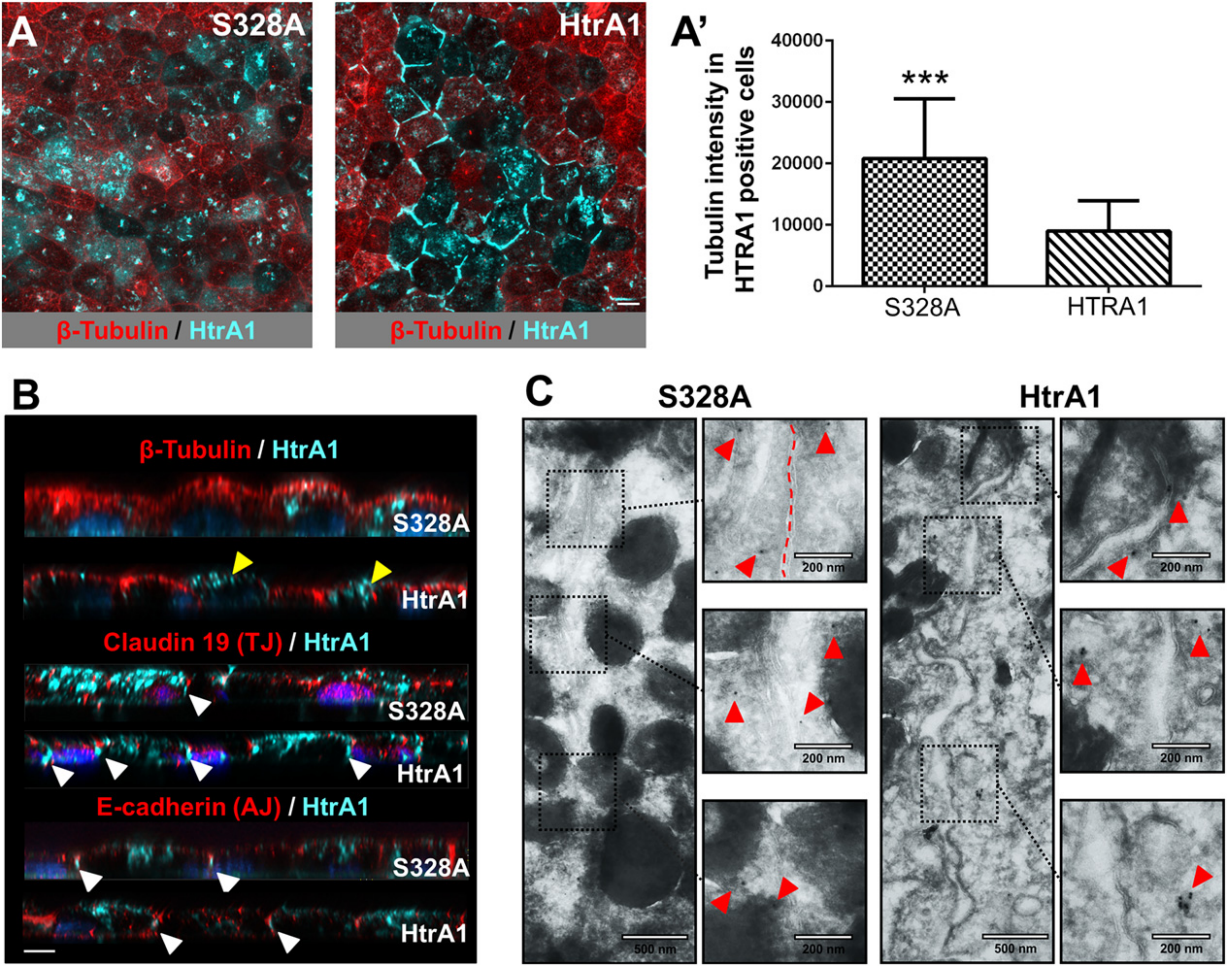
HtrA1 overexpression associates with tubulin loss and localization at the adherens junctions. (A) IF representative confocal image of S328A (left) and HtrA1 (right) overexpressing RPE at day 35 of culture. β-tubulin (red), HtrA1 (cyan) and DAPI (blue); scale bar, 10 μm. (A’) β-tubulin intensity quantification in HtrA1 positive cells at day 35 of culture. Data represent the mean ± SD of three independent experiments, ***p < 0.001. (B) IF high-resolution confocal images (*xy* plane) of tubulin, claudin-19 and E-cadherin (red) and HrA1 (cyan), DAPI (blue). The apical membrane faces the top. Yellow arrows indicate cells containing less tubulin. White arrows indicate localization of HtrA1 under claudin-19 and co-localization of HtrA1 and E-cadherin. Scale bar, 5 μm. (C) Transmission electron micrographs from immune-gold detection of HtrA1 in S328A, left panel, and HtrA1 overexpressing cells, right panel. Scale bars, 500 nm and 200 nm. Red arrows indicate gold localization. Dashed line indicates the limit between cells.

Having confirmed the biochemical interaction of HtrA1 with tubulin, we conducted an extensive cellular phenotyping to investigate the effect of HtrA1 enzymatic activity as well as its intracellular localization (control cells are in Fig. S3a). To this end, we performed immunostaining assays against HtrA1 in overexpressing RPE cells and found HtrA1 in the cytoplasm, below the primary cilium (Fig. S3b), and at the cell-cell junction level, especially for the catalytic active condition (Fig. 3A). Next, we deconvolved high resolution images and plotted the cross-sections of polarized RPEs. We could observe HtrA1 localized beneath Claudin-19 and at the level of *E*-Cadherin, suggesting that HtrA1 might be confined at the level of adherens junctions but not tight junctions (white arrows in Fig. 3B, Fig. S3c for an apical and basal view). Further immunodetection with gold particles confirmed deposition at cell-cell junctions for both HtrA1 and the S328A variant (Figs. 3C and S3d).

We noticed that RPE transfected with HtrA1 partially lost their typical microtubule distribution while S328A overexpressing cells continued to have this cytoskeletal structure (Fig. 3A). When comparing the tubulin intensity of the S328A *versus* HtrA1 positive cells, we detected an intensity reduction of greater than two-fold in the cells expressing the active protease (*p* < 0.001; Fig. 3A′).

Moreover, as an internal control, we observed the presence of tubulin at the cytoplasm in neighboring non-transfected cells. Very similar findings were reported in OV202 cells (human ovarian carcinoma cell line resistant to cisplatin) but with α-tubulin staining (Chien et al., 2009a). Images from the same samples acquired at high resolution and subsequently deconvolved, confirmed this observation (Fig. 3B). Indeed, we observed a homogeneous apical tubulin staining in the S328A overexpressing RPEs. In contrast, HtrA1 overexpressing cells lost partially the staining in the apical side (yellow arrows in Fig. 3B).

### 3.5 The Overexpression of HtrA1 Alters the Nanomechanical Properties of RPEs

**Fig. 4:**
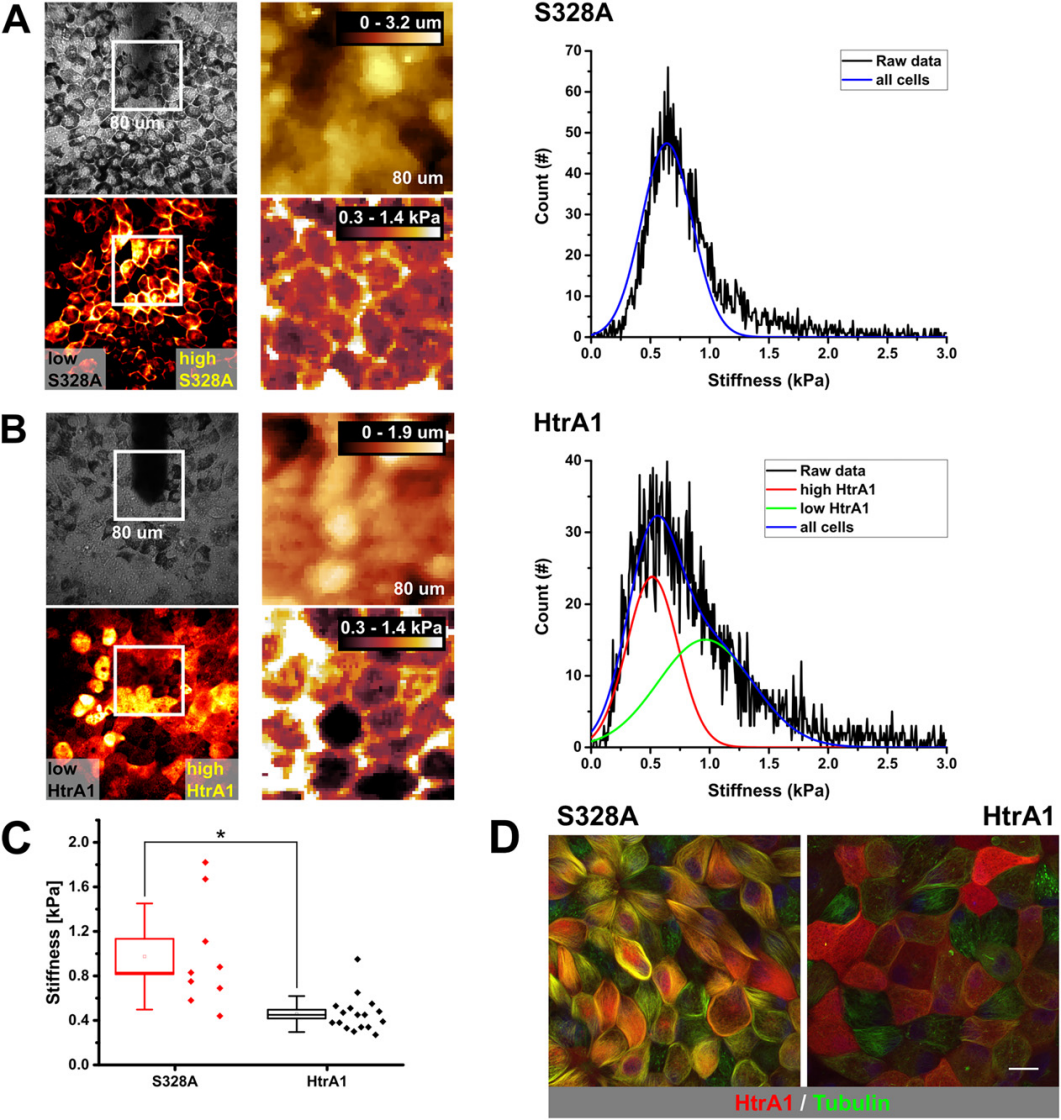
The nanomechanical fingerprint of RPE cells overexpressing HrA1 and S328A variant. (A/B, left panels) The cell viability and position of the AFM probe is monitored in the brightfield signal for each experiment. Live-Fluorescence imaging allowed for identifying the overexpressing cells (white insert). AFM topography and stiffness data belonging to the measurements (white insert). (A, right panel) Stiffness data, S328A variant cells exhibit similar properties when compared to the neighboring negative cells. (B, right panel) Stiffness data, HtrA1 overexpressing cells showed a 2-fold decrease for RPEs overexpressing HtrA1 (red line) when compared to cells having a low HtrA1 content (green line). (C) Quantitative stiffness analysis over multiple measurements. *N* = 9 maps (*n* = 92 cells) for S328A and *N* = 17 maps (*n* = 168 cells) for HtrA1, *p < 0.05. (D) Live cell imaging Halotag TMR ligand for HtrA1 (red), SiR-Tubulin (green) and Hoechst (blue). Scale bar, 20 μm.

In order to examine the contribution of the HtrA1 overexpression to the mechanical response in living RPE cells, we used an AFM setup with integrated light microscopy features allowing the AFM tip to be positioned to scan a defined area of interest. An essential feature of the combined microscope is the option of epifluorescence (Fig. 4A and B), which allows the overlay of corresponding fluorescence and bright field images, as well as their direct correlation with AFM stiffness maps. To specifically address the role of the HtrA1 in cellular elasticity, we have fluorescently labelled living cells with HtrA1 wild type and variant (S328A) constructs and examined their effects on the endogenous microtubule network and stiffness response. Quantitative analysis of the stiffness data (Fig. 4C) has revealed that enzymatically inactive S328A variant does not influence the nanomechanical response of cells while the overexpression of HtrA1 lead to 2-fold decrease in stiffness, impacting the organization of the endogenous tubulin cytoskeleton system (Fig. 4D).

### 3.6 HtrA1 Overexpression Translates into Reduced Apical Processes

To further investigate the repercussions of the partial tubulin deficiency, we hypothesized that other functions where tubulin is implicated could also be affected by HtrA1 overexpression. Aside from its role as a major cytoskeletal component, tubulin is also involved in the formation of the primary cilium found in RPE cells (Sloboda and Rosenbaum, 2007). In order to induce ciliogenesis, cells were grown without FBS in the apical compartment of the transwells (Tsang et al., 2008).

**Fig. 5:**
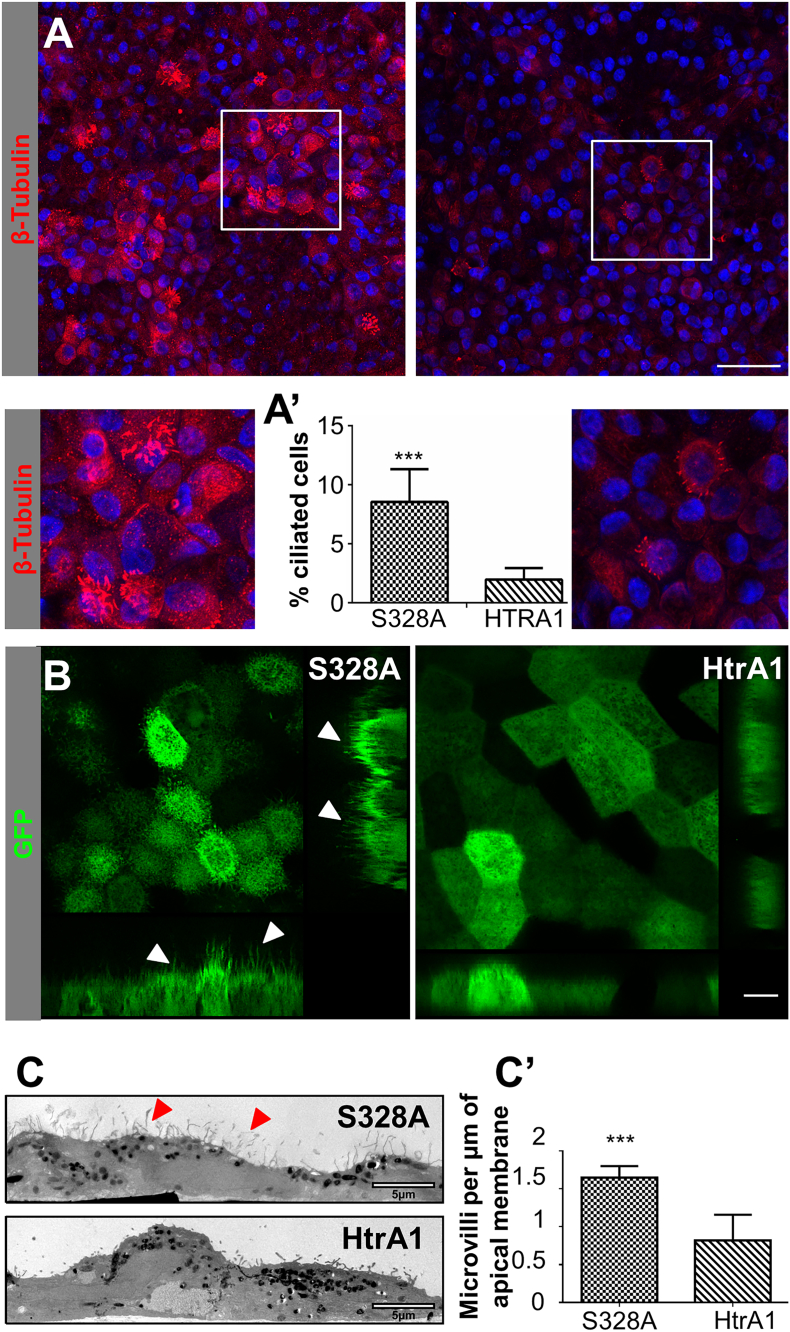
HtrA1 overexpression leads to apical impairment. (A) IF confocal images of RPE at day 35 of culture. β-tubulin (red) and DAPI (blue). Left image, S328A overexpressing cells; right image, HtrA1 overexpressing cells. Scale bar, 50 μm. Close up images show ciliated cells. (A′) % of ciliated cells in the RPE population. Data represent the mean ± SD of three independent experiments, ***p < 0.001. See also Fig. S3. (B) Life cell imaging of RPE cells at day 35 of culture. Left, S328A overexpressing RPE; right, HtrA1 overexpressing RPE. The apical membrane faces the top. Scale bar, 10 μm. (C) Transmission Electron micrographs of S328A (above) and Htra1 (below) overexpressing cells. Scale bar, 5 μm. (C′) Microvilli per μm of apical membrane. Data represent the mean ± SD of one experiment, ***p < 0.001.

To our surprise, when staining RPE cells for β-tubulin, we observed a distinct epithelial RPE population within the monolayer (Figs. S4c and S4b). Most cells had a primary cilium but some cells also appeared to have several cilia protruding from the surface (Fig. 5A). In addition, staining of RPE cells with α-acetylated tubulin, an appropriate marker of cilia, has confirmed the presence of multicilation and its co-localization with β-tubulin (Fig. S4a). These cells could also be observed when rendering the images of the stacks (Fig. S4b) or by Scanning Electron microscopy (SEM) (Fig. S4c). This result is intriguing, given the fact that RPE cells are not known to have multiciliation events. Interestingly, the cells that had been grown overexpressing S328A HtrA1 (Fig. 5A) had a higher proportion of these distinct, ciliated, RPE cells (8.45% ± 2.77%) than those that overexpress the WT HtrA1 (1.96% ± 0.98%). Differences between these two populations (Fig. 5A’) were found to be statistically significant (*p* < 0.001). Control cells grown with the empty vector had a similar population as the S328A cells (8.96% ± 1.9%). When growing the RPE cells without any viral transduction we did not detect any ciliated cells, suggesting that the viral transduction could be the trigger for this response. Naturally occurring or indirectly induced, healthy RPE cells are capable of developing cilia (S328A and EV) but not diseased RPE cells overexpressing the active protease (HtrA1).

Since the transduction vector expressed a GFP reporter cassette, we could image the RPE monolayer with a dip-in water lens that allowed us to image the surface of live RPE cells (Fig. 5B). Again, we could see a drastic difference between the apical processes (microvilli or cilia) found in the RPE from S328A cells as compared to the HtrA1. When the active protease was overexpressed we could barely detect any apical processes and cells appeared flatter compared to the S328A condition.

To better characterize the morphological differences observed between HtrA1 and S328A cells, the apical microvilli were quantified by TEM (Fig. 5C). A significant difference (p < 0.001) was found between cells overexpressing HtrA1 and S328A (Fig. 5C’). The examination by Scanning Electron Microscopy (SEM) (Fig. S3d) allowed us to compare the surface of the cells from a different perspective. Furthermore, we observed that S328A overexpressing cells had a rugose surface that was not found in HtrA1 overexpressing cells (Fig. S4d).

### 3.7 HtrA1 Overexpression Leads to Impaired Phagocytic Activity

**Fig. 6:**
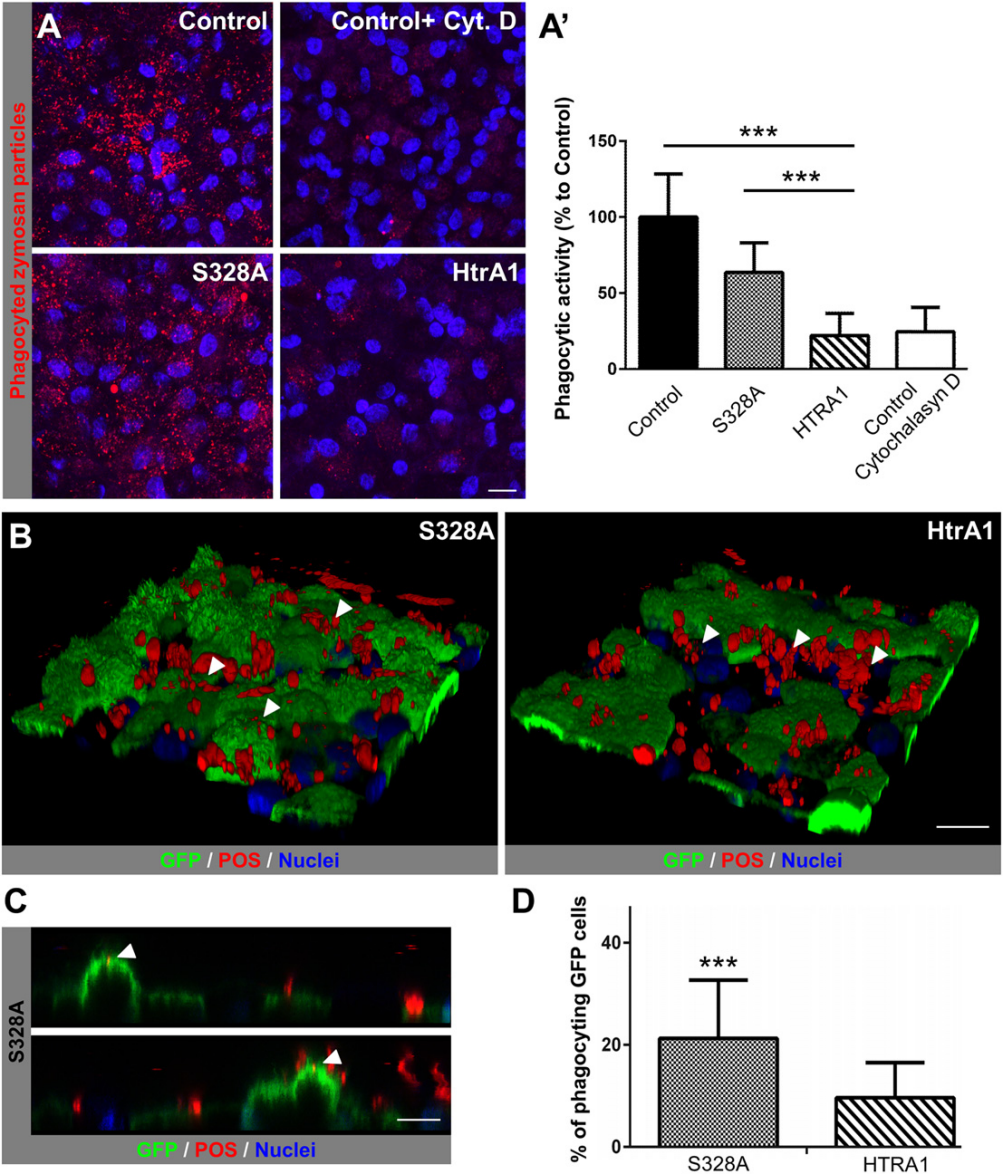
HtrA1 overexpression is accompanied by a decrease in the phagocytosis activity. (A) Representative confocal images of Zymosan A treated hfRPE from control cells, control cells treated with Cytochalasyn D, overexpressing S328A cells and HtrA1 overexpressing cells. Zymosan A particles (red) and Hoechst (blue). Scale bar, 10 μm (A′) Phagocytic activity (in % to control cells) from S328A overexpressing cells, HtrA1 overexpressing cells and control cells treated with Cytochalasyn D at day 35 of culture. Mean ± SD of three independent experiments, ***p < 0.001. (B) Life cell imaging of RPE cells while the process of phagocytosis. Left, S328A overexpressing cells; right, HtrA1 overexpressing cells. POS (red), GFP (green) and Hoechst (blue). Scale bar, 20 μm. (C) Close up image of S328A cells in the moment of particle uptake. POS (red), GFP (green) and Hoechst (blue). Scale bar, 10 μm (D) % of phagocyting GFP cells. Mean ± SD of two independent experiments, ***p < 0.001.

RPE cells are physically in contact with photoreceptor cells and can engulf the outer segments (Marmorstein et al., 1998)*.* Therefore, we hypothesized that diminished apical projections on RPE cells could lead to an impaired phagocytic activity. To assess for phagocytic performance, we challenged the RPE cells with synthetic particles (Fig. 6A) and further verified the results with labelled porcine outer segments (POS) (Fig. 6B–D). After incubation of RPE with opsonized Zymosan A, we observed that cells transfected with S328A variant had a reduced phagocytic function compared to the control cells (63.58% ± 19.49 of control) suggesting a possible effect from the transduction (Fig. 6A′). However, HtrA1 infected cells exhibited a more prominent reduction: 22.05% ± 14.5 compared to the S328A and control cells (*p* < 0.0001). This decrease was similar to that found in the control cells treated with Cytochalasin D (24.61% ± 15.94), a compound known to inhibit cell phagocytosis activity. Confirmation of these results was performed by challenging the RPE cells with labelled POS followed by assessment of the S328A and HtrA1 transfected cells (GFP positive) for POS uptake. The S328A GFP + population reached a phagocytic activity of 21.27% ± 11.41 compared to only 9.66% ± 6.8 observed in the cells overexpressing HtrA1 (Fig. 5D). This constitutes a statistically significant difference in phagocytosis activity between S328A and HtrA1 cells (*p* < 0.0001).

Upright imaging of the RPE monolayer during POS incubation allowed us to identify living cells engulfing particles. As predicted, we found POS particles in those cells with long processes (Fig. 6C). By plotting the images in 3D, we once again confirmed that HtrA1 cells had a smoother surface with fewer projections, as compared to S328A cells (Fig. 6B). Also, we found POS binding preferably to non-transfected HtrA1 GFP- cells (white arrows in Fig. 6B, right panel), which did not occur in S328A transfected cells where POS were found in the surface of both GFP + and GFP- cells (white arrows in Fig. 6B, left panel). Nevertheless, for quantification purposes (Fig. 6A′ and D), only those cells containing POS internalized were considered. To this end, cells were examined through the orthogonal projections ensuring that only those particles reaching the cytoplasm were considered (Fig. S5).

### 3.8 HtrA1 Aggregates and Degrades Microtubules *in vitro*

Previous studies have suggested a possible role for HtrA1 in stabilizing MT in addition to HtrA1 driven tubulin degradation that has been observed *in vitro* (Chien et al., 2009b). Our observations in RPE cells support the idea of HtrA1 degrading tubulin whilst also having a strong stabilizing effect. Since previous studies were conducted with tubulin monomers, we decided to perform experiments with polymerized MT where the protease would be in contact with the filament instead of the monomers, allowing us to assess if the same behavior occurred.

**Fig. 7:**
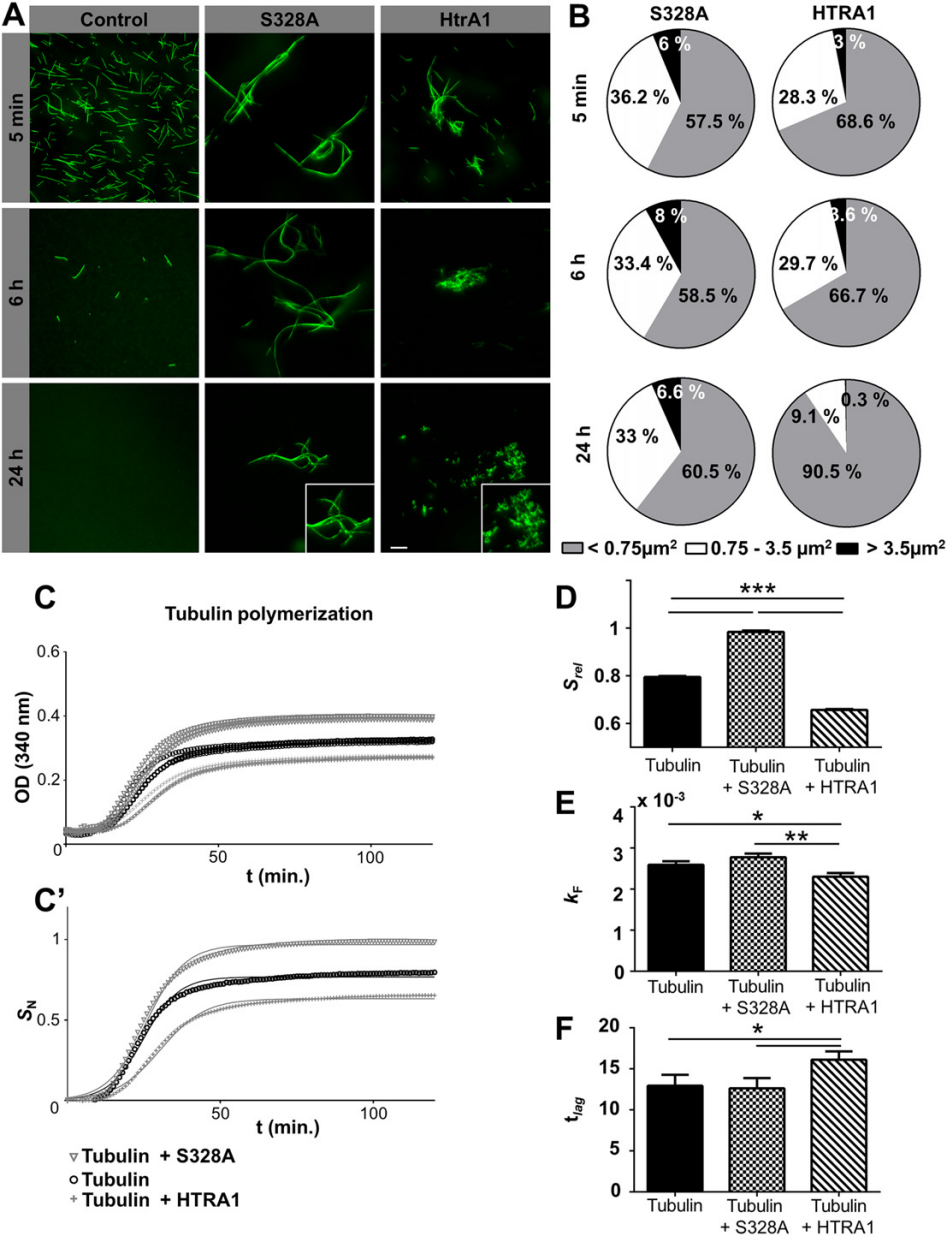
HtrA1 degrades preformed MT and avoids polymerization *in vitro.* (A) MT polymerized *in vitro* and incubated with S328A HtrA1 (center) and HtrA1 (right). Images acquired at 5 min, 6 h and 24 h after adding the protease. Scale bar, 10 μm. (B) Quantification of the area occupied by the MT 5 min, 6 h and 24 h after adding the protease. (C) Microtubule polymerization reactions in the absence or presence of S328A HtrA1 and HtrA1. As a representative growth signal, the absorbance at 340 nm was monitored *in vitro* as a function of time for three independent replicates. (C′) The average growth signal for each variant was calculated and normalized relative to the overall maximum. Best fits to sigmoidal function are shown with solid lines. (D) Comparison of the growth amplitudes (*S*_rel_), (E) kinetic rate constants (*k*_F_), (F) nucleation time (*t*_lag_) of microtubule polymerization reaction in the absence or presence of S328A HtrA1 and HtrA1. Mean ± SD for three replicates are reported, *p < 0.05, **p < 0.01, ***p < 0.001.

To this end, we incubated fluorescently labelled MT (16.7 μg/mL) with HtrA1 and S328A recombinant proteins (10 μg/mL) over 24 h and acquired images throughout the digestion. Just 5 min after the addition of the protease, a very strong aggregative effect was observed in both HtrA1 and S328A (Figs. 7A and S6a). After 6 h, aggregate formation was detected in the samples incubated with S328A and MT appeared to be longer. Aggregate formation was also seen in MT incubated with HtrA1 but, in contrast to S328A, no increase in length was seen. In the control without HtrA1 or S328A, the MT depolymerized, as the GTP was consumed, and a reduction in the number of MT present in the solution was observed. The lack of microtubule depolymerization observed in both the HtrA1 and S328A samples indicates that HtrA1 has a first stabilization effect on MT that is not dependent on protease activity. Interestingly, over time, MT incubated with HtrA1 began to show signs of degradation including retraction and condensation with the formation of thick aggregates. After 24 h, HtrA1 incubated samples contained no MT but, instead, clusters of digested tubulin. In contrast, at the end of the incubation no signs of microtubule degradation were observed in the S328A samples.

In order to quantify the events described, we measured the surface occupied by the MT at time points referred to above (Fig. 7B). Images were analyzed for the recognition of tube-like structures. We then clustered the distribution of the events in three different groups: long (> 3.5 μm2), medium (0.75–3.25 μm2) and small (shorter than 0.75 μm2). This analysis allowed us to see that samples incubated with the S328A protease had a similar pattern throughout the incubation time: 57.5–60.5% short MT, 33–36% medium sized MT, 6–8% long MT. When analyzing the MT that had been incubated with the HtrA1 protease, we observed an increase in the proportion of short MT (68.6% at 5 min, 66.7% at 6 h and 90.5% at 24 h), which is most likely due to degradation. In parallel, we observed a decrease in the proportion of medium (28.3% at 5 min, 29.7% at 6 h and 9.1% at 24 h) and long MT (3% at 5 min, 3.6% at 6 h and 0.3% at 24 h).

We also wish to emphasize that the difference in length between the MT incubated with HtrA1 and S328A protease were at all times present, having always a bigger proportion of long and medium size MT in the S328A samples as compared with the HtrA1 incubated MT. When performing a Mann-Whitney non-parametric test to compare the two groups, at the measured times, we obtained statistically significant differences (*p* < 0.001). Thus, diverse results can be read from this experiment: HtrA1 and S328A can stabilize MT and aggregate them in a first moment, with a stronger effect observed in S328A. Only MT incubated with HtrA1 are degraded over the time.

### 3.9 HtrA1 Impairs Microtubule Polymerization

Since formation of cilia requires polymerization of tubulin *de novo*, we decided to investigate if HtrA1 would have any effect on this process *in vitro*. Previous experiments reported that HtrA1 promotes the assembly and stability of the MT at low concentrations (15 ng/mL) but not at high concentrations (1.5 μg/mL) (Chien et al., 2009b). Based on our observations we found that S328A overexpressing cells formed new cilia but not HtrA1 cells. To fit with this result, we would expect HtrA1 and S328A to be present at high concentrations in the cytoplasm of the RPE cells. Therefore, we decided to perform our experiments at tubulin concentrations of 3 mg/mL with HtrA1 at 10 μg/mL. To maximize the interaction of the tubulin subunits with the recombinant proteins, we incubated the solutions at 4 °C for 30 min and then started the polymerization at 37 °C. As a control, we performed the experiments with and w/o taxol (MT stabilizer) for all conditions.

As shown in Fig. 7C/C′, when polymerizing the MT all conditions reached a plateau after around 60 min. Through comparison of the experiments performed with and w/o taxol, we can see that the effects are stronger in the presence of this compound (Fig. S6b). Samples incubated with S328A achieved the highest signals, whereas samples polymerizing in the presence of HtrA1 achieved the lowest (Figs. 7D and S6c, top). Signal values directly report the quantity of MT formed, meaning that S328A lead to the formation of more MT than the other two conditions (p < 0.001). The filament formation rate constant was identical within error between control and S328A polymerized samples, implying that S328A affects the amount of MT generated but not the polymerization kinetics (Figs. 7E and S6c, middle). MT polymerized with HtrA1 were significantly lower in quantity and also slower in their formation compared to S328A incubated (*p* < 0.01) or control (*p* < 0.05). Thus, HtrA1 appears to have an effect at both levels: quantity of MT formed and the speed at which they grow. Finally, assessment of the time at which enucleation started (lag time), revealed a marked difference between the conditions: S328A was the fastest to enucleate and HtrA1 the slowest. Statistically significant differences were found between the HtrA1 incubated samples and the other two conditions (*p* < 0.05) (Figs. 7F and S6c, bottom).

## 4 Discussion

The growing evidence indicates that HtrA1 plays an important role in AMD pathophysiology however no treatment has been developed to target this protease. The lack of therapies can be partially attributed to the limited understanding we have of AMD and, in particular, the absence of reliable cellular models. In this work, we introduce a polarized and physiologically relevant RPE cell-culture model system where the impact of HtrA1 overexpression on cellular phenotype and functions could be evaluated. Intracellular HtrA1 interactomics studies allowed us to identify several HtrA1 binding partners with a large proportion of candidates emerging from the tubulin protein family. By mimicking the disease with a native and enzymatically inactive S328A variant we have been able to compare their phenotypes and understand the effect of the intracellular increase in HtrA1 at a functional level. Overexpression of the active protease leads to apical dysfunction accompanied by decreased phagocytic activity, one of the most pivotal functions of polarized RPE cells.

We cultured highly differentiated human RPE cells by developing a 3-step protocol and provided several lines of evidence to show that the RPE cells matured towards the desired phenotype. Immunocytochemical characterization of tight/adherens junctions and TEM imaging confirmed a proper polarization of the cells. Moreover, by mRNA and miRNA profiling we described a typical RPE signature. Furthermore, we were been able to identify seven miRNA that were also described in the literature in human (Li et al., 2012, Wang et al., 2014) or mice RPE cells (Ohana et al., 2015, Soundara Pandi et al., 2013) from tissue or cell culture origin. Nevertheless, our work is the first to generate such profiles employing differentiated RPE cells from human fetal origin.

The mechanical characterization performed with AFM is also a novelty in the field. To date, the majority of mechanical measurements by AFM have been performed on cell lines (Haase and Pelling, 2015). We report, for first time, results acquired on primary epithelial cells that were cultured on transwell inserts. Mechanical measurements using a nanometer scale AFM force measurements have revealed a characteristic mechanical hallmark of healthy epithelia (Plodinec et al., 2012) with cell-cell junctions being predominantly stiffer than the cytoplasm or the perinuclear area. Moreover, we demonstrated that RPE cells soften during the differentiation protocol and especially at the junctional level. This time-lapse characterization was essential to obtain the baseline mechanical properties of RPE since we hypothesized that any effect of HtrA1 involving adhesion or cytoskeleton components would presumably affect their mechanical features.

There is a limited knowledge of the role HtrA1 might play at the cellular level by affecting various RPE functions. Chien et al. identified intracellular proteins that could be potential substrates of HtrA1 by determining cleavage site motifs using mixture-based oriented peptide library screening, and identified tubulins as potential intracellular substrates (Chien et al., 2009a). In this work and in a further study, they validated the idea that HtrA1 is a MAP and observed an inhibition in the migration of the cells (Chien et al., 2009b).

However, these findings do not seem to translate to RPE cells. Indeed, Pei and colleagues reported inhibition of proliferation and migration after HTRA1 knockdown in ARPE19 cells (Pei et al., 2015). Ultimately, the response of RPE could be different to that in SKOV3 (estrogen positive human ovarian carcinoma cell line) and OV202 cells, since they have a completely different origin and role. In addition, primary RPE differ from the ARPE19 cell line, which although revealing some polarization features does not completely display certain key characteristics, such a high TEER (Dunn et al., 1996). In addition, the role of HtrA1 in any of the key functions performed by RPE, such as phagocytosis, has never been explored.

To this end, we sought to recapitulate the increase in *HtrA1* transcriptional levels, which have been associated with disease predisposition and pathogenesis. We found the use of an adenovirus-mediated overexpression more suitable to overcome the primary cells intrinsic variability, resulting in a stable and reproducible approach.

As suggested by Chien et al. we confirmed that overexpression of proteolytic active HtrA1 contributed to tubulin degradation and microtubule loss also in RPE cells. In contrast, we did not observe such a phenotype for the S328A variant, indicating more specifically that the activity of HtrA1 is responsible for tubulin degradation. HtrA1 interactome analysis reinforced the idea that a strong interaction occurs between HtrA1 and several α and β tubulin subunits. We could also confirm the cleavage of tubulin by HtrA1 in non-overexpressing cells using a TAILS proteomics approaches in conjunction with a small molecule inhibitor of HtrA1, indicating that HtrA1 also regulates tubulin in baseline conditions. Interestingly among the other interacting candidates and new to the field, we have found Complement C1q tumor necrosis factor-related protein 5 (C1QTNF5) as specifically interacting with HtrA1. C1QTNF5 is described as a component of basement membranes that may play a role in cell adhesion. More interestingly, mutations in this gene have been associated with late-onset retinal degeneration (Shu et al., 2006). Future studies are necessary to elucidate the implications of other HtrA1 cleaved products such as C1QTNF5 and other interacting candidates.

Microtubules are important in many cellular functions, including cell division, shape integration, force transduction and vesicle transport. In case of differentiated RPE, where no divisions are occurring and little migration is expected, the most probable functions that could be affected by tubulin dysfunction would be those concerning the mechanical structure maintenance(Schaedel et al., 2015) and specific processes from RPE where an active participation of the cytoskeleton is crucial, such as the phagocytosis of the OS (Ruggiero and Finnemann, 2014).

Thus, we assessed the phagocytic function and observed that cells overexpressing HtrA1 showed diminished phagocytic activity, which could be further confirmed with OS. In the light of these results, it would be reasonable to expect the photoreceptors to suffer from the accumulation of OS, which would not be properly engulfed, eventually compromising the survival of these cells, a typical pathological phenotype of AMD. Indeed, impairment of phagocytosis and renewal of POS induce retina degeneration resembling AMD like phenotypes, as reported in knockout mice such as ATP-binding cassette transporter 4 (ABCA4) or alphavbeta5 integrin (Nandrot et al., 2004, Radu et al., 2004).

In the retina, microvilli of the RPE apical membrane interdigitate with the OS of the photoreceptors (Marmorstein et al., 1998). Absence of microvilli was revealed by apical imaging of RPE cells overexpressing active HtrA1 but not when the S328A was overexpressed. It is tempting to speculate that this apical dysfunction could be the reason behind the diminished phagocytic activity of the diseased cells. This speculation is consistent with the observation that cells having the longest apical processes were the first among the population to uptake the outer segment particles. How the protease could be affecting the apical processes is still a question to be answered, since the microvilli are composed of actin filaments. Further investigation would need to be done to explore the relationship between the actin compartment of the cytoskeleton and the rearrangements occurring when tubulin is degraded.

Microtubules have a significant contribution to cell stiffness in cells and hence, its disruption could cause a decrease in cell stiffness (Stamenovic, 2005). In line with this, we demonstrated that RPE cells overexpressing HtrA1 exhibited a significant decrease in tubulin content which translated into a modification in the cellular stiffness (Fig. 4A). As previously reported, rearrangements in the microtubule component of the cytoskeleton and melanosomes have been associated with cell softening in RPE (Sarna et al., 2016). Notably, we also observed that RPE cells overexpressing HtrA1 appeared to be less pigmented than the S328A ones (data not shown). In line with that, a recent study reported a reduction in the RPE melanosomes in AMD patients suggesting that age-related changes of RPE melanosomes may correlate with the development of this disease (Booij et al., 2010).

Additional evidence comes from observations made at the cellular junctions. For example, we observed clear deposition of the HtrA1 at the adherens junctions and not at the tight junctions as indicated by the prominent staining below Claudin19 and co-localization with *E*-cadherin. Taken together; major decrease in RPE stiffness associated with increased tubulin degradation and co-localization of HtrA1 with E-cadherin at the adherens junctions provides a plausible explanation for the loss of adhesion in the RPE cells but not the lack of barrier function. Indeed, computational models of AMD, suggested that defects in the adhesion dominate the neovascularization initiation and progression (Shirinifard et al., 2012). The physical and mechanical barrier that in physiological conditions confers resistance to the vessels would, in a pathological milieu, allow for the choroid to trespass to the neural retina (Dugel and Zimmer, 2016). Future studies are necessary to identify the exact interactors at the cell-cell junction level and to further investigate the mechano-functional relationship between HtrA1 and the RPE adhesion loss.

Chien et al., suggest that at low concentrations, the protease promotes higher steady-state levels of tubulin polymerization *in vitro*. At high concentrations, on the other hand, HtrA1 would have the opposite effect and reduce tubulin polymerization. However, these data were produced by polymerizing the MT at 25 °C, and the behavior of HtrA1 would not necessarily be the same at 37 °C. In addition, Chien et al. did not investigate the role of the S328A variant or the role of a full-length HtrA1.

Our results, obtained physiological conditions, show a very clear difference between HtrA1 and S328A. HtrA1 disrupted the formation of MT, whilst the mutant S328A conferred stability to the polymers and enhanced microtubule levels to above those of the control tubulin. MT polymerized in the presence of the active HtrA1 had a lower growth rate and the time needed for enucleation was substantially longer. This delay could possibly be explained by the concomitant degradation occurring as the MT are forming. It is also remarkable, that we could observe an amplification of the described patterns when the experiments were performed with taxol, where it was observed that the stabilizing agent was additive to the effects of S328A but could not rescue the activity of HtrA1.

Since we found that more tubulin polymer formed in the S328A condition, we hypothesized that two distinct events could be occurring. Either more MTs were being formed or the MTs formed in presence of the S328A HtrA1 were longer. In this regard, the experiments where we polymerized the MT first and then added the protease afterwards (Fig. 7A and B) would be providing an answer. In the presence of the S328A protease, the MTs were aggregating becoming longer than the control ones. It is reasonable to think, that MTs polymerized in the presence of S328A also became longer and this lead to the higher amount quantified after 120 min of polymerization.

Taken together, it seems that the S328A variant has a stabilizing effect that could be observed both in the polymerization of new MT as well as in those already polymerized. The active protease HtrA1 seems to behave differently. When HtrA1 is incubated with preformed MT, it appears to first aggregate and slightly stabilize MTs and then degrade them (as seen after 24 h). During polymerization, the stabilization by HtrA1 does not occur and only degradation takes place. Therefore, there could be a double role: one of stabilization and one of degradation. The stabilizing effect seems to be an intrinsic function of the HtrA1 protein unrelated to its proteolytic activity when the MTs are already formed. In contrast, the degradation role seems to be specific and mediated by the enzymatic active HtrA1 on either growing or already formed MT, provided the protease is present in high concentrations.

This dualistic nature of HtrA1 observed *in vitro* would clarify why we are able to find MT in the S328A overexpressing cells, which fade in the HtrA1 condition. It could explain also the observation of higher frequency of multiciliation events in the S328A variant. Indeed, for this latest process polymerization of tubulin *de novo* is necessary and RPE cells overexpressing HtrA1 would not be able to achieve this step, clarifying the lack of mutliciliated events in such condition.

In conclusion, we have established a polarized RPE cell culture model that enables exploration of the functional consequences of pathological HtrA1 increase observed in human retinas form AMD patients. Overexpression of HtrA1 has revealed a diminished content of tubulin along with a prominent decrease in cell stiffness and dysfunction in the apical processes of the RPE cells. This disease phenotype translates into altered mechanical stability of the retinal layer and impaired phagocytosis activity. We have elucidated the molecular mechanisms by which the HtrA1 enzymatic activity affects either the intracellular degradation or polymerization of tubulin and consequently alters the RPE mechanics. Taken together our cell culture model provides an essential platform to investigate functional role of HtrA1 in RPE cells with significant implications for developing therapeutic interventions targeting HtrA1 at the intracellular level.

## Disclosure of potential conflicts of interest

E.M., H.M., A.C.C., JC.H., T.KT., R.S., L.B., N.F., Z.*E.G.*, F.D., C.S., G.G., A.F., B.H., S.G., J.SP., F.G., B.B., T.D., S.F., R.I., are (or were) Roche employees (at the time when the study was conducted). Roche holds patent applications for small molecules inhibitors for the HtrA1 protein.

Work of MP and PO was supported by the Swiss National Science Foundation Nanotera Project awarded to the PATLiSci II Consortium.

The University of Basel has filed patents related to the AFM technology based on the inventions of M.P., P.O. and R.Y.H.L.

## Author Contributions

E.M., P.O., C.T., T.B., L.S., A.C.C., C.C., N.F., Z.*E.G.*, F.D., C.S., G.G., A.E., S.G., F.G., T.K., R.S., L.B. J.S.P., performed/analyzed experiments, and B.B., H.M., C.F., T.D., S.H., O.S., V.E., S.F., M.P., and R.I. designed experiments. E.M., M.P., O.S., T.D., V.E., H.M., and R.I. interpreted the data. E.M., O.S., T.D., M.P., and R.I. wrote the manuscript.

## Appendix A Supplementary data

Supplementary material Image 1
