# Supplementary material for: HtrA1 Mediated Intracellular Effects on Tubulin Using a Polarized RPE Disease Model
Source: eBioMedicine. 2017 Dec 13;27:258–74. doi: 10.1016/j.ebiom.2017.12.011 (PMC5828370; doi:10.1016/j.ebiom.2017.12.011)
Supplement: Supplementary file 1 — Supplementary material [file mmc1.docx]

**Figure S1 – related to figure 1**

**
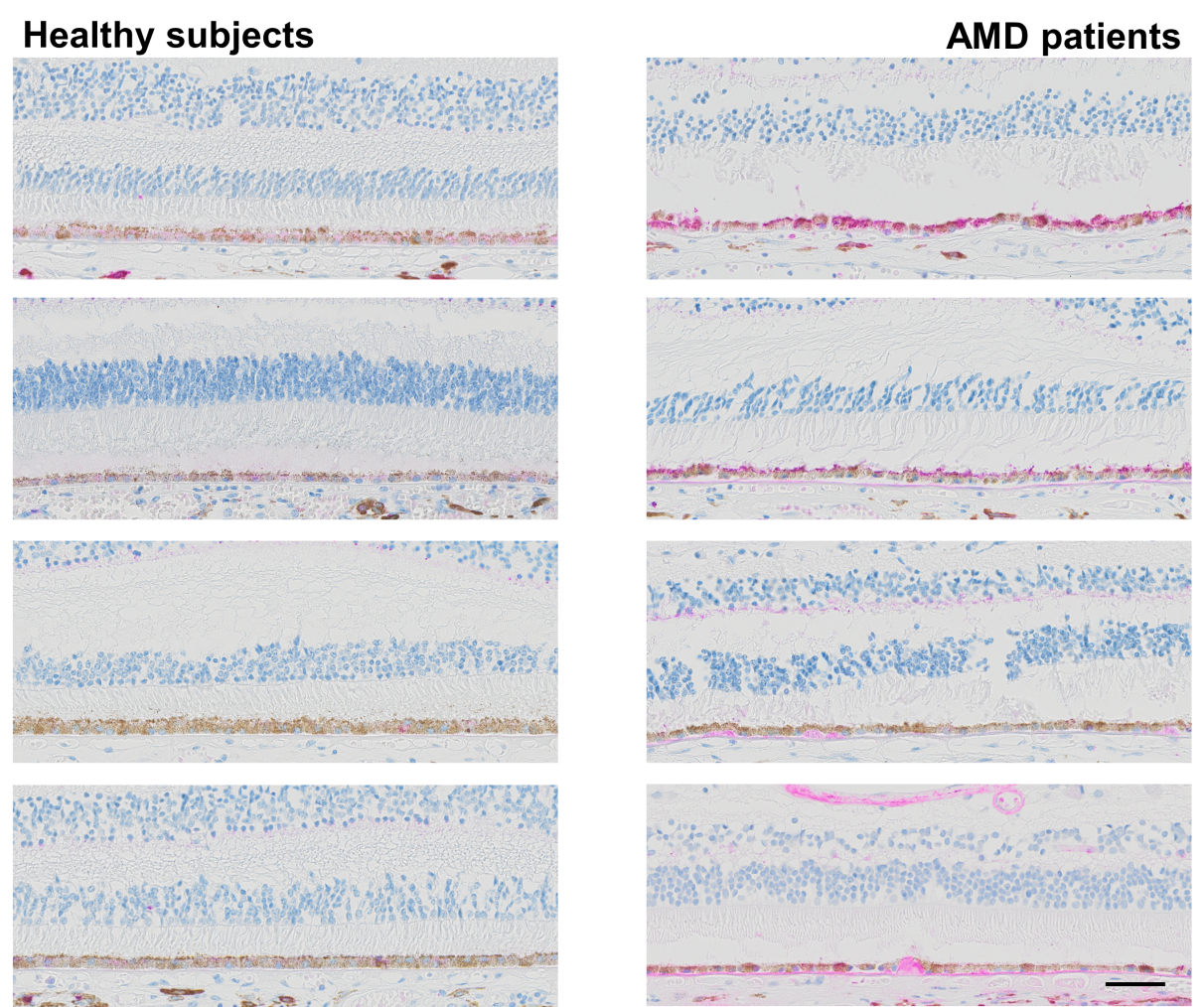
a**

**b**

**
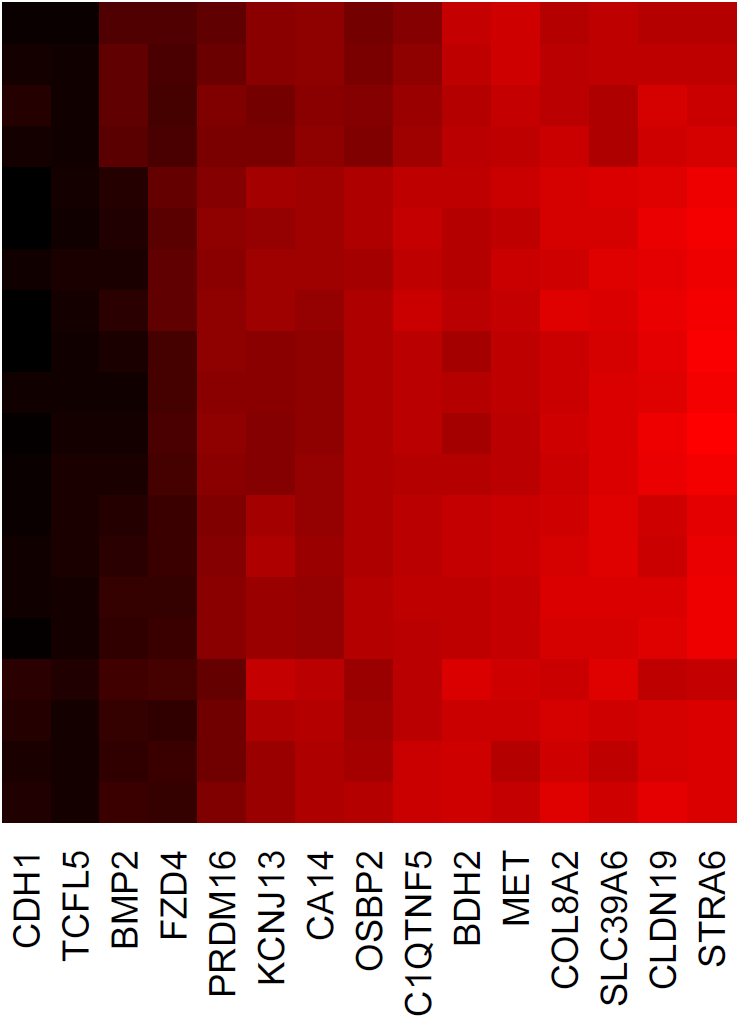
**
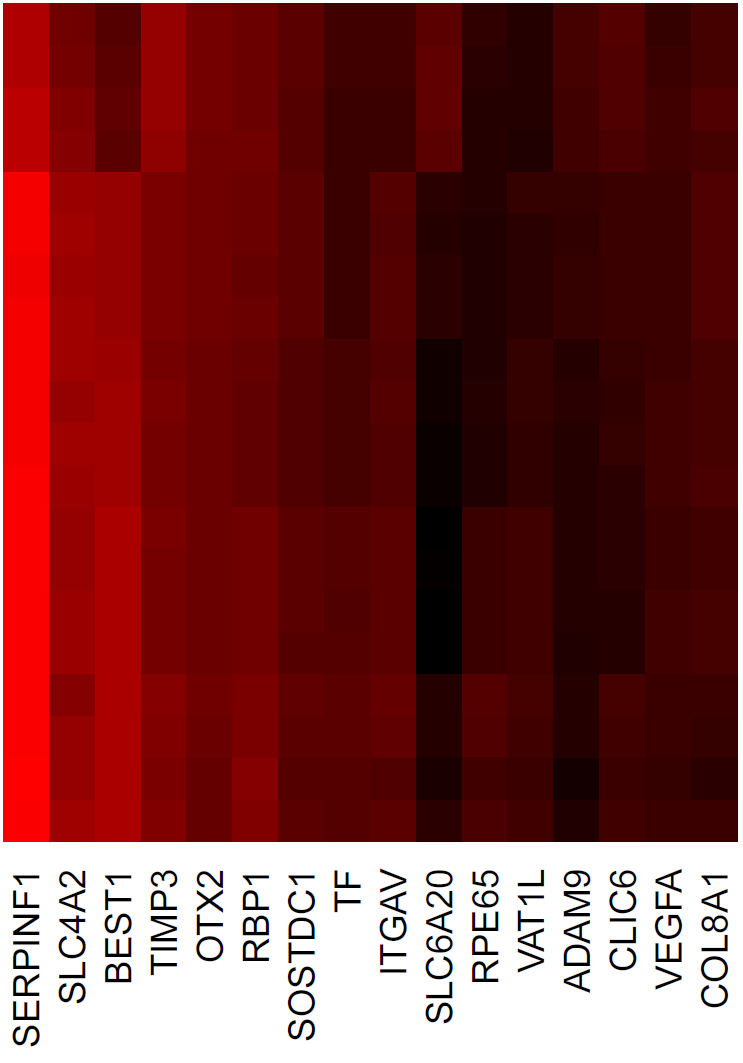
**Genes in consistently high expression Genes in consistently low expression**

**
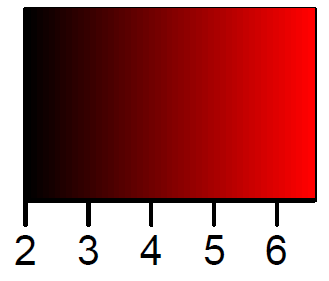

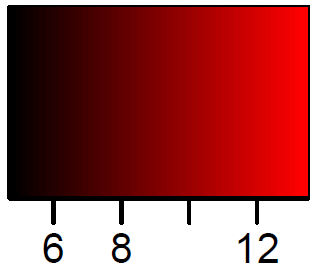
**

**
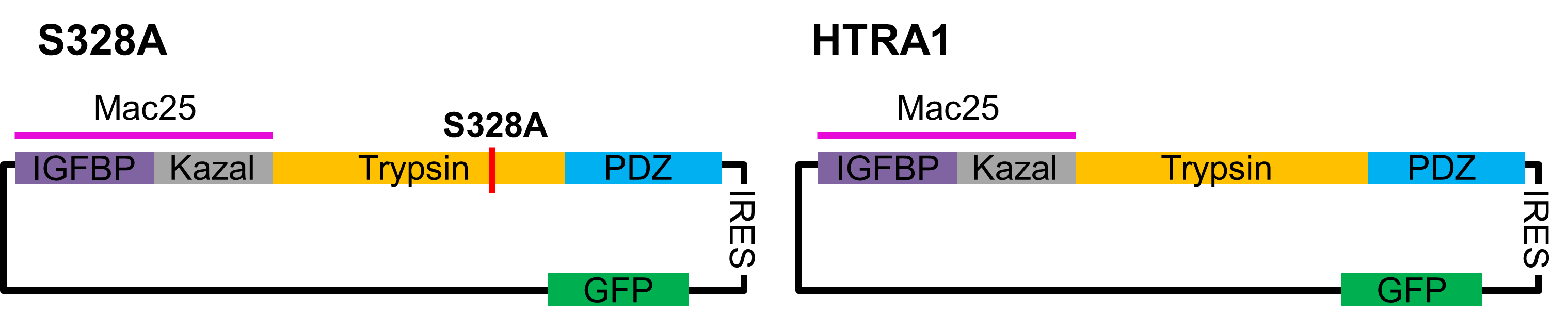
c**

**
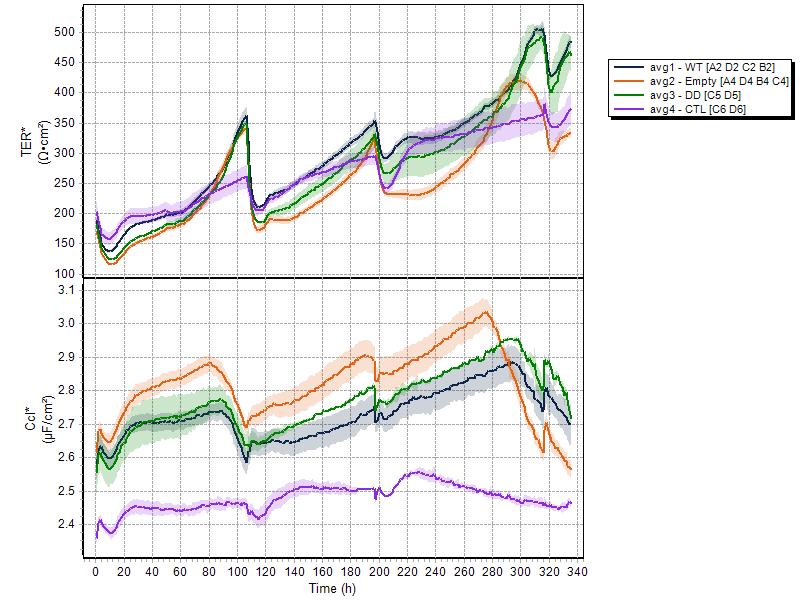
**

HtrA1 S328A Empty Vector Control cells

**S328A**

**HtrA1**

**d**

**
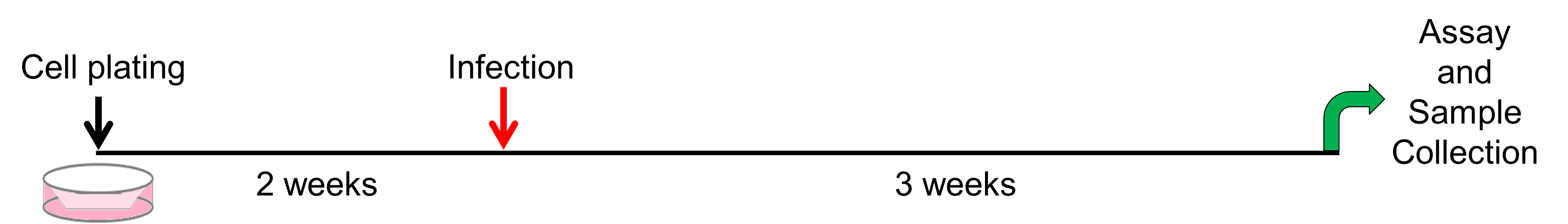
**

**e f**

**
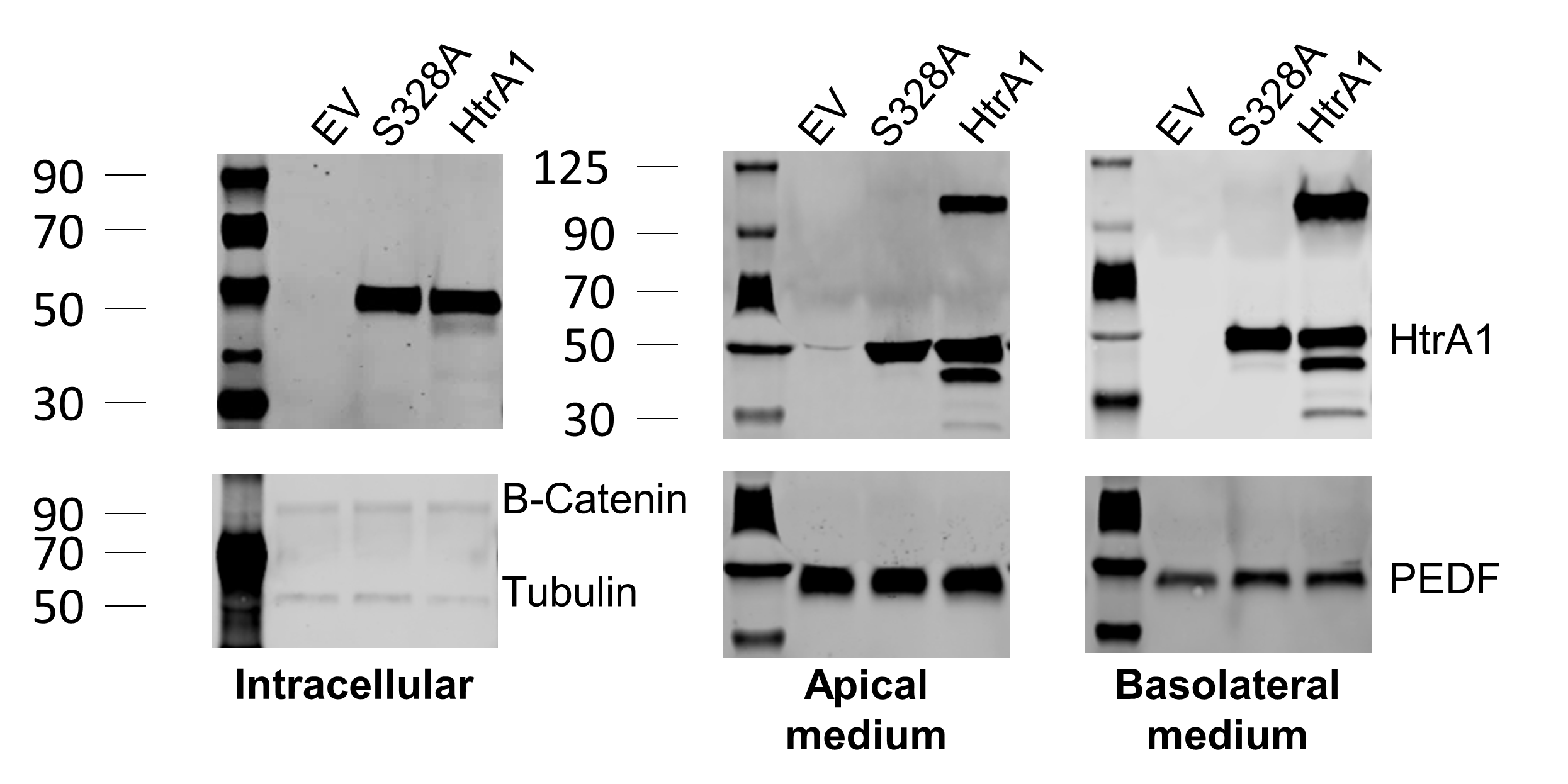
**


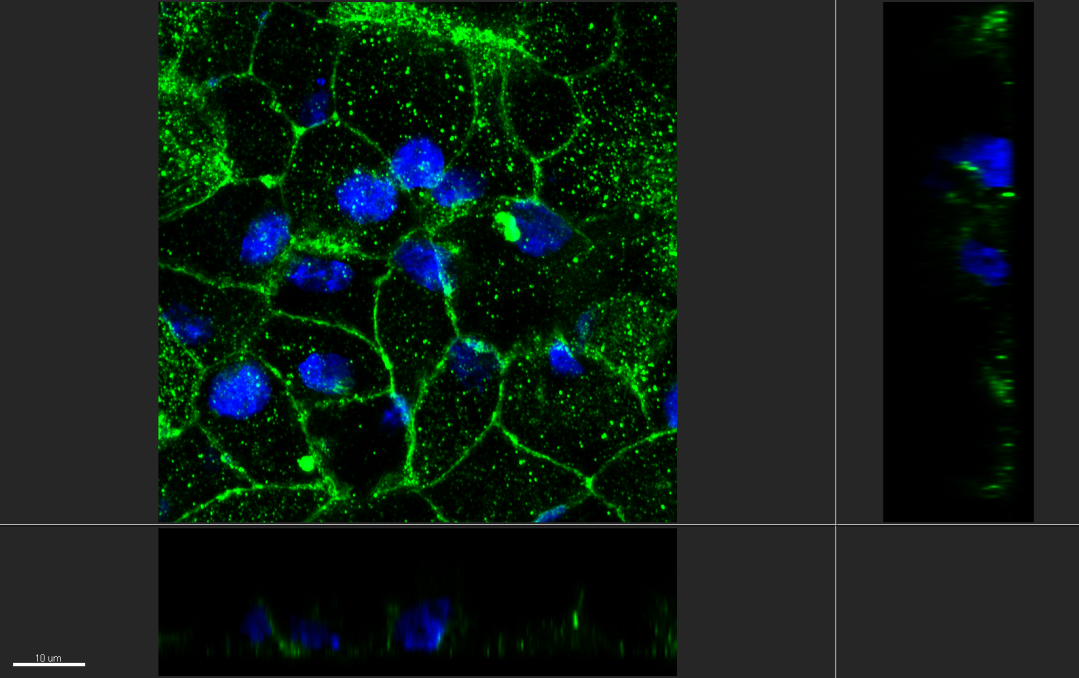

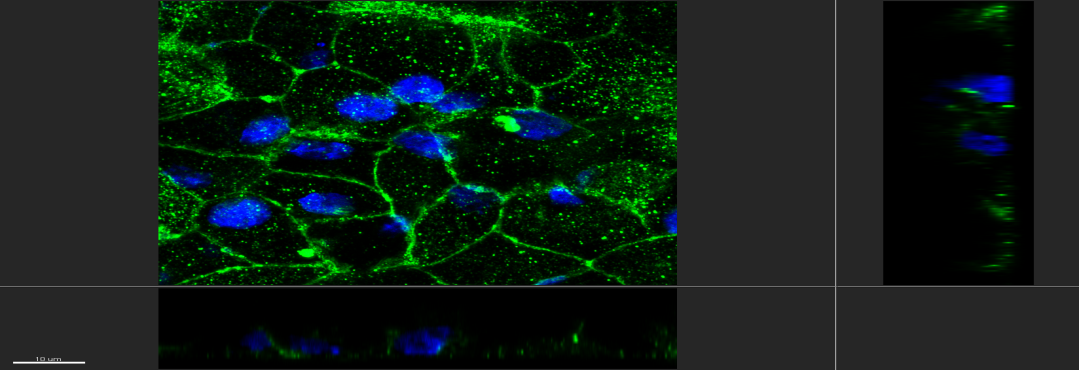

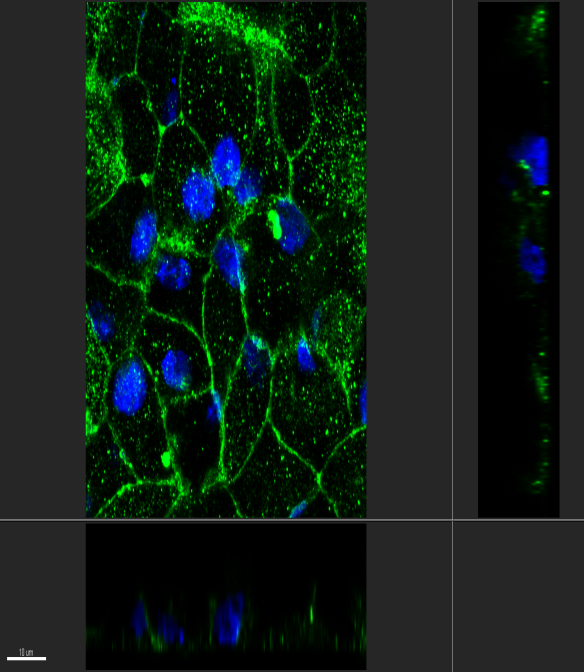


**Na^+^K^+^ ATPase / Nuclei**

**Figure S2 – related to figure 2**

**a**
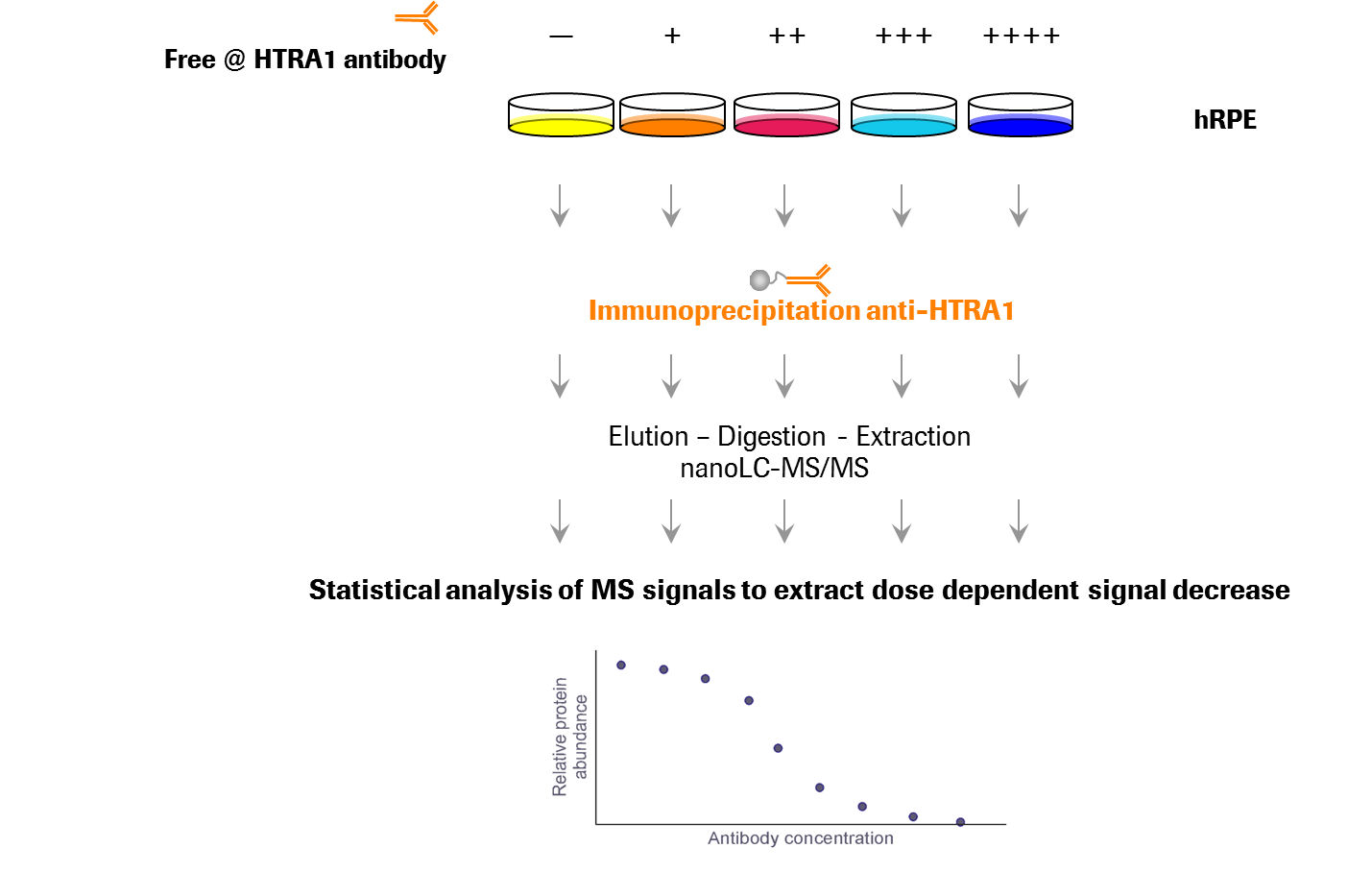


**b**

**Compound RI2404:** N-[(2S)-3-(3-chlorophenyl)-1-[[(1S)-2-[[(3S)-5,5-difluoro-2-methyl-4,6-dioxo-6-(2,2,2-trifluoroethylamino)hexan-3-yl]amino]-1-(4-methoxyphenyl)-2-oxoethyl]amino]-1-oxopropan-2-yl]pyridine-2-carboxamide)

**IC50 (HTRA1) = 0.0085 uM (average value, measured 3 times)**

**IC50 (CatS) > 100 uM**

**IC50 (CatG) > 40 uM**

**IC50 (Elastase) = 0.42 uM**


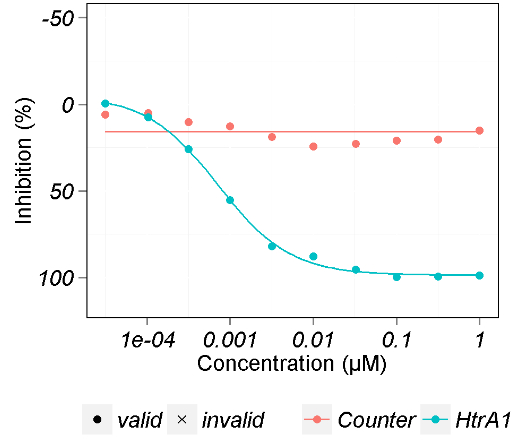


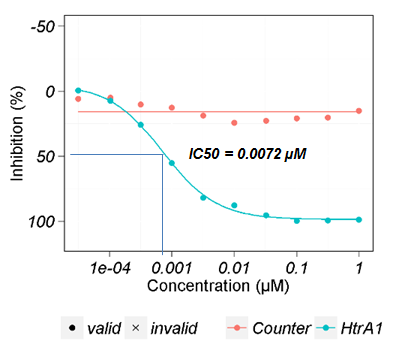


**Compound EM3770:** N-[(2S)-3-(3-cyanophenyl)-1-[[(1S)-2-[[(3S)-5,5-difluoro-2-methyl-4,6-dioxo-6-(2,2,2-trifluoroethylamino)hexan-3-yl]amino]-1-(4-methoxyphenyl)-2-oxoethyl]amino]-1-oxopropan-2-yl]pyridine-2-carboxamide

**IC50 (HTRA1) = 0.0089 uM (average value, measured 4 times)**

**IC50 (CatS) > 100 uM**

**IC50 (CatG) > 100 uM**

**IC50 (Elastase) = 0.39 uM**


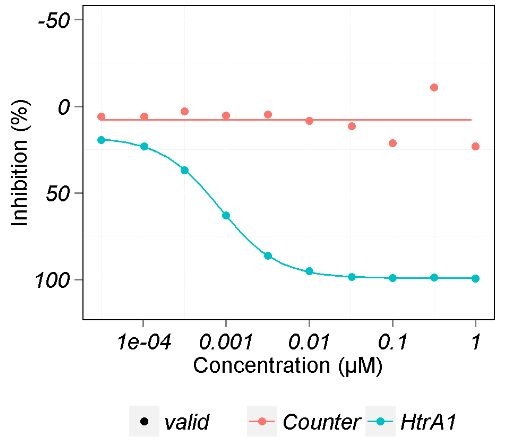


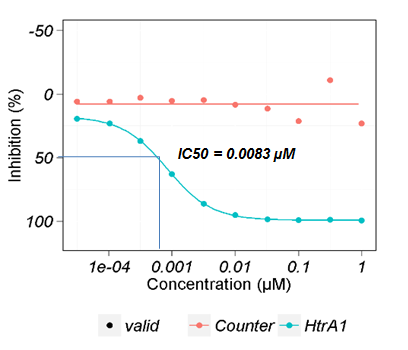


**c**

**
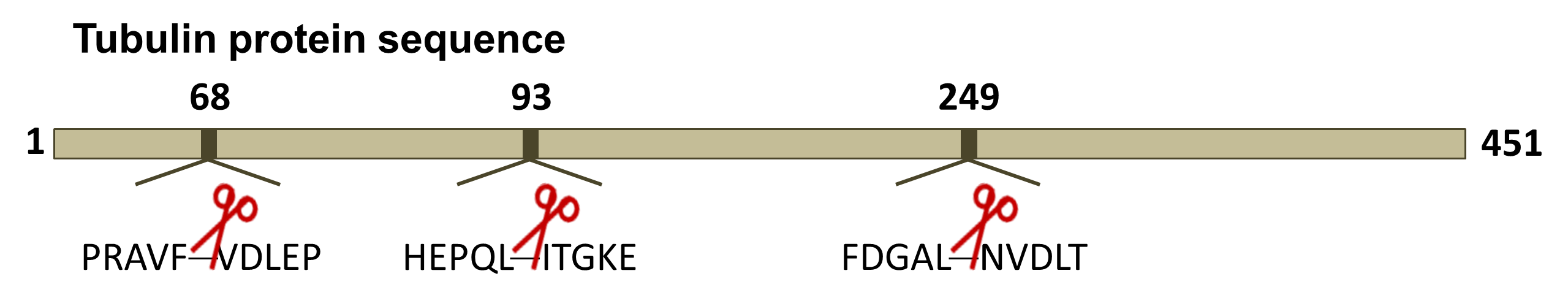
**

**Figure S3 – related to figure 3**

**a b**


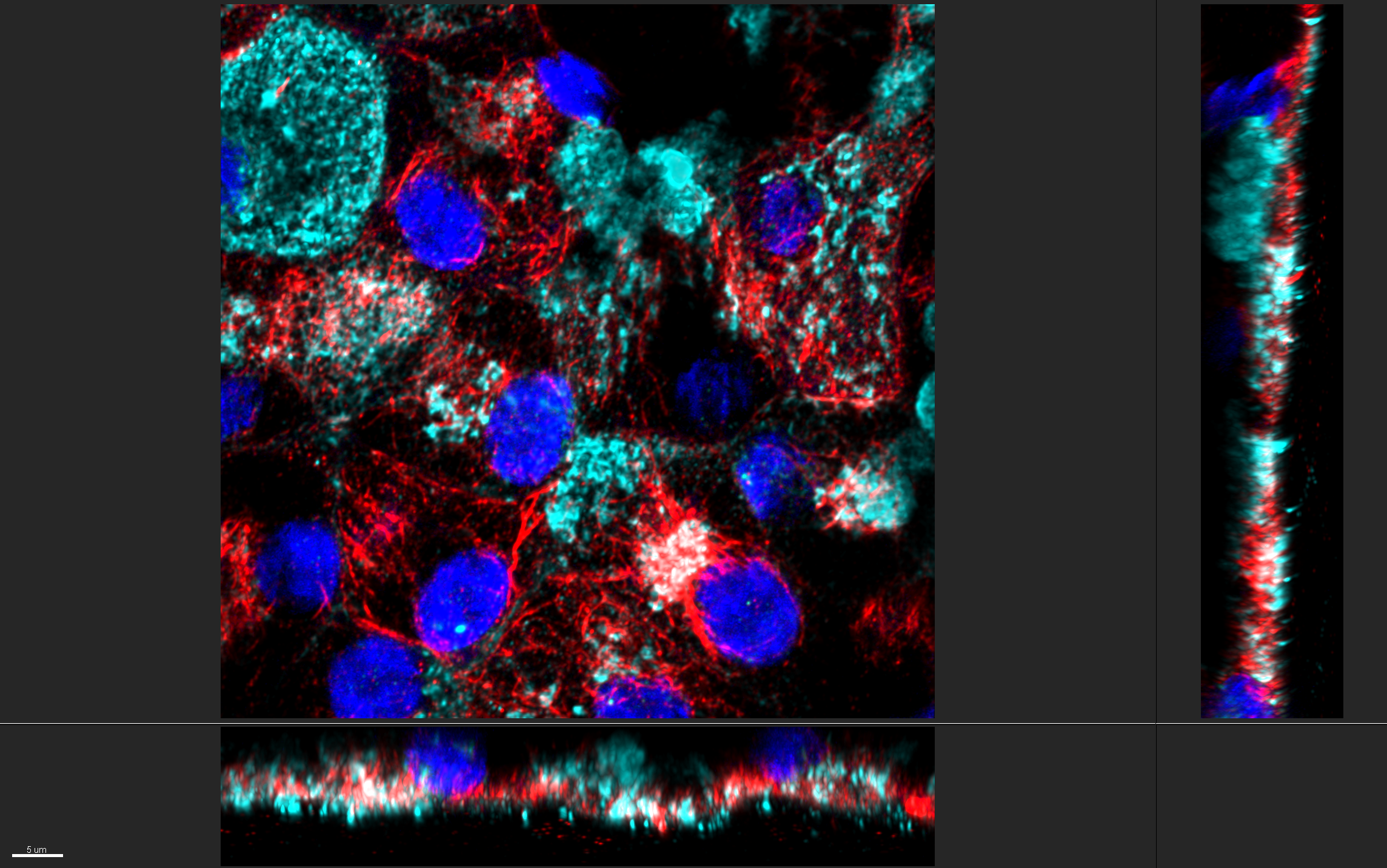

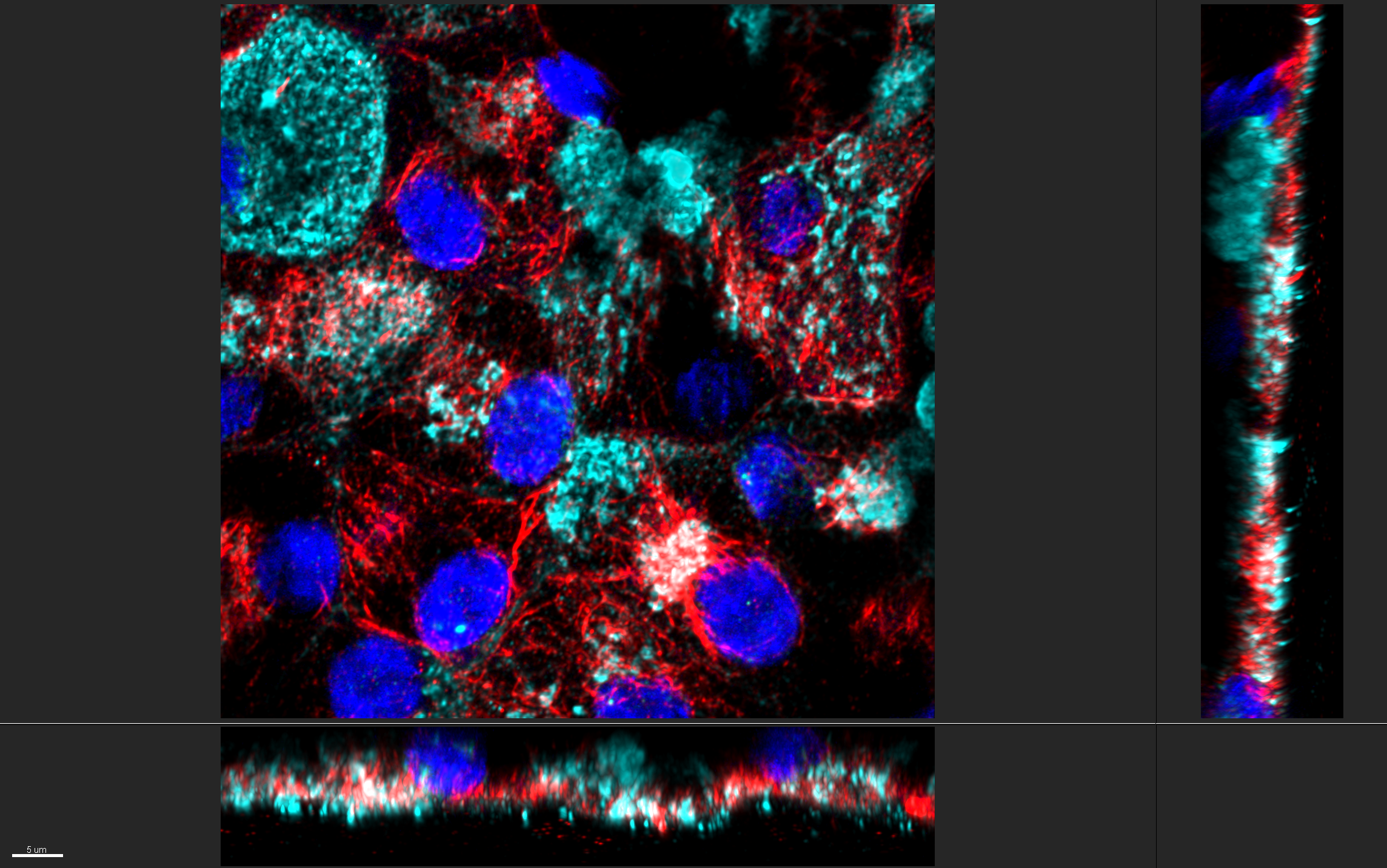


**HtrA1 / α-acetylated tubulin / Nuclei**

**HtrA1 / α-acetylated tubulin**


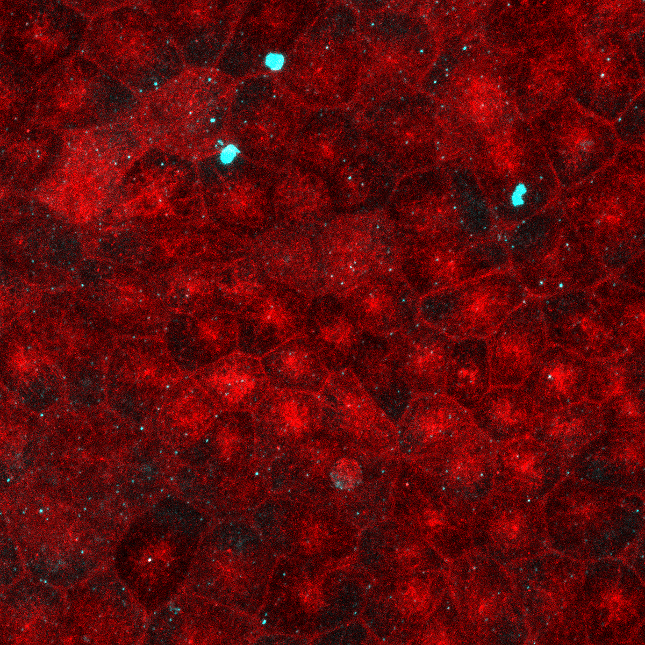


**c**


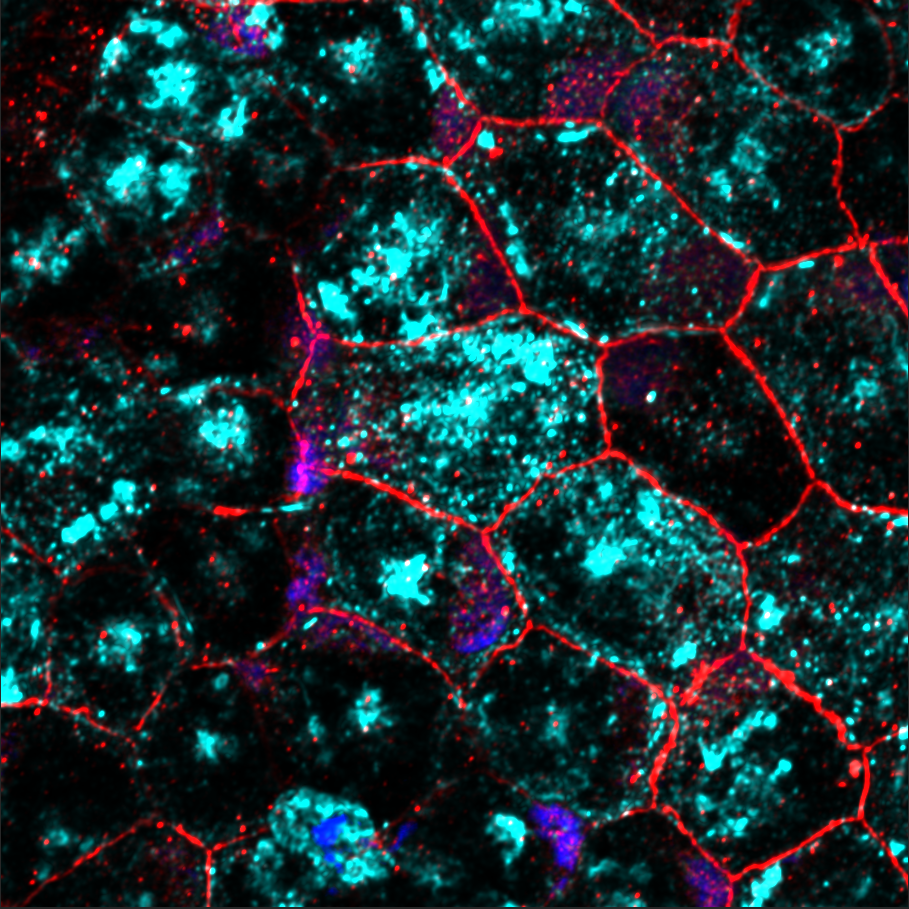

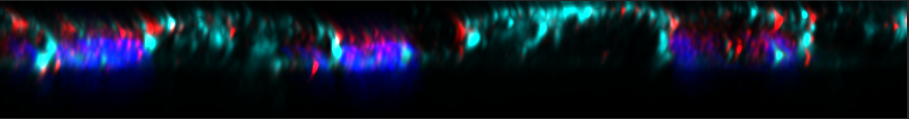

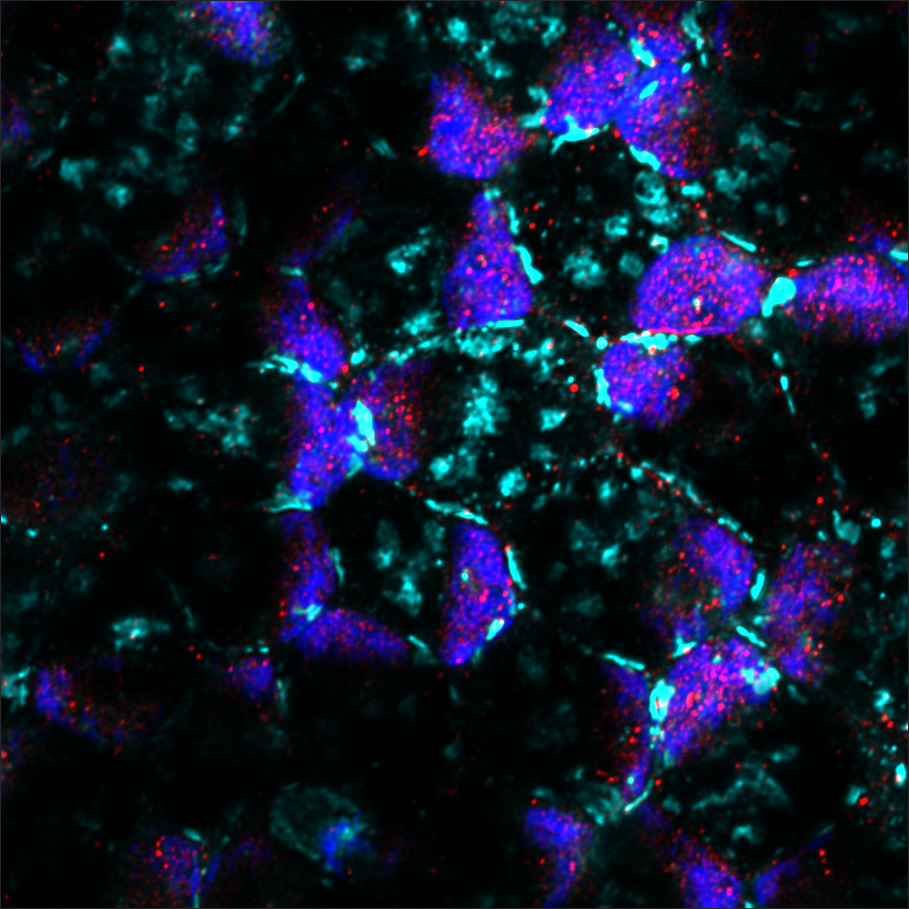


**HtrA1 / Claudin 19 / Nuclei**

**Basal Apical**

**
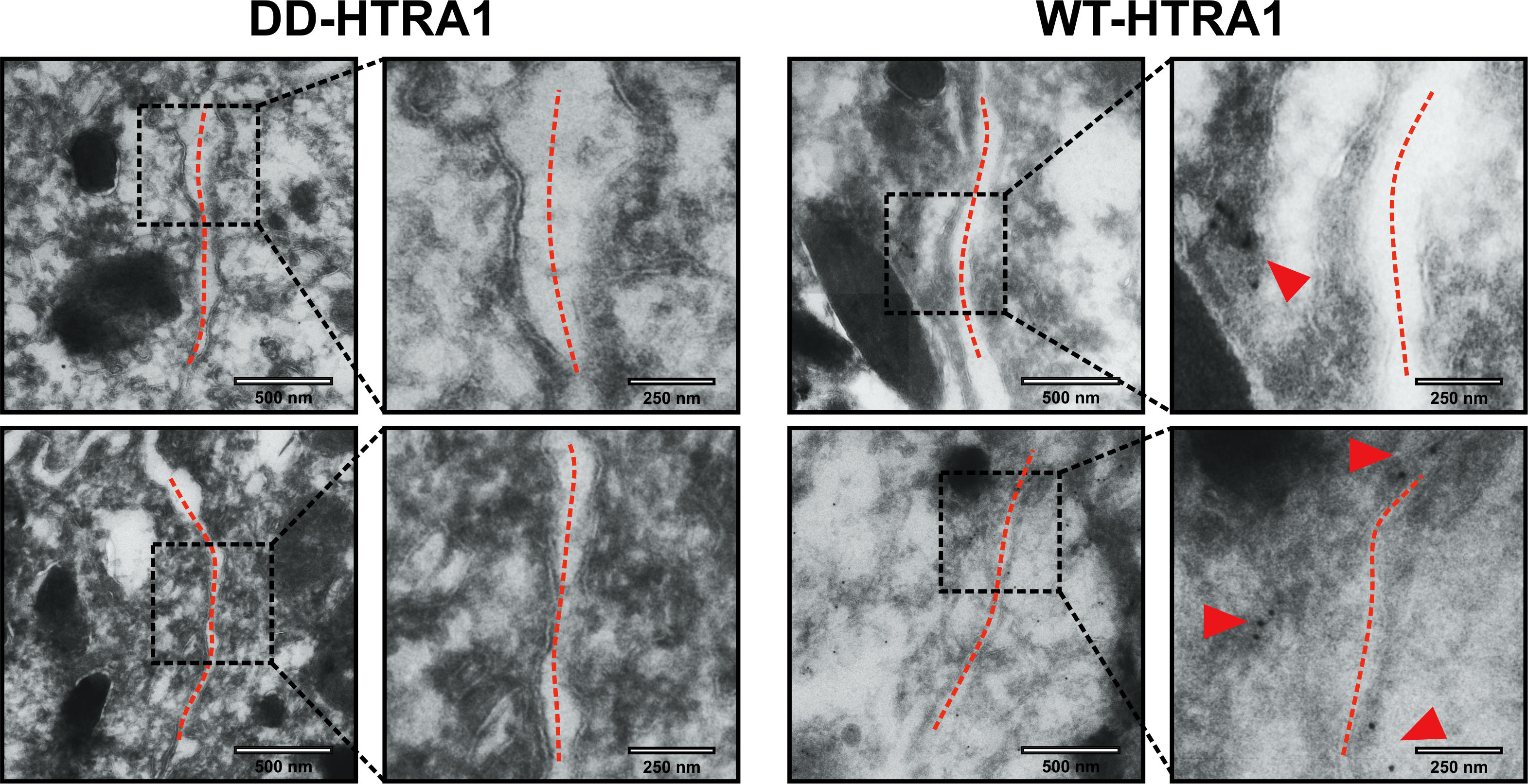
d**

**Figure S4 – related to figure 5**

**a**


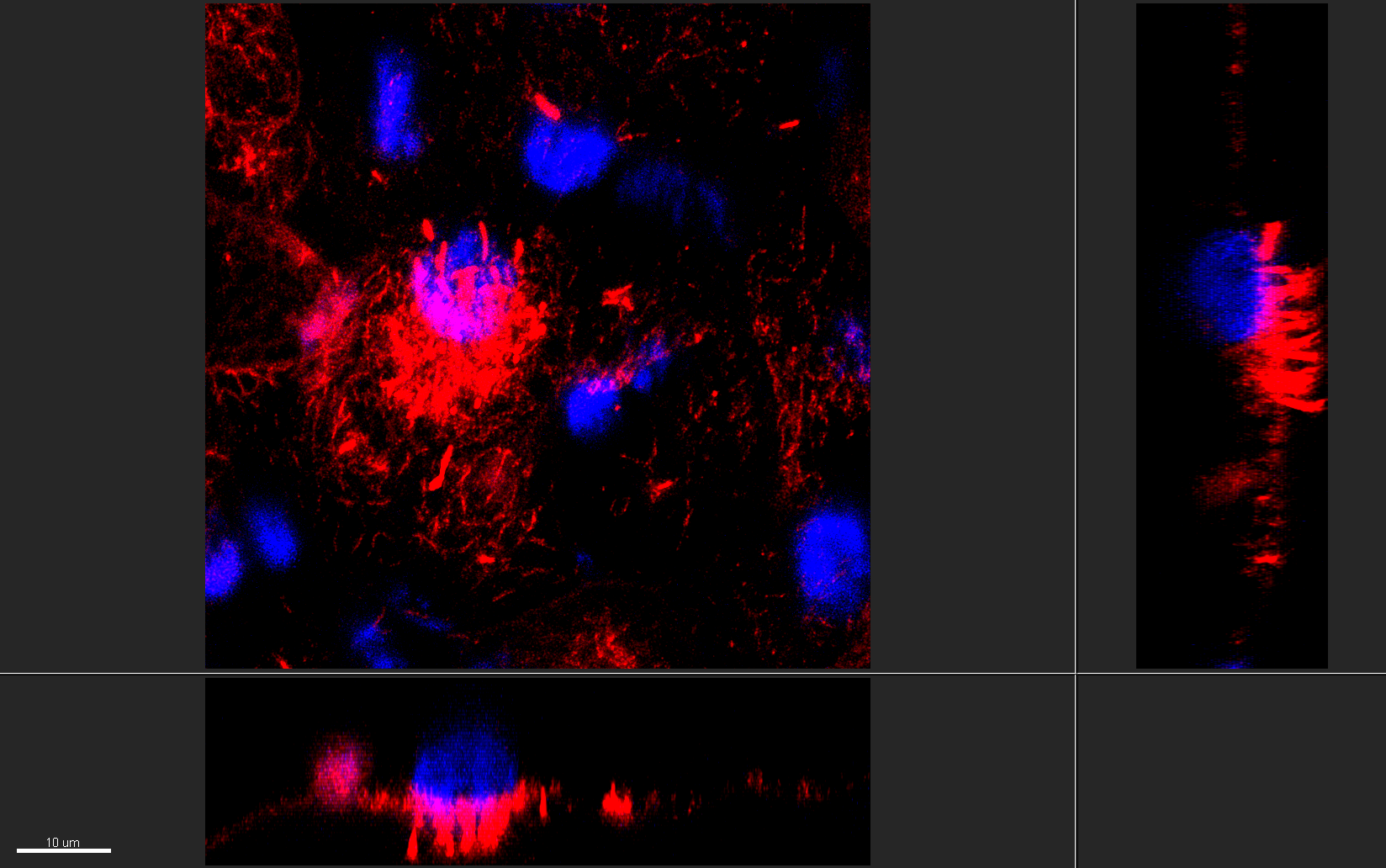

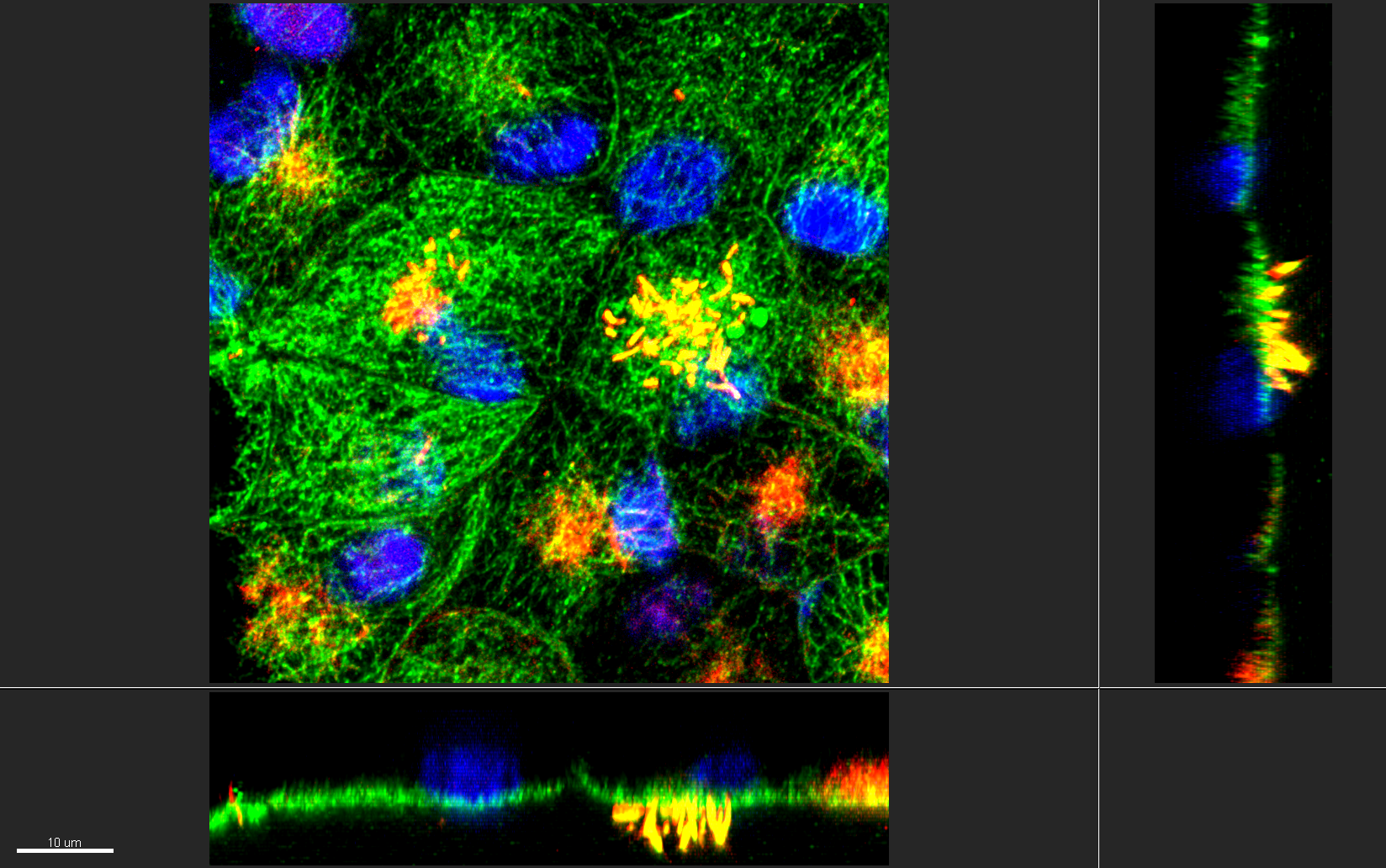

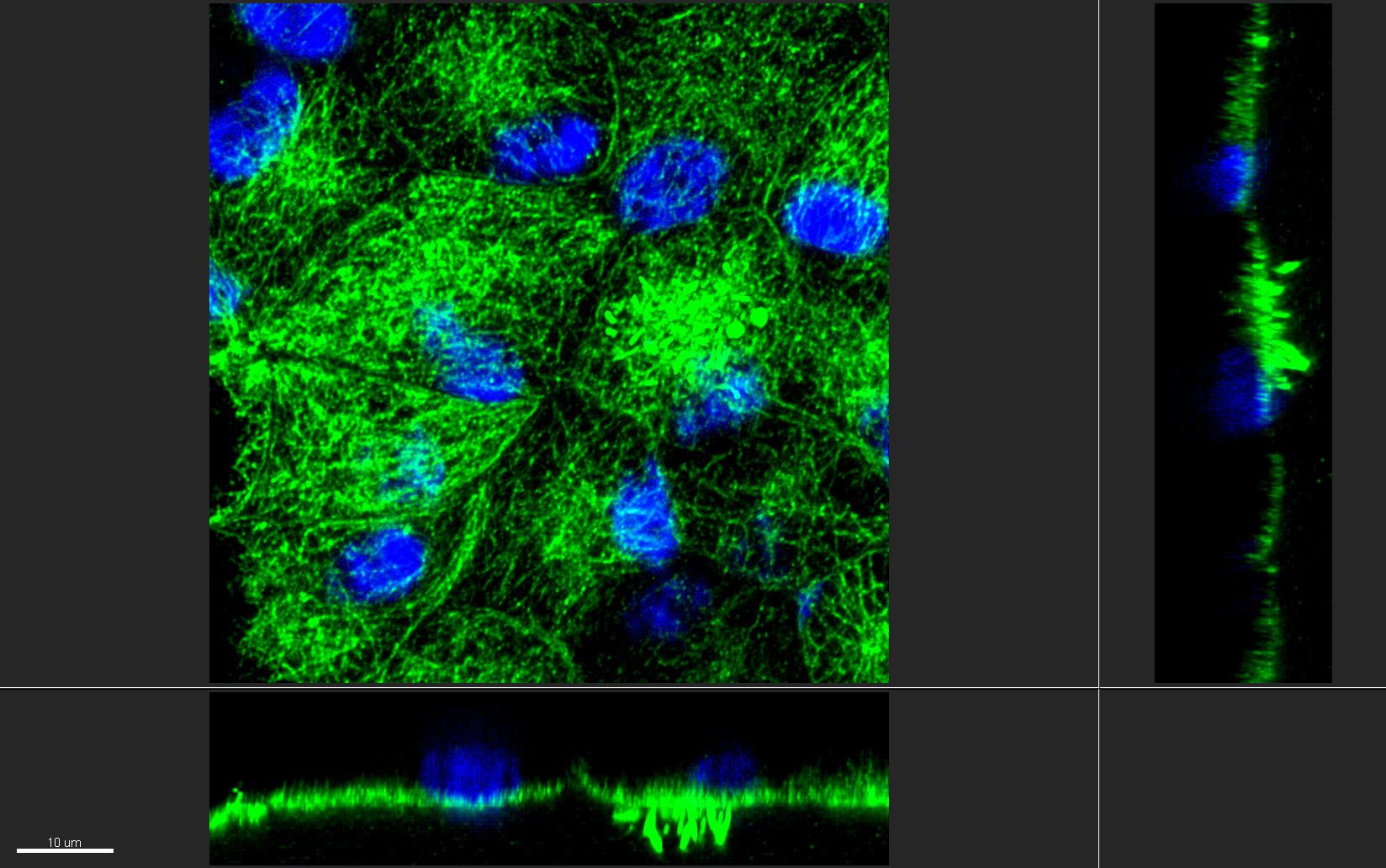

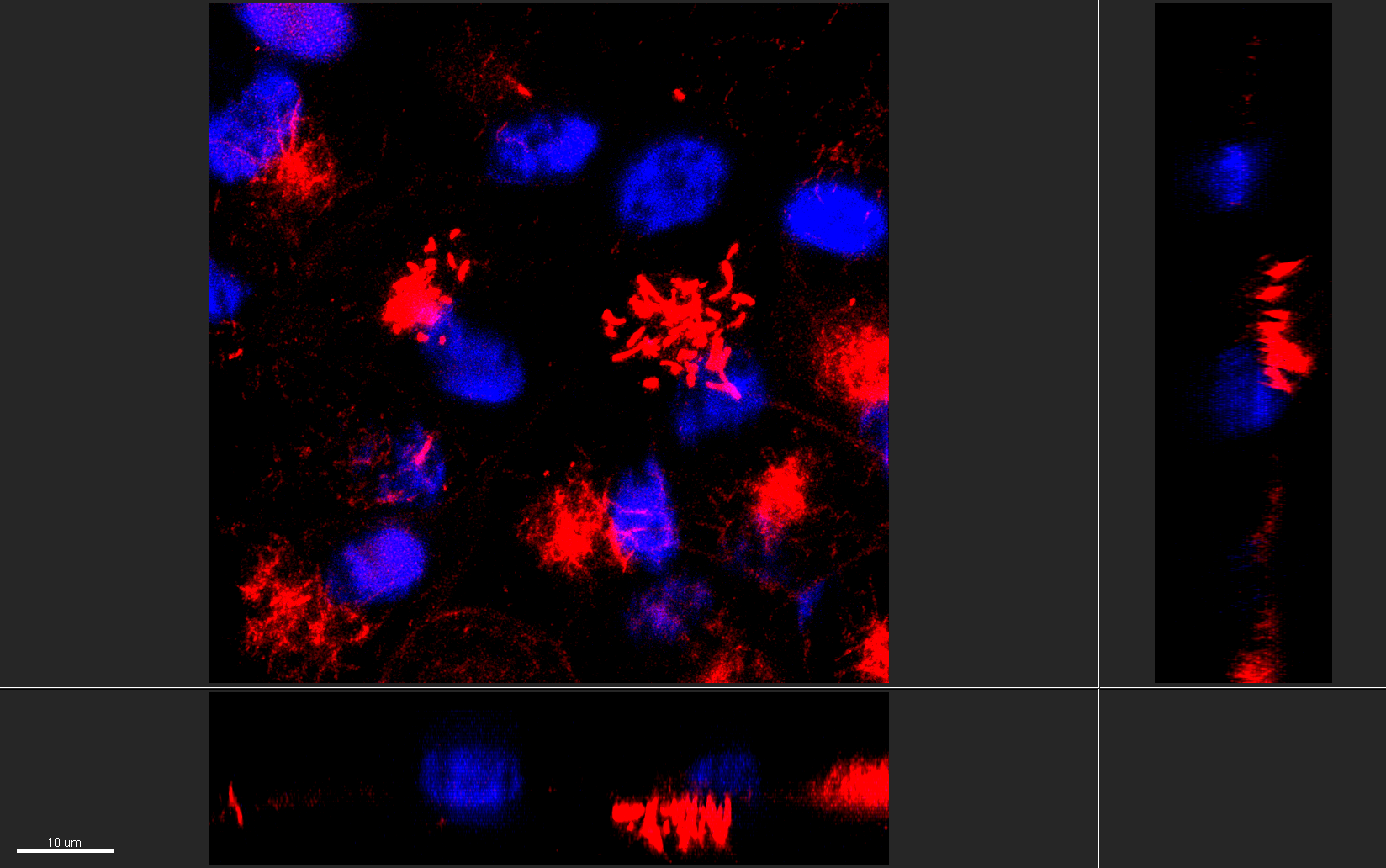

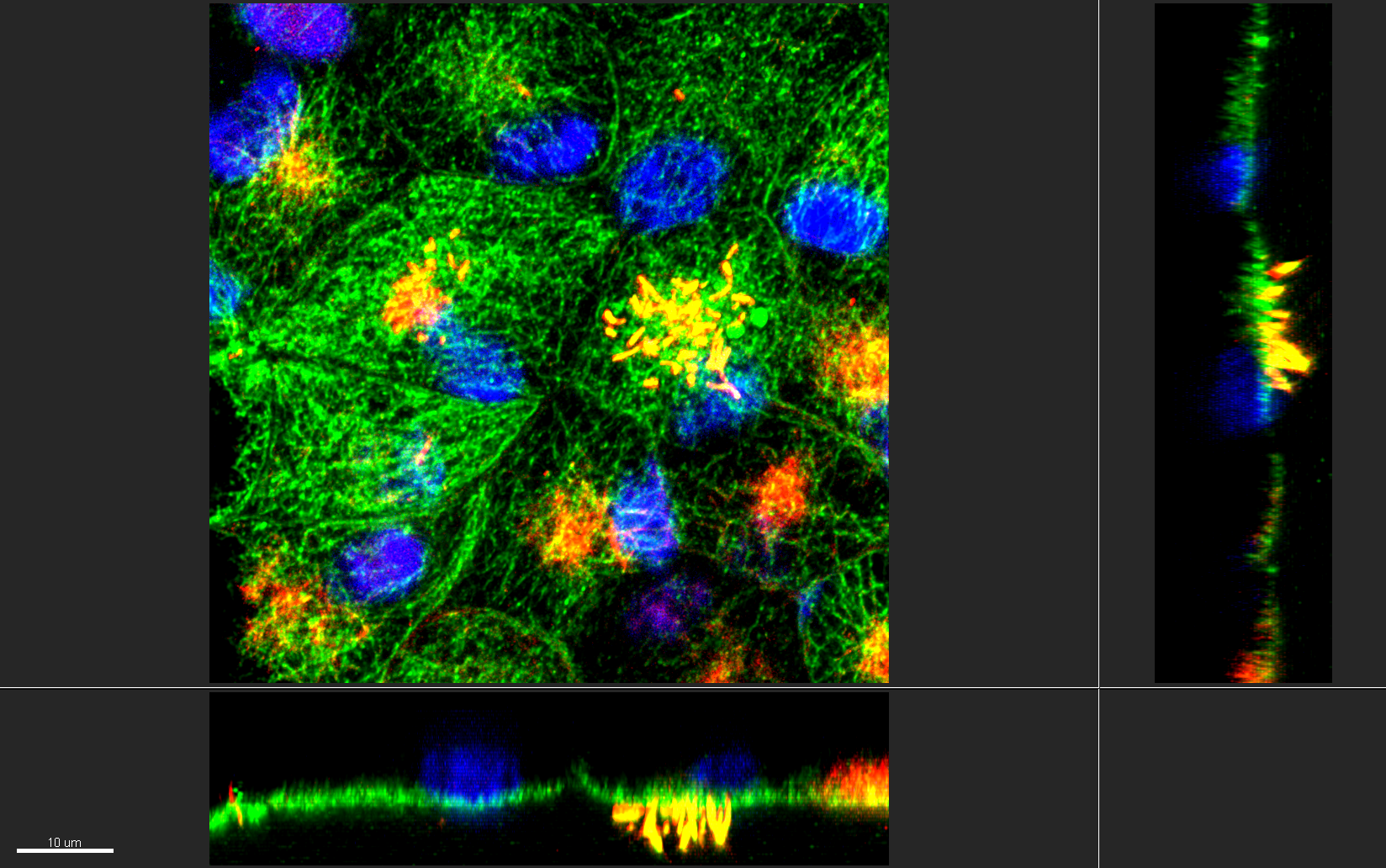

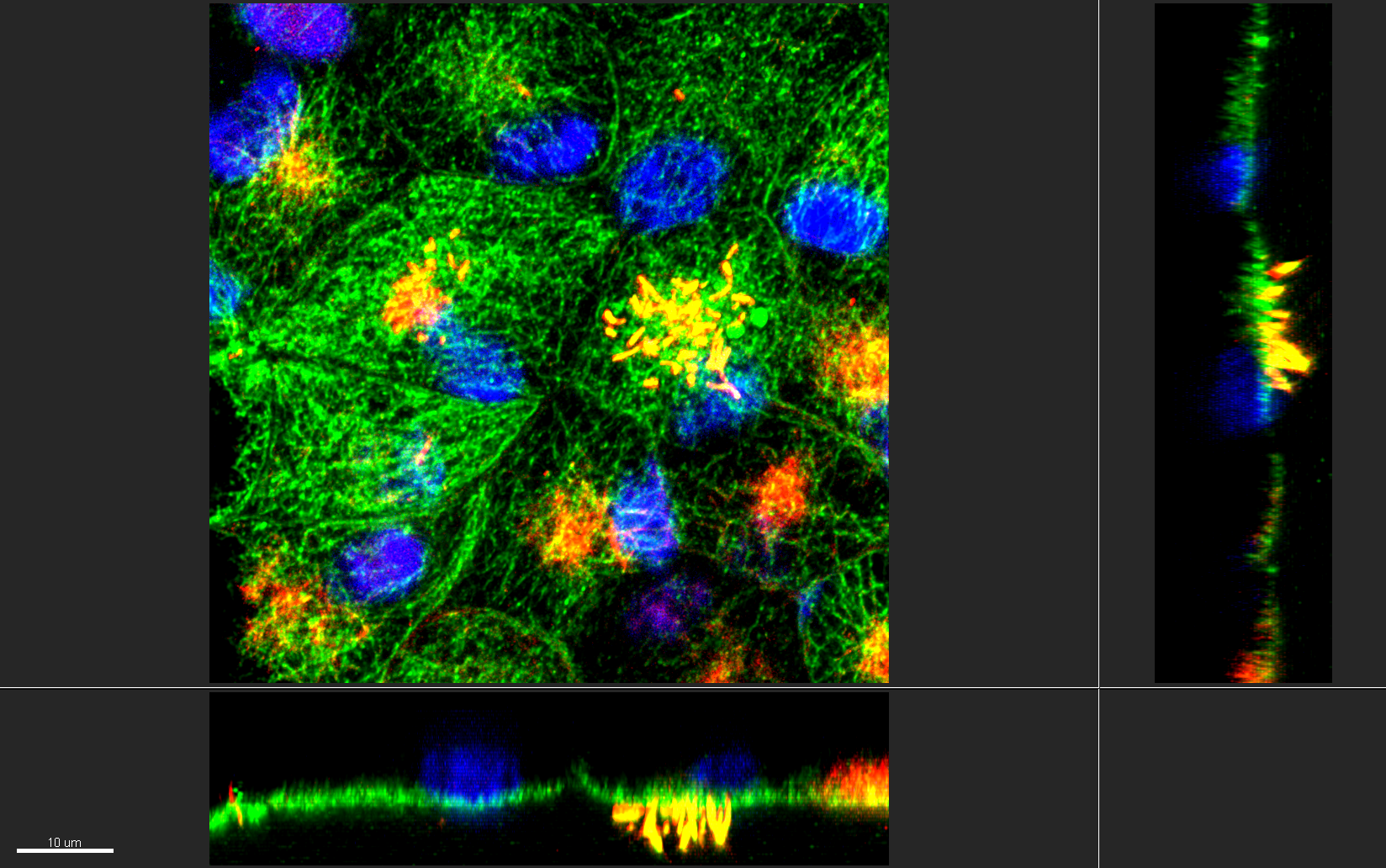

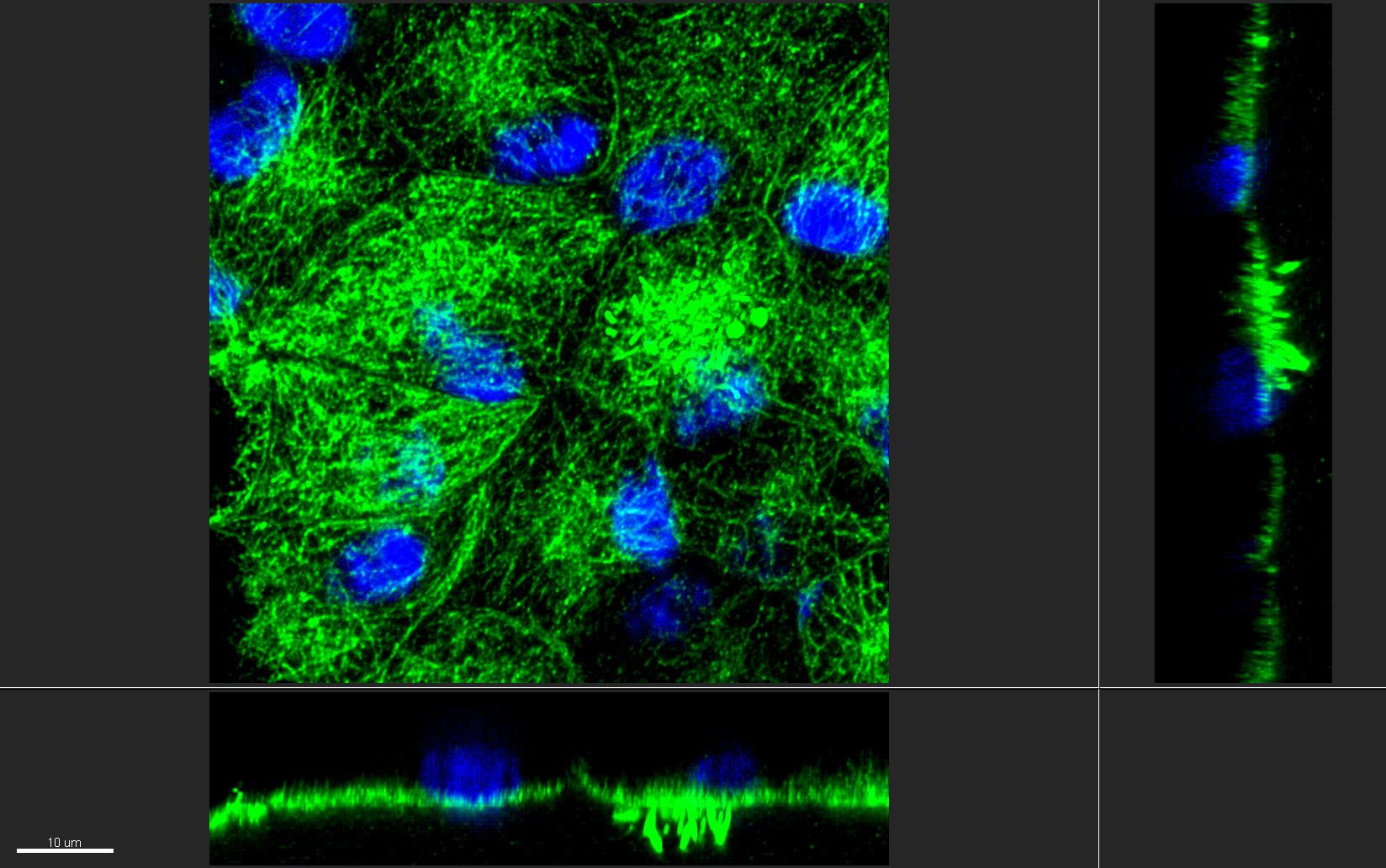

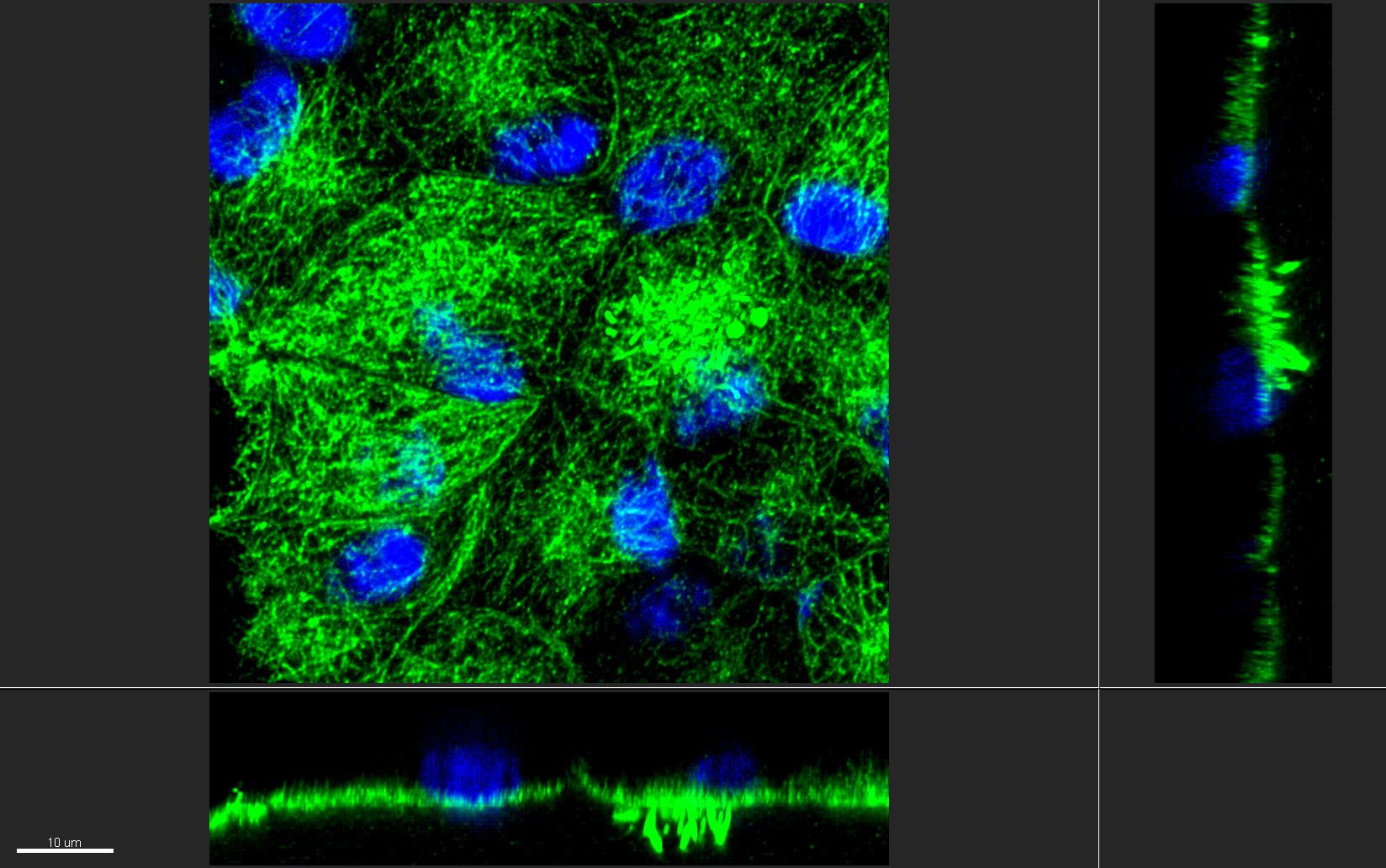

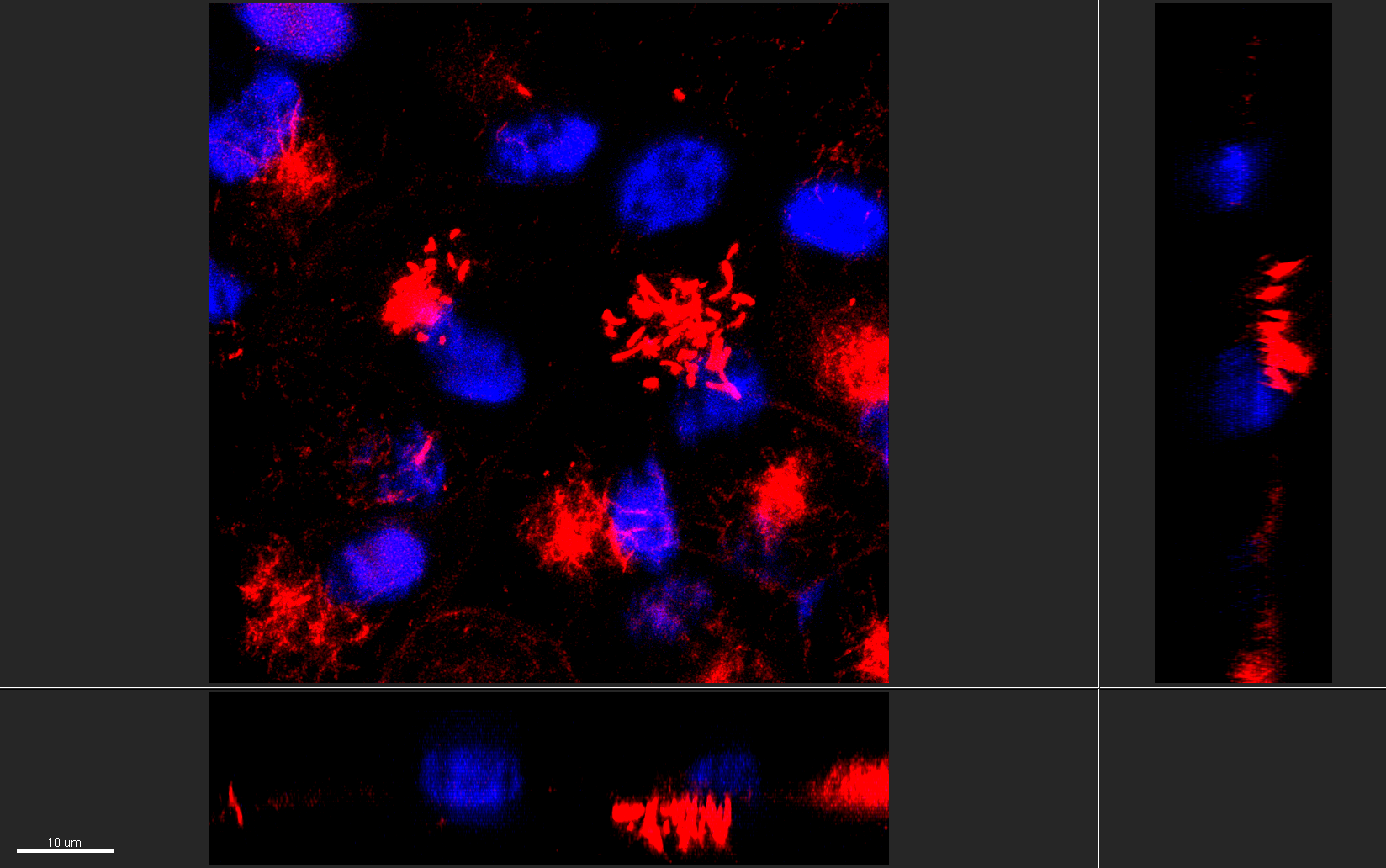

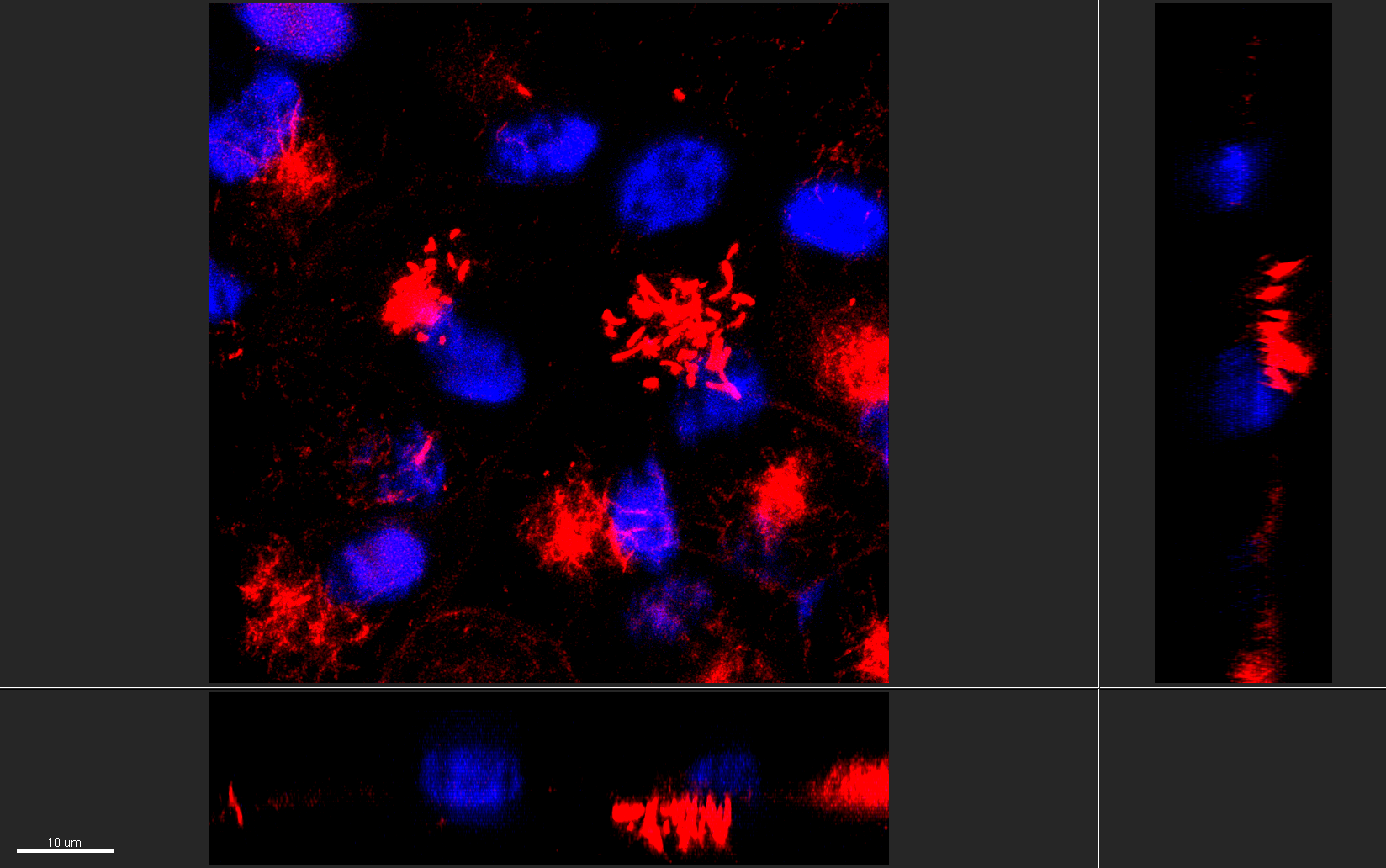


**α-acetylated tubulin / β-tubulin / Nuclei**


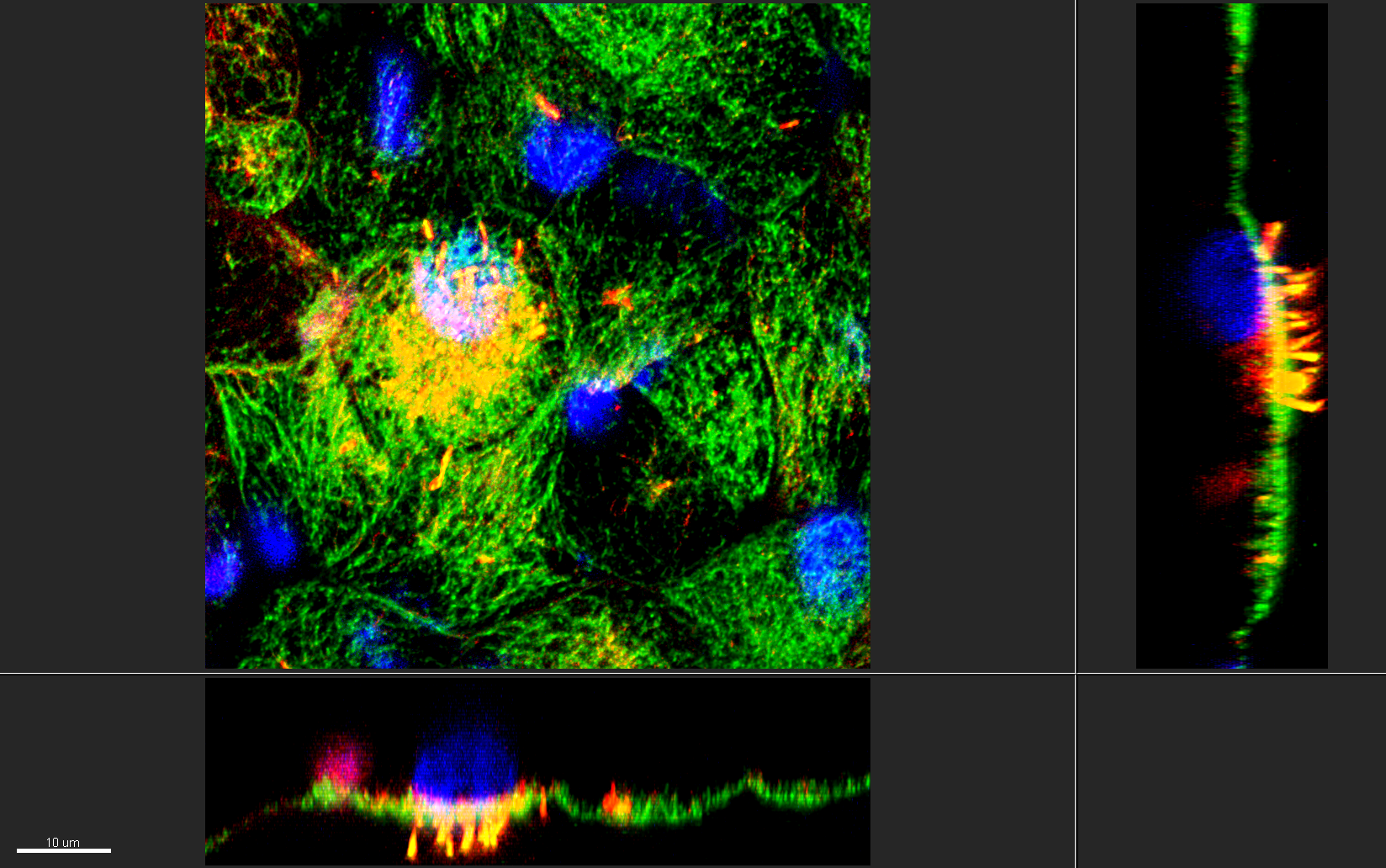

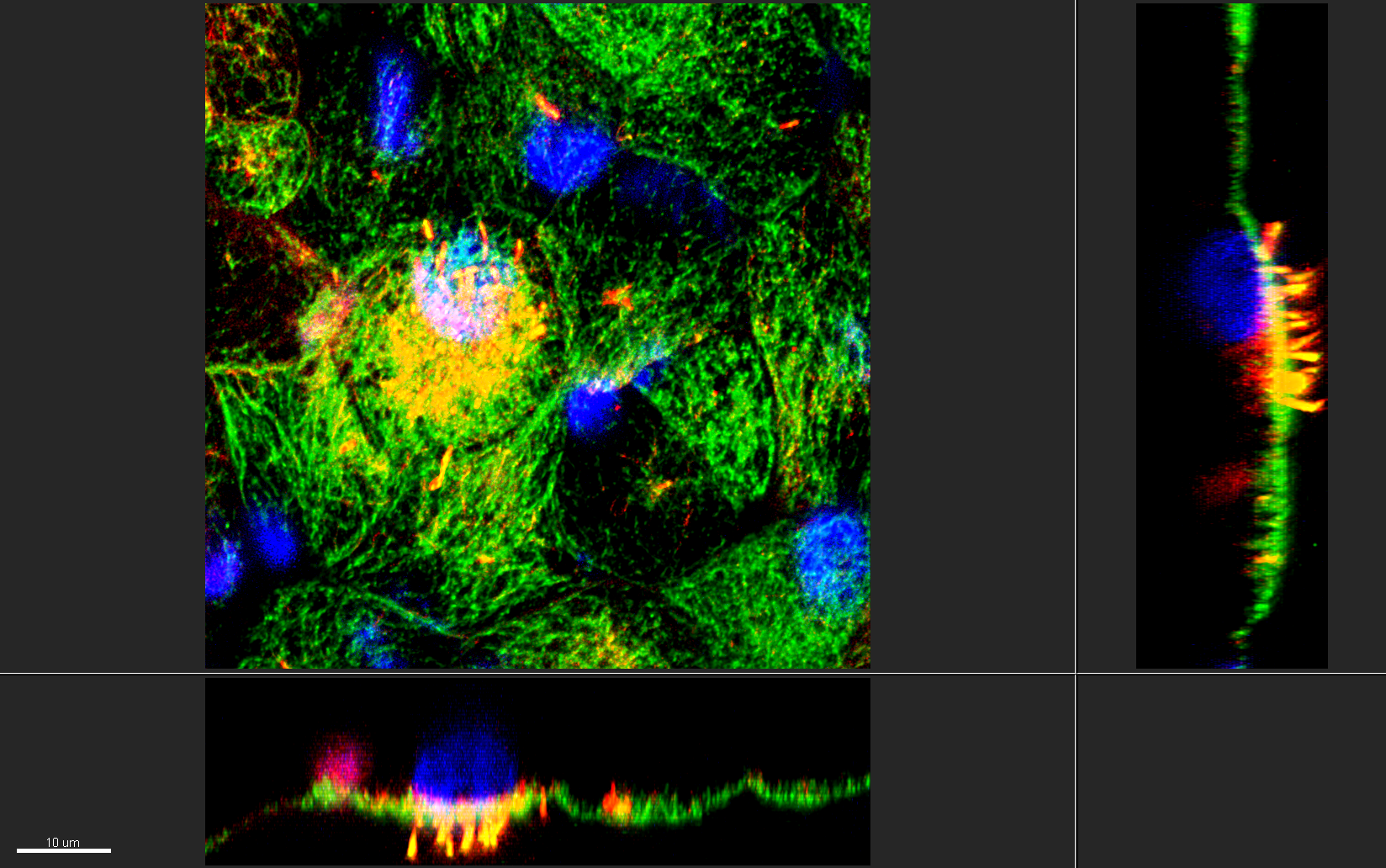

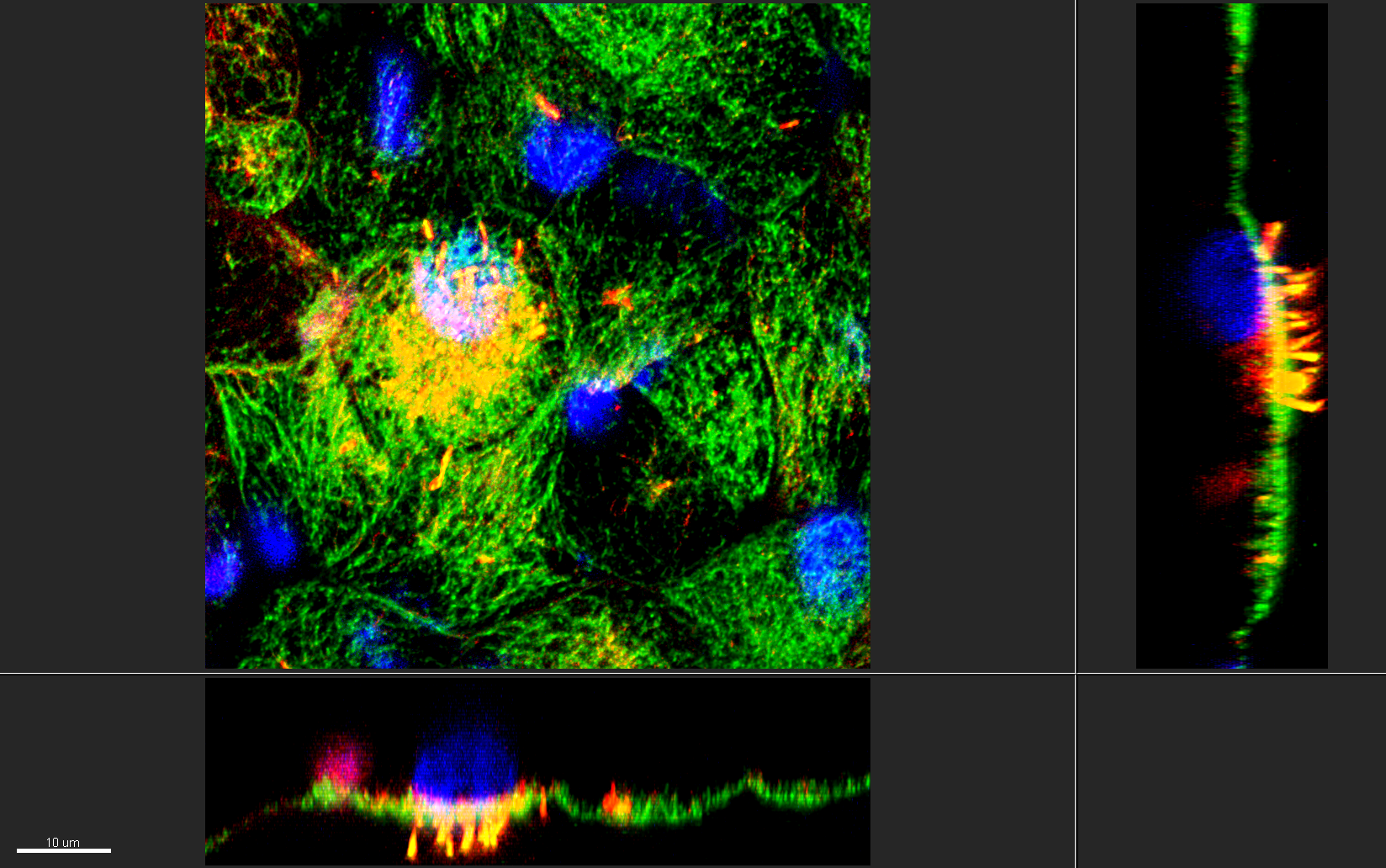

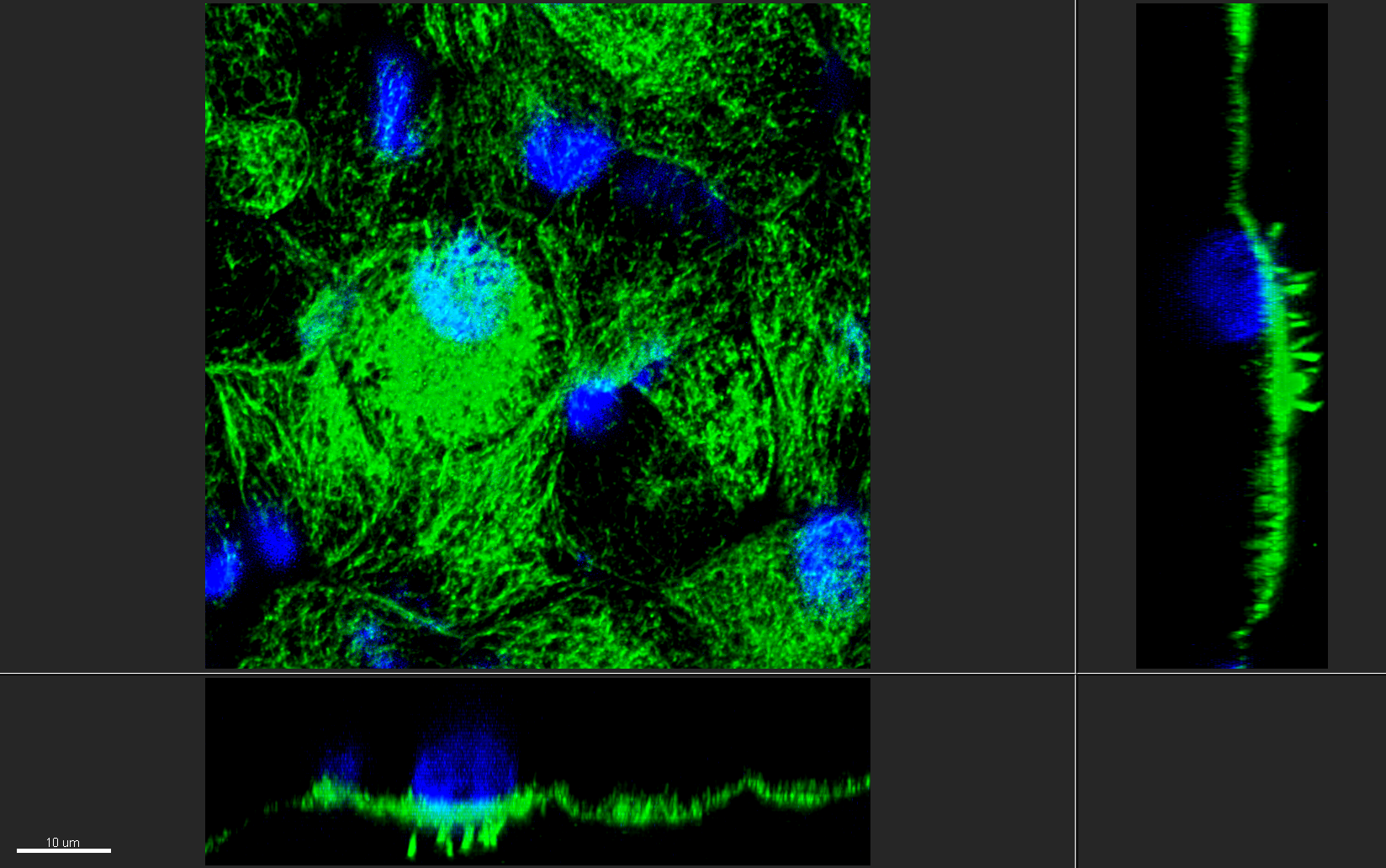

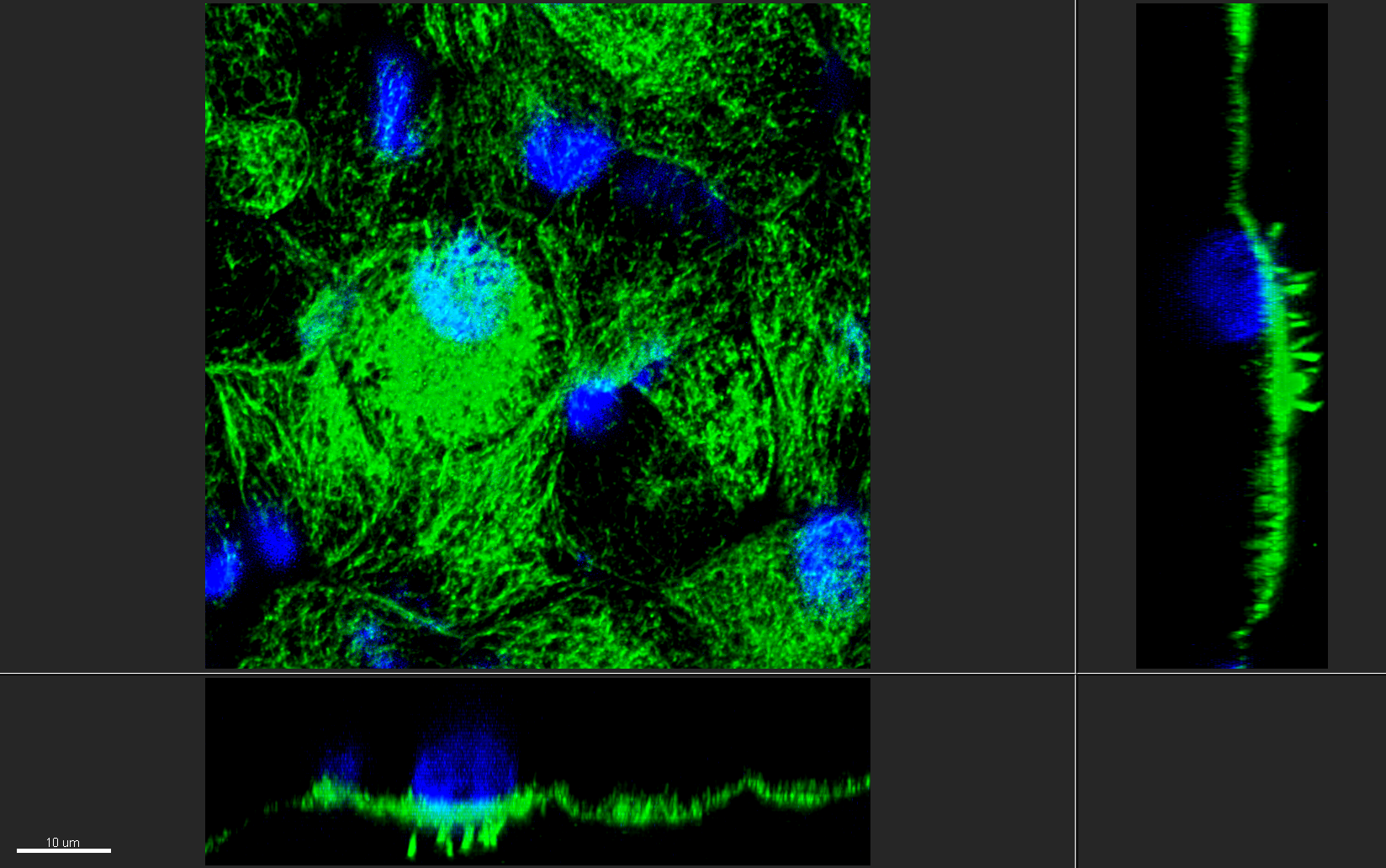

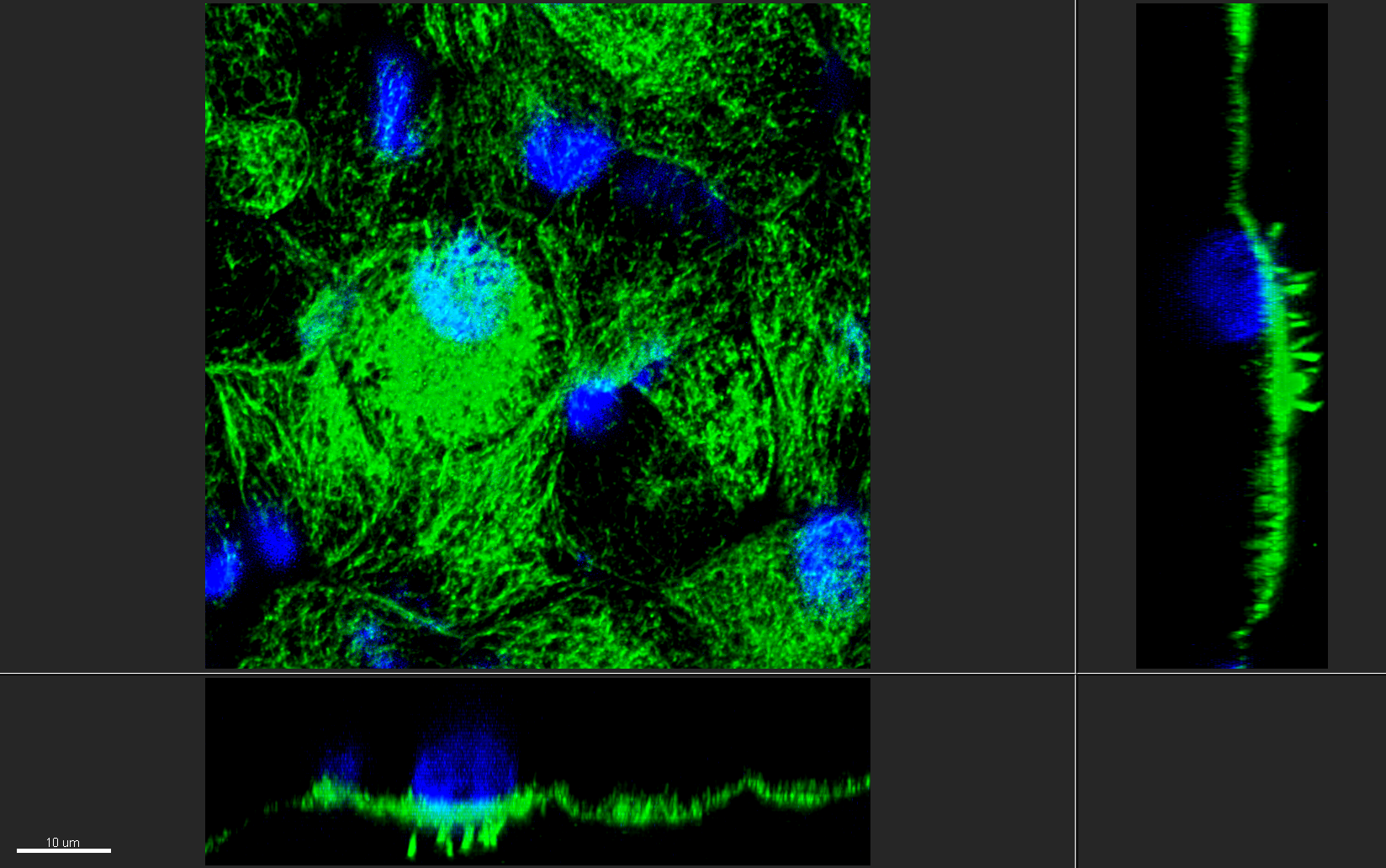

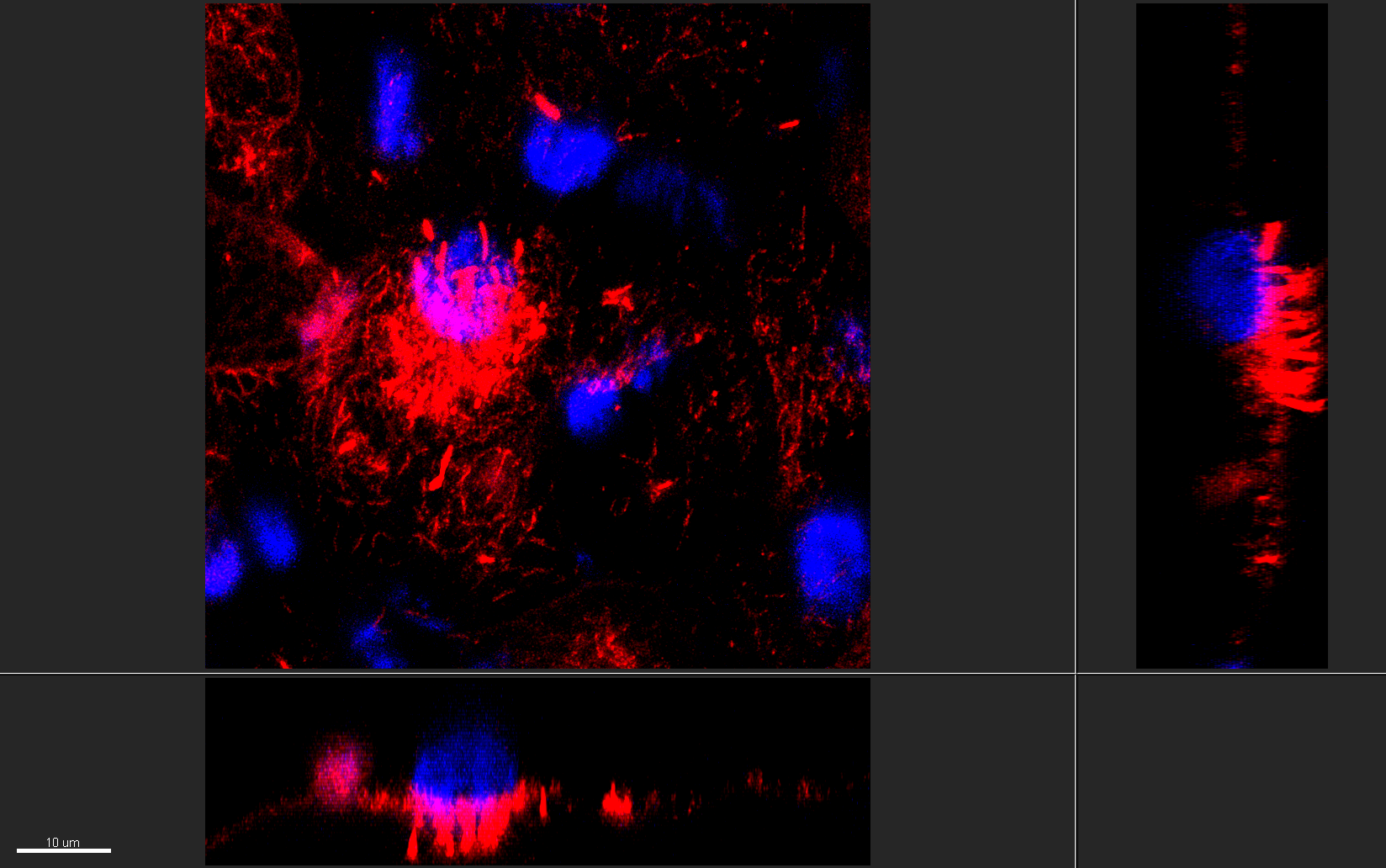

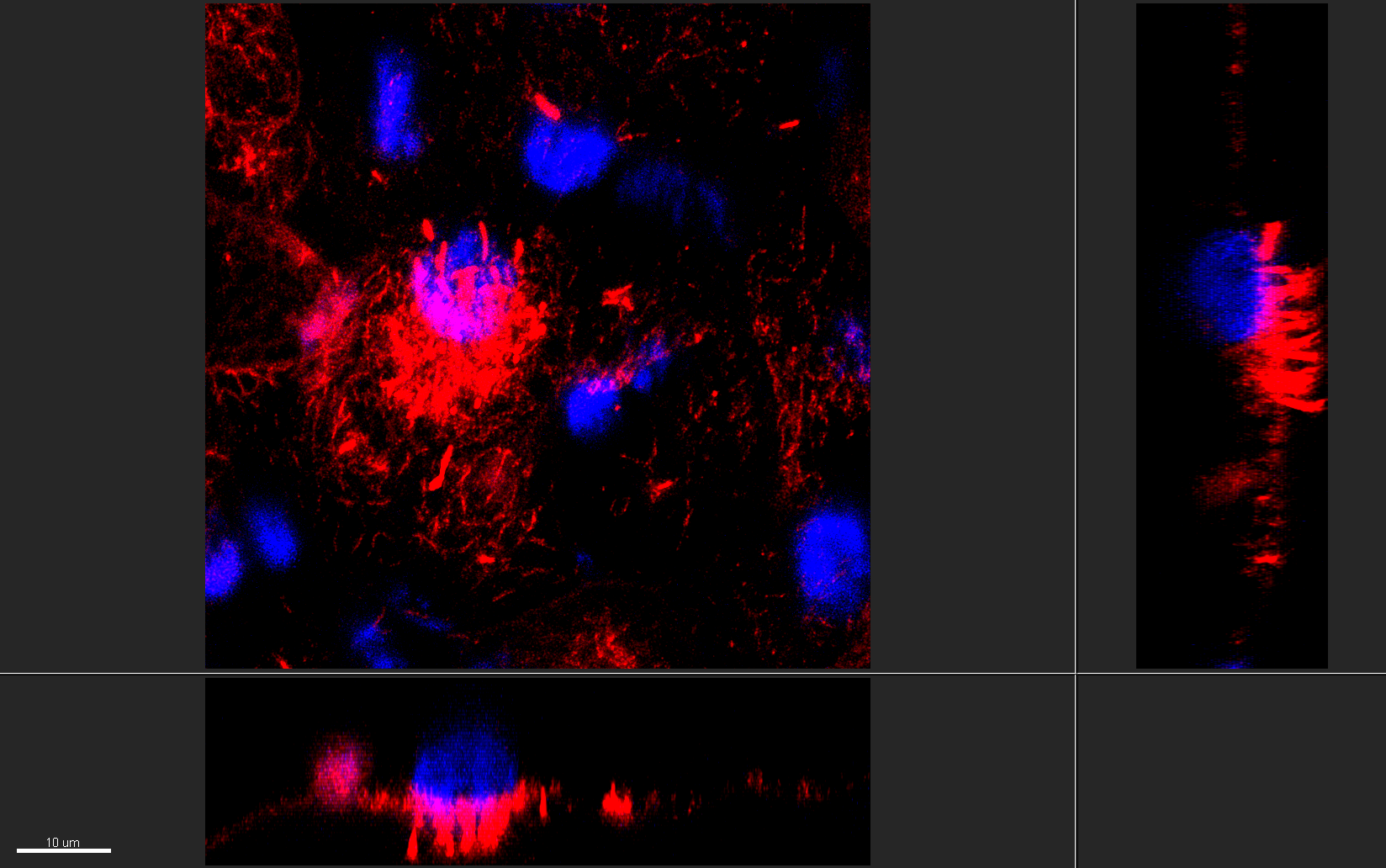


**b c**


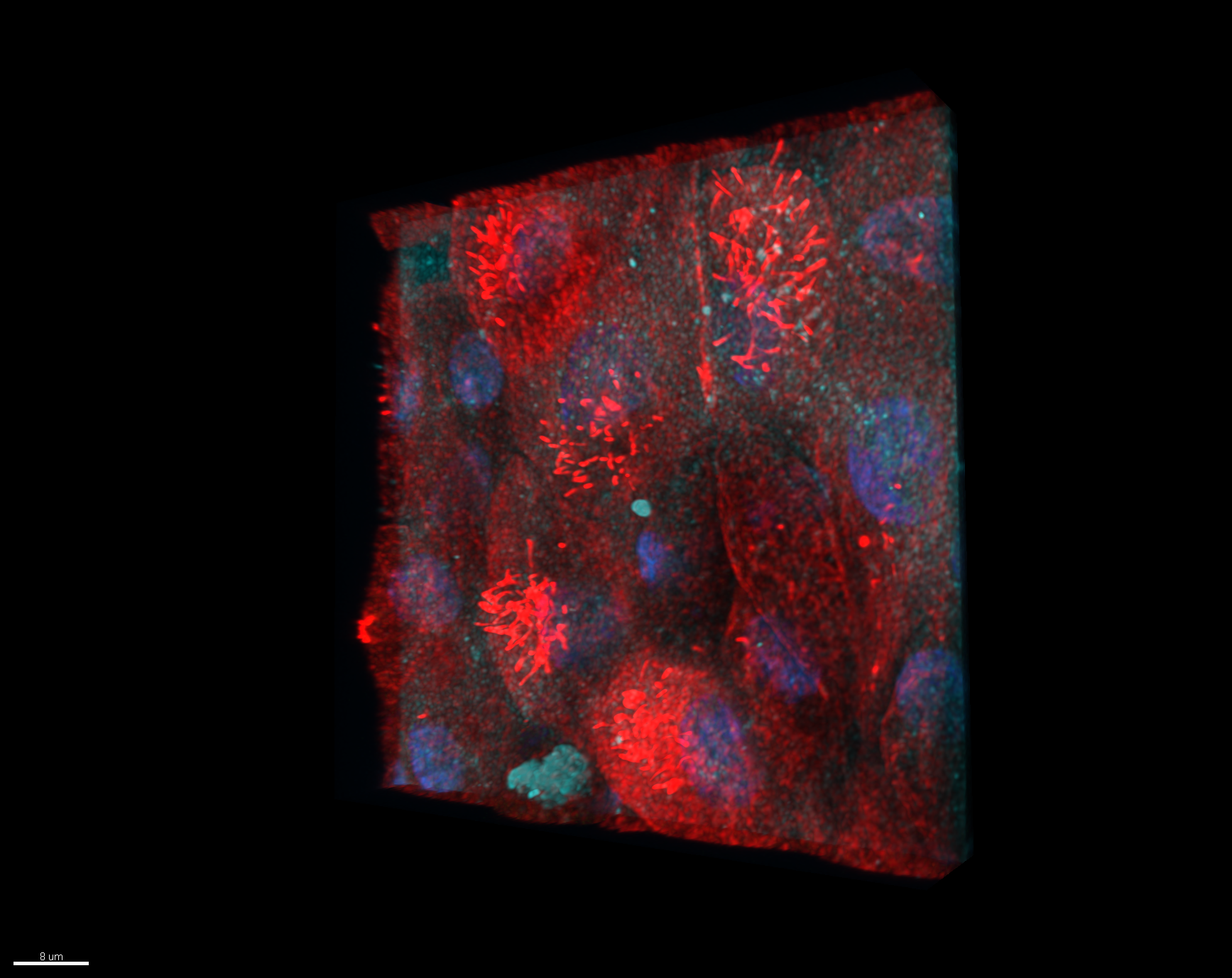


**β-tubulin / HtrA1 / Nuclei**

**
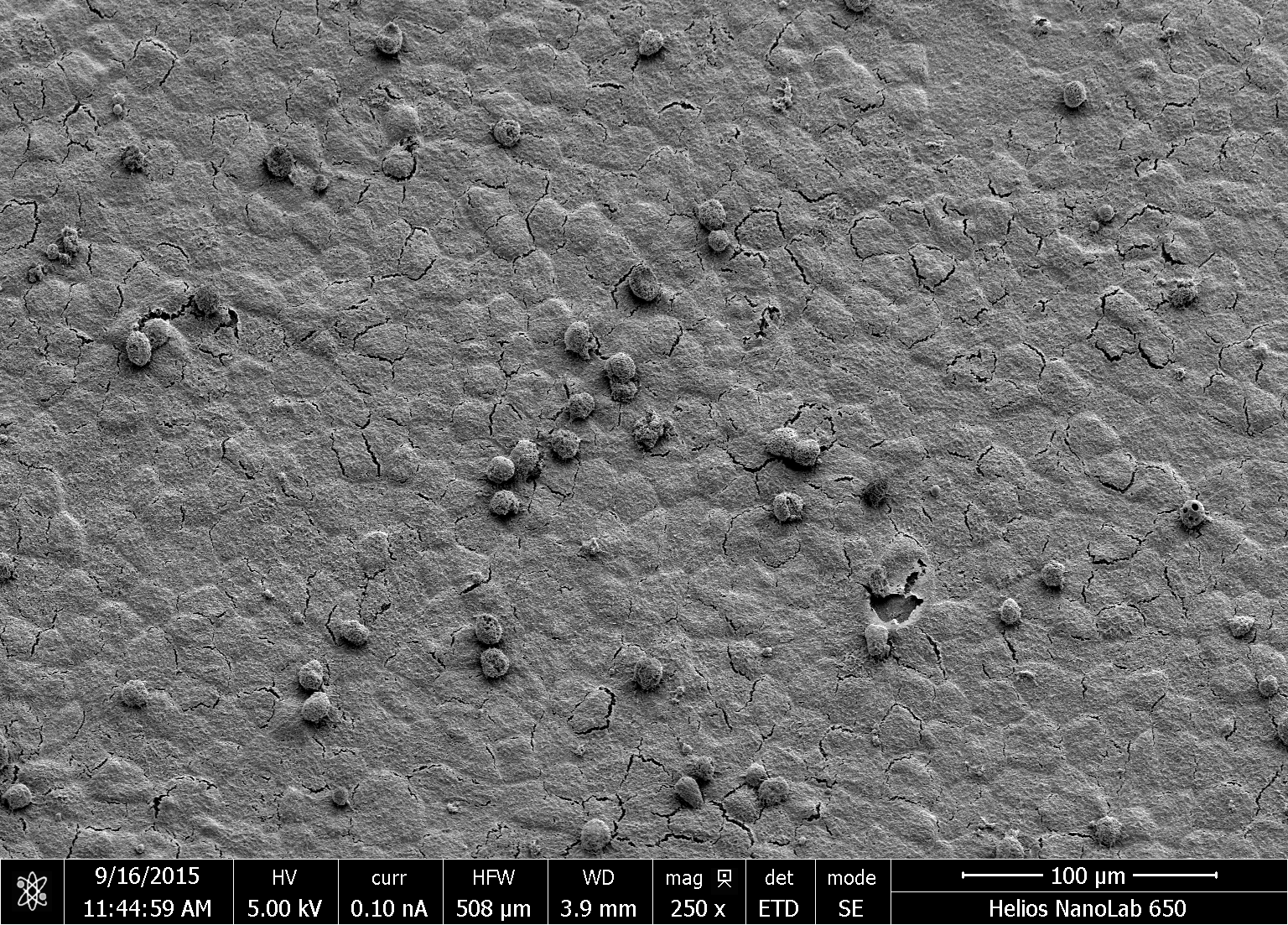
**


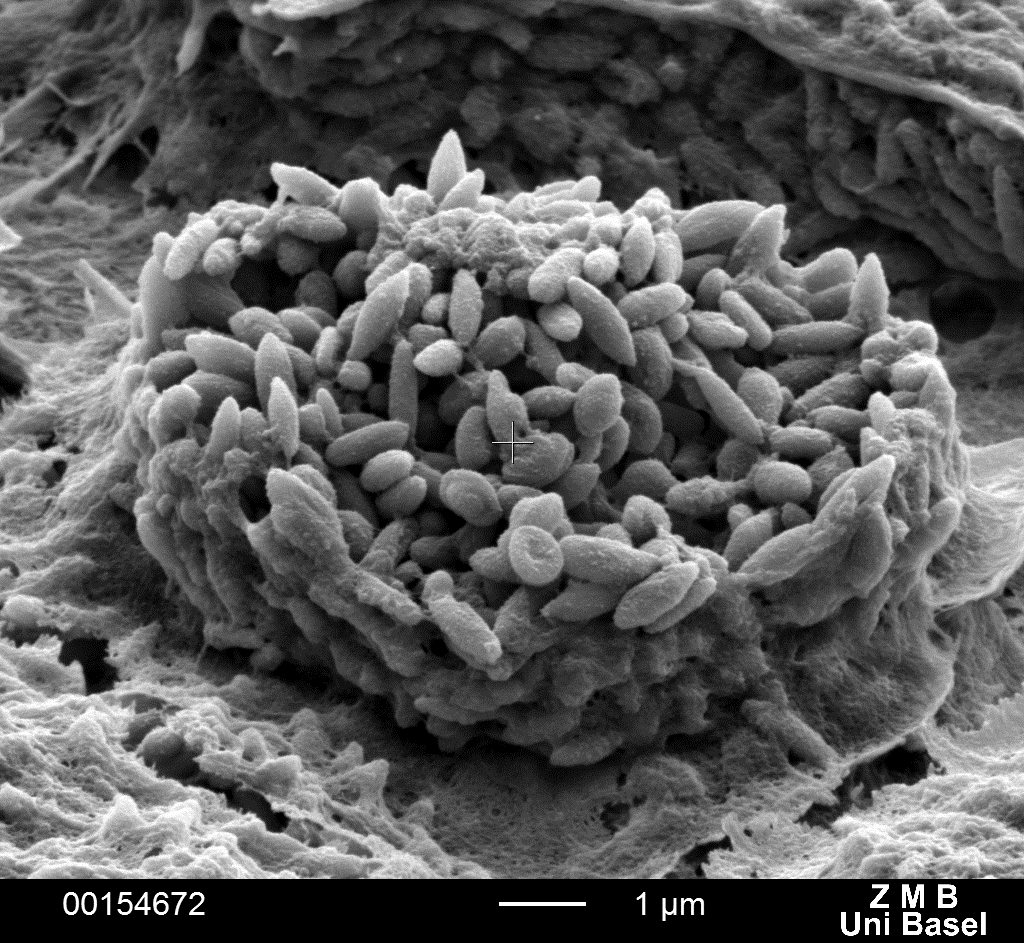


**d**

**
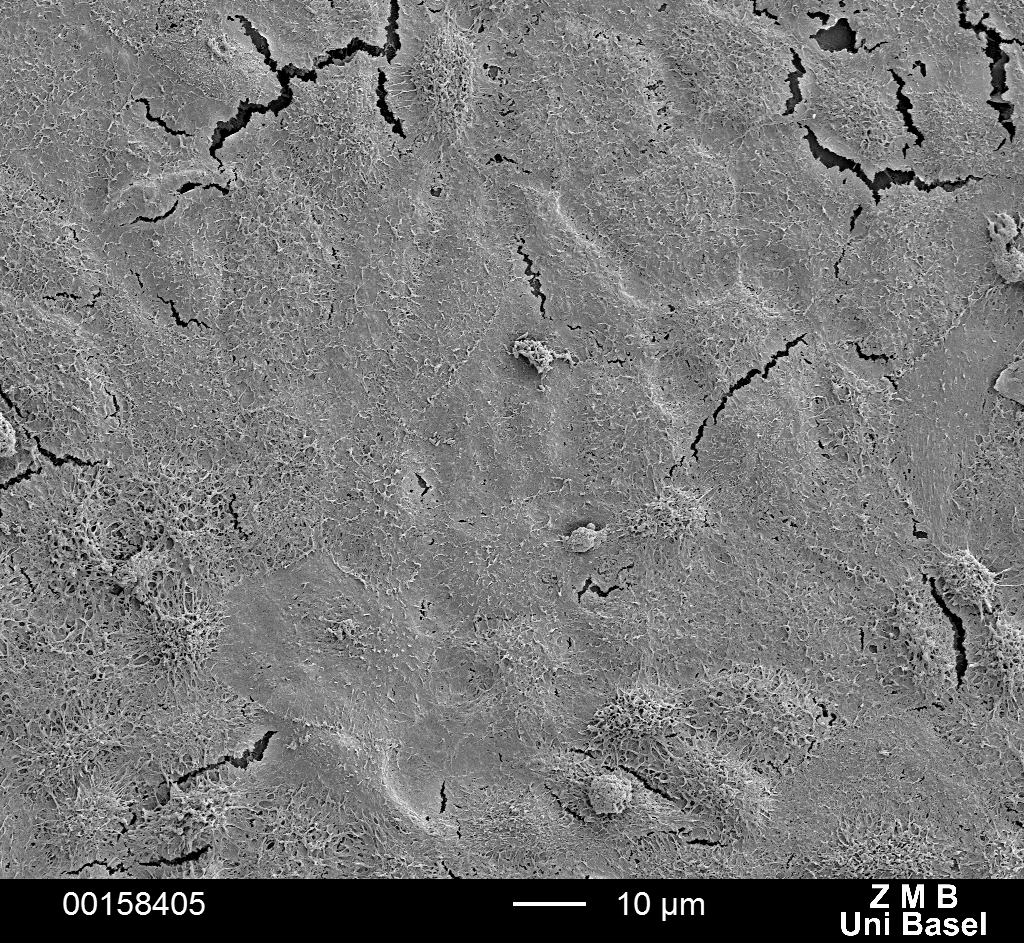

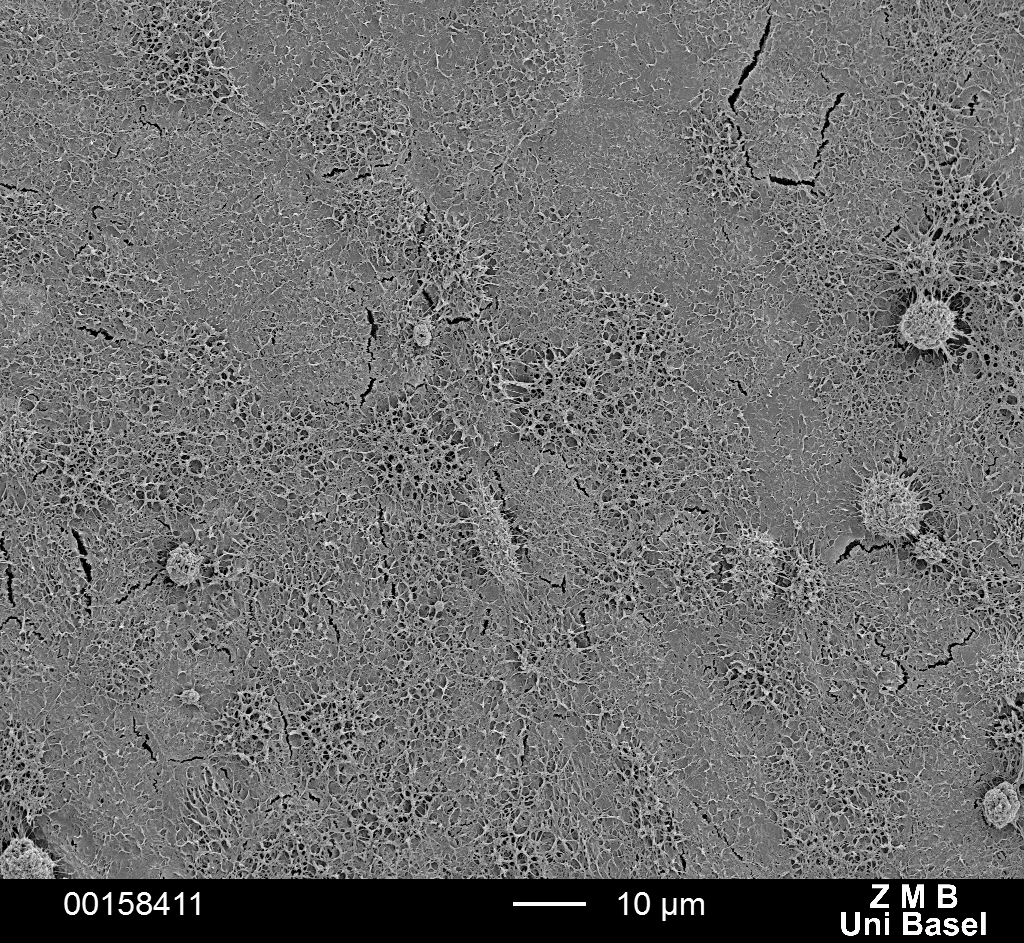
S328A HtrA1**

**Figure S5 – related to figure 6**


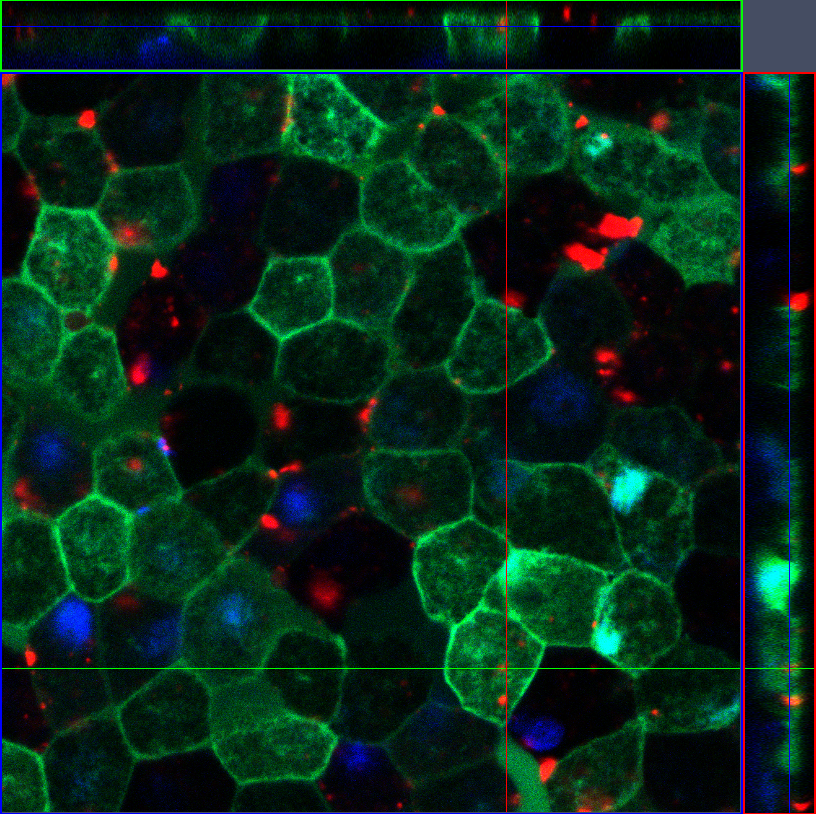


**Figure S6 – related to figure 7**

**a**

**
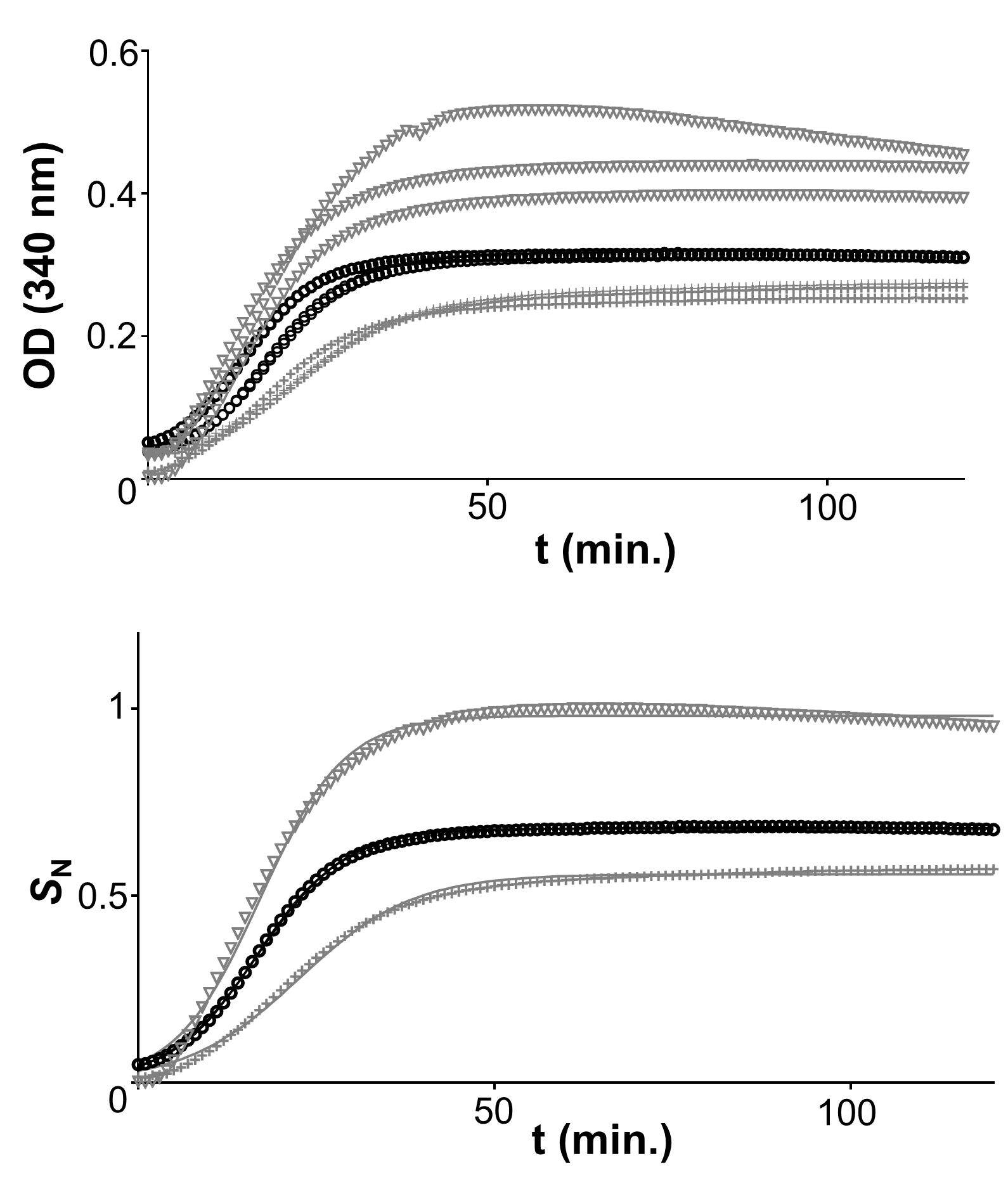

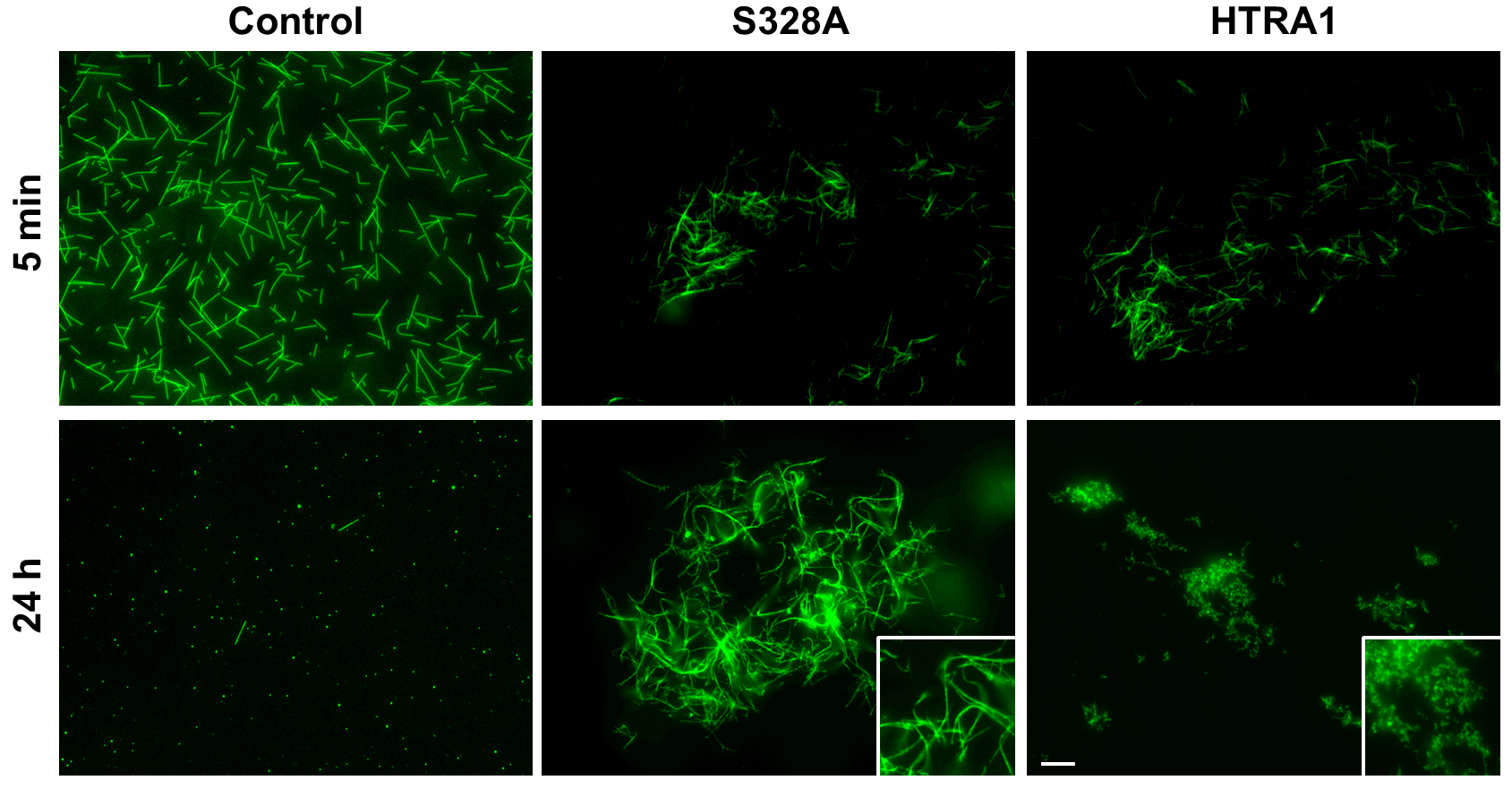
**

**
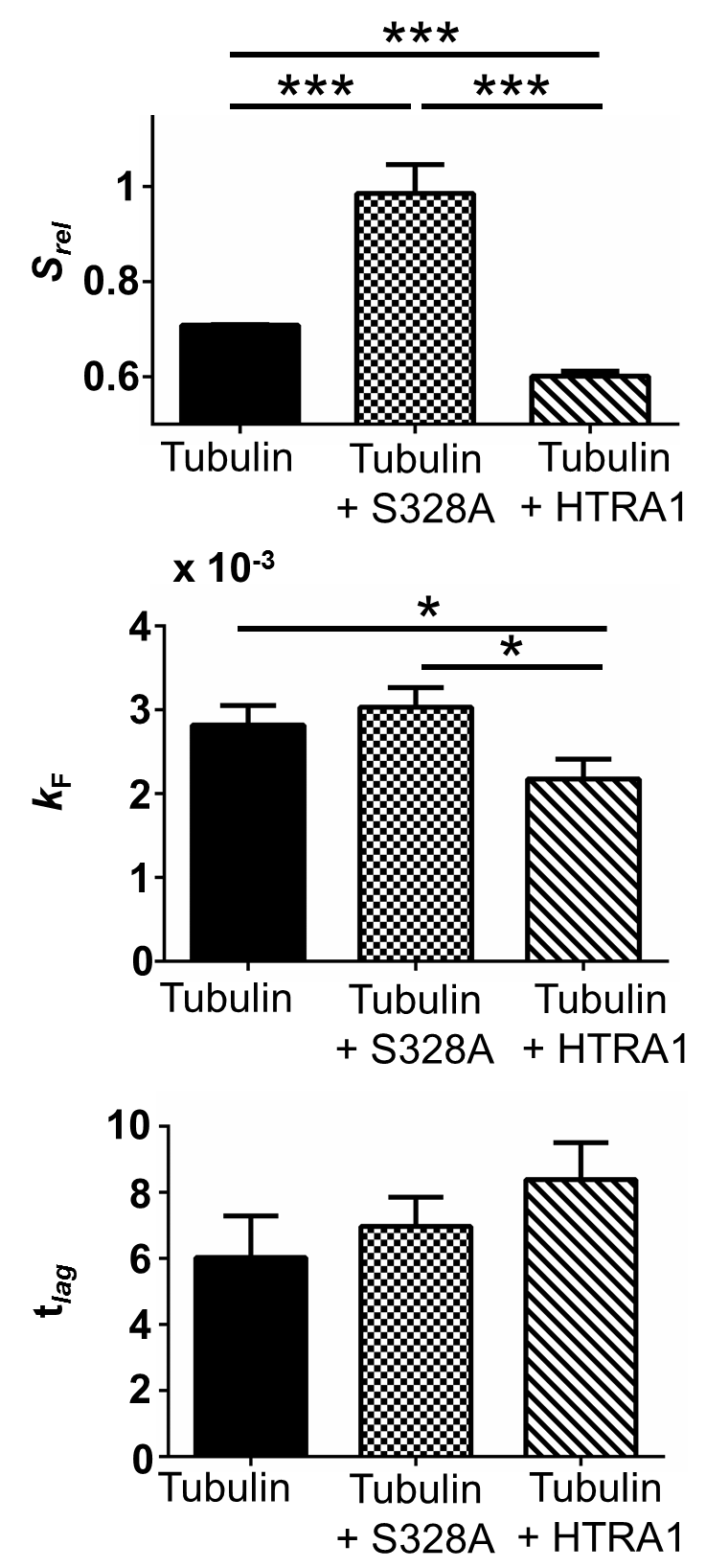
b c**

**Table S1: miRNA profiling. Related to figure S1**

| ID description | Specie | Citation | | log FC | log CPM | LR | P value | FDR |
| --- | --- | --- | --- | --- | --- | --- | --- | --- |
| hsa-miR-30a-5p | m | ([Soundara Pandi et al., 2013](#_ENREF_3)) | mmu-miR-30a-5p | -2.62 | 10.933 | 109.72 | 1.13E-25 | 2.41R-24 |
| hsa-miR-181a-5p | m | ([Ohana et al., 2015](#_ENREF_2)) | mmu-miR-181a-2 | 2.84 | 15.399 | 136.13 | 1.87E-31 | 5.67E-30 |
| hsa-miR-27a-3p | h | ([Wang et al., 2014](#_ENREF_4)) | hsa-miR-27a | -0.99 | 8.246 | 12.95 | 3.2E-04 | 8.21E-04 |
| hsa-miR-100-5p | h | ([Wang et al., 2014](#_ENREF_4)) | hsa-miR-100 | -0.54 | 11.197 | 12.296 | 4.54E-04 | 1.14E-03 |
| hsa-miR-204-5p | m/h | ([Li et al., 2012](#_ENREF_1)) | hsa-miR-204-5p | 1.73 | 19.240 | 130.44 | 3.29E-30 | 7.91E-29 |
|  |  | ([Soundara Pandi et al., 2013](#_ENREF_3)) | mmu-miR-204-5p |  |  |  |  |  |
|  |  | ([Ohana et al., 2015](#_ENREF_2)) | mmu-miR-204 |  |  |  |  |  |
| hsa-miR-23b-3p | h | ([Wang et al., 2014](#_ENREF_4)) | hsa-miR-23b | 0.66 | 8.97 | 15.23 | 9.51E-05 | 2.66E-04 |
| hsa-miR-99b-5p | m/h | ([Wang et al., 2014](#_ENREF_4)) | hsa-miR-99b-5p | 0.68 | 14.613 | 22.18 | 2.48E-06 | 8.88E-06 |
|  |  | ([Soundara Pandi et al., 2013](#_ENREF_3)) | mmu-miR-99b-5p |  |  |  |  |  |

miRNA identified in RPE cells at day 35 of culture. ID description, Species where it was reported to be previously identified (m, mouse and h, human), citation, LogFC (compared to day 7 of culture), lgCPM, LR, p value, FDR.

**Table S2: Htra1 co-immunoprecipitated proteins after statistical analysis of MS data (ranked by pAdj then signal reduction. Related to S2**

Several proteins were identified and quantified by profiling Htra1 interactome using ICC-MS in hRPE overexpressing Htra1. Besides proteins from the tubulin family, 9 proteins (C1QT5, ECHB, NPM, RS27L, PGAM5, COL12, DJC10, GCST and DNJA2) were identified as Htra1-interacting proteins with adjusted p-values below 1% and at least 2 unique peptides. pAdj: p value to test displacement adjusted by the Westfall-Young approach; Signal reduction: signal difference between the highest and the lowest concentration of competitor antibody; Unique peptides: number of peptides of a respective protein quantitation group.

**Table S3: NTAIL proteomics in compound treated RPE cells (control). Related to S2**

|  | | | **Fc=log2 (treated/control)** | | | |
| --- | --- | --- | --- | --- | --- | --- |
|  |  |  | **Compound A** | | **Compound B** | |
| **non prime** | **P1** | **prime** | **R1** | **R2** | **R1** | **R2** |
| AGKHVPRAVF | F | VDLEPTVIDEVR | -1.14 | -2.37 | -1.56 | -4.16 |
| YRQLFHPEQL | L | ITGKEDAANNYAR | -1.68 | -1.79 | -3.28 | -2.65 |
| TASLRFDGAL | L | NVDLTEFQTNLVPYPR | -2.46 | -1.68 | -5.97 | -9.97 |

Fold change (Fc) of down-regulated tubulin fragments under HtrA1 inhibitor treatment, lower panel. R1, replicate 1. R2, replicate 2.

**Supplemental figure legends**

**Figure S1 – related to figure 1**

1. Retinal histological sections immunostained against HtrA1 (pink) from four healthy subjects (left) and four AMD donors (right). Nuclei stained with hematoxylin.
2. Panels of genes found by RNA sequencing to be in consistently high expression (left panel) or consistently low expression (right panel) along the 35-day period of differentiation
3. Adenoviral design constructs. Left, S328 variant; right, HtrA1. Below, left graph indicating the % of GFP cells (or transfected cells) found in the S328A and HtrA1 overexpressing cells. Mean ±SD of two independent experiments, ***p < 0.001. Right graph, represents the TER values at the end of an experiment. Mean value (line) ±SD (shadow). HtrA1, black line; S328A. green line; Empty Vector, orange line; Contol cells purpule line.
4. Cellular experimental setup.
5. Intracellular detection of HtrA1 by Western blot, upper left panel. Intracellular detection of B-catenin and tubulin by Western blot, lower left panel. HtrA1 detection in the apical medium, upper central panel. PEDF detection in the apical medium, lower central panel. HtrA1 detection in the basolateral medium, right upper panel. PEDF detection in the basolateral medium, right lower panel.
6. Orthogonal projection of Na^+^K^+^ ATPase staining (green) where polarization of the cells can be observed. Nuclei (blue) stained with DAPI. Scale bar, 10 µm.

**Figure S2 – related to figure 2**

1. Immuno-Competitive Capture Mass Spectrometry (ICC-MS) applied for the detection of Htra1-interacting proteins**.** A pre-incubation of Htra1 overexpressing hRPE cell lysates with increasing concentration of Htra1 free antibody is performed prior to immunoprecipitation using the same Htra1 antibody immobilized on agarose beads. After several washes, the bound fractions are eluted from the beads and processed for protein identification by LC-MS/MS. Proteins are identified and then quantified according to their extracted-ion chromatograms. A robust statistical analysis is finally applied on each signal to derive concentration-dependent signal decrease of specific interactors.
2. Chemical formula and IC50 properties from the two compounds employed to inhibit HtrA1.
3. Three potential HtrA1 cleavage sites on tubulin, identified by N-terminomics.

**Figure S3 – related to figure 3**

1. IF detection for β-tubulin (red) HtrA1 (cyan) in control cells where an homogeneous tubulin staining can be found across the cells forming the monolayer. Scale bar, 20 µm.
2. Orthogonal projection of RPE cells overexpressing HtrA1. Primary cilia can be identified in some cells through the α-acetylated tubulin staining (red), white arrows. In some cases, the cytoplasmic tubulin has been degraded, remaining only the primary cilium (upper left cell). In the *xy* projection it can be observed how HtrA1 is appearing below the primary cilium, yellow arrow. Scale bar, 5 µm.
3. Orthogonal projection of RPE cells overexpressing HtrA1. Basal (left image) and apical (right image) from the same cells show how HtrA1 (cyan) is mostly found at the basolateral side of the cells, but not at the level of the tight junctions where claudin-19 is located (red). Nuclei were stained with DPAI (blue). Scale bar, 5 µm.
4. Transmission electron micrographs from immune-gold detection of HtrA1 in different overexpressing cells. Scale bars, 500nm and 200nm. Red arrows indicate gold localization. Dashed line indicates the limit between cells.

**Figure S4 – related to figure 5**

1. Orthogonal projections from two areas where multicilated cells were found. Cells were positive for α-acetylated tubulin (green) and β-tubulin (red). The *xy* projection illustrates how these structures are projected from the apical membrane of the cells. Scale bar, 5 µm.
2. 3D render from an area where cilia protruding from the cells can be observed in cells overexpressing S328A. β-tubulin (red), HtrA1 (cyan), Nuclei (blue). Scale bar, 8 µm.
3. Multiciliated cells imaged by scanning electron microscopy. Scale bar, 1 µm.
4. Scanning electron micrograph of the RPE surface from S328A (left) and HtrA1 (right) overexpressing cells.

**Figure S5 – related to figure 6**

Orthogonal projection from RPE cells where an OS (red) can be observed (yellow arrows). Cells overexpressing HtrA1 are GFP positive (green). Nuclei were stained with Hoechst (blue). Scale bar, 10 µm.

**Figure S6 – related to figure 7**

1. Microtubules polymerized *in vitro* and incubated with S328A HtrA1 (center) and HtrA1 (right). Images acquired 5 min and 24h after adding the protease. Scale bar, 10 µm.
2. Microtubule polymerization with taxol in the absence or presence of S328A HtrA1 and HtrA1. The absorbance at 340 nm was monitored *in vitro* as a function of time for three independent replicates (upper panel). The average signal for each variant was calculated and normalized relative to the overall maximum. Best fits to sigmoidal functions are shown with solid lines (lower panel).
3. Comparison of the growth amplitudes (*S_rel_*) (upper panel), kinetic rate constants (*k*_F_) (central panel) and nucleation times (t*_lag_*) (lower panel) of microtubule polymerization reaction with taxol in the absence or presence of S328A HtrA1 and HtrA1. Mean ±SD for three replicates are reported. *p < 0.05, **p < 0.01, ***p < 0.001.

**References**

Li, W.B., Zhang, Y.S., Lu, Z.Y., Dong, L.J., Wang, F.E., Dong, R., and Li, X.R. (2012). Development of retinal pigment epithelium from human parthenogenetic embryonic stem cells and microRNA signature. Investigative ophthalmology & visual science *53*, 5334-5343.

Ohana, R., Weiman-Kelman, B., Raviv, S., Tamm, E.R., Pasmanik-Chor, M., Rinon, A., Netanely, D., Shamir, R., Solomon, A.S., and Ashery-Padan, R. (2015). MicroRNAs are essential for differentiation of the retinal pigmented epithelium and maturation of adjacent photoreceptors. Development (Cambridge, England) *142*, 2487-2498.

Soundara Pandi, S.P., Chen, M., Guduric-Fuchs, J., Xu, H., and Simpson, D.A. (2013). Extremely complex populations of small RNAs in the mouse retina and RPE/choroid. Investigative ophthalmology & visual science *54*, 8140-8151.

Wang, H.C., Greene, W.A., Kaini, R.R., Shen-Gunther, J., Chen, H.I., Cai, H., and Wang, Y. (2014). Profiling the microRNA Expression in Human iPS and iPS-derived Retinal Pigment Epithelium. Cancer informatics *13*, 25-35.
